# Molecular phylogenetics of cool-season grasses in the subtribes Agrostidinae, Anthoxanthinae, Aveninae, Brizinae, Calothecinae, Koeleriinae and Phalaridinae (Poaceae, Pooideae, Poeae, Poeae chloroplast group 1)

**DOI:** 10.3897/phytokeys.87.12774

**Published:** 2017-10-09

**Authors:** Jeffery M. Saarela, Roger D. Bull, Michel J. Paradis, Sharon N. Ebata, Paul M. Peterson, Robert J. Soreng, Beata Paszko

**Affiliations:** 1 Botany Section, Research and Collections, Canadian Museum of Nature, Ottawa, Ontario, Canada; 2 Department of Botany, National Museum of Natural History, Smithsonian Institution, Washington, DC, United States of America; 3 Department of Vascular Plant Systematics and Phytogeography, W. Szafer Institute of Botany, Polish Academy of Sciences, Kraków, Poland

**Keywords:** grasses, phylogenetics, ETS, systematics, taxonomy, classification

## Abstract

Circumscriptions of and relationships among many genera and suprageneric taxa of the diverse grass tribe Poeae remain controversial. In an attempt to clarify these, we conducted phylogenetic analyses of >2400 new DNA sequences from two nuclear ribosomal regions (ITS, including internal transcribed spacers 1 and 2 and the 5.8S gene, and the 3’-end of the external transcribed spacer (ETS)) and five plastid regions (*matK*, *trnL–trnF*, *atpF–atpH*, *psbK–psbI*, *psbA–rps19–trnH*), and of more than 1000 new and previously published ITS sequences, focused particularly on Poeae chloroplast group 1 and including broad and increased species sampling compared to previous studies. Deep branches in the combined plastid and combined ITS+ETS trees are generally well resolved, the trees are congruent in most aspects, branch support across the trees is stronger than in trees based on only ITS and fewer plastid regions, and there is evidence of conflict between data partitions in some taxa. In plastid trees, a strongly supported clade corresponds to Poeae chloroplast group 1 and includes Agrostidinae p.p., Anthoxanthinae, Aveninae s.str., Brizinae, Koeleriinae (sometimes included in Aveninae s.l.), Phalaridinae and Torreyochloinae. In the ITS+ETS tree, a supported clade includes these same tribes as well as Sesleriinae and Scolochloinae. Aveninae s.str. and Sesleriinae are sister taxa and form a clade with Koeleriinae in the ITS+ETS tree whereas Aveninae s.str. and Koeleriinae form a clade and Sesleriinae is part of Poeae chloroplast group 2 in the plastid tree. All species of *Trisetum* are part of Koeleriinae, but the genus is polyphyletic. Koeleriinae is divided into two major subclades: one comprises *Avellinia*, *Gaudinia*, *Koeleria*, *Rostraria*, *Trisetaria* and *Trisetum* subg. Trisetum, and the other *Calamagrostis*/*Deyeuxia* p.p. (multiple species from Mexico to South America), *Peyritschia*, *Leptophyllochloa*, *Sphenopholis*, *Trisetopsis* and *Trisetum* subg. *Deschampsioidea. Graphephorum*, *Trisetum cernuum*, *T. irazuense* and *T. macbridei* fall in different clades of Koeleriinae in plastid vs. nuclear ribosomal trees, and are likely of hybrid origin. ITS and *matK* trees identify a third lineage of Koeleriinae corresponding to Trisetum subsect. Sibirica, and affinities of *Lagurus ovatus* with respect to Aveninae s.str. and Koeleriinae are incongruent in nuclear ribosomal and plastid trees, supporting recognition of *Lagurus* in its own subtribe. A large clade comprises taxa of Agrostidinae, Brizinae and Calothecinae, but neither Agrostidinae nor Calothecinae are monophyletic as currently circumscribed and affinities of Brizinae differ in plastid and nuclear ribosomal trees. Within this clade, one newly identified lineage comprises *Calamagrostis coarctata*, *Dichelachne*, *Echinopogon* (Agrostidinae p.p.) and *Relchela* (Calothecinae p.p.), and another comprises *Chascolytrum* (Calothecinae p.p.) and *Deyeuxia effusa* (Agrostidinae p.p.). Within Agrostidinae p.p., the type species of *Deyeuxia* and *Calamagrostis* s.str. are closely related, supporting classification of *Deyeuxia* as a synonym of *Calamagrostis* s.str. Furthermore, the two species of *Ammophila* are not sister taxa and are nested among different groups of *Calamagrostis* s.str., supporting their classification in *Calamagrostis*. *Agrostis*, *Lachnagrostis* and *Polypogon* form a clade and species of each are variously intermixed in plastid and nuclear ribosomal trees. Additionally, all but one species from South America classified in Deyeuxia sect. Stylagrostis resolve in Holcinae p.p. (*Deschampsia*). The current phylogenetic results support recognition of the latter species in *Deschampsia*, and we also demonstrate *Scribneria* is part of this clade. Moreover, Holcinae is not monophyletic in its current circumscription because *Deschampsia* does not form a clade with *Holcus* and *Vahlodea*, which are sister taxa. The results support recognition of *Deschampsia* in its own subtribe Aristaveninae. Substantial further changes to the classification of these grasses will be needed to produce generic circumscriptions consistent with phylogenetic evidence. The following 15 new combinations are made: Calamagrostis × calammophila, *C. breviligulata*, C. breviligulata subsp. champlainensis, C. × don-hensonii, *Deschampsia aurea*, *D. bolanderi*, *D. chrysantha*, D. chrysantha var. phalaroides, *D. eminens*, D. eminens var. fulva, D. eminens var. inclusa, *D. hackelii*, *D. ovata*, and *D. ovata* var. *nivalis. D. podophora*; the new name *Deschampsia parodiana* is proposed; the new subtribe Lagurinae is described; and a second-step lectotype is designated for the name *Deyeuxia phalaroides*.

## Introduction

The cool-season grass subfamily Pooideae is one of three subfamilies comprising the BOP clade (Bambusoideae, Oryzoideae (=Ehrhartoideae), Pooideae) and the largest of the 12 grass subfamilies. It includes ca. 4200 species in 197 genera (Soreng et al. 2015b). Economically important species in the subfamily include temperate cereals such as wheat (*Triticum* L.), barley (*Hordeum* L.) and oats (*Avena* L.), numerous turfgrasses in the genera *Agrostis* L., *Festuca* L., *Lolium* L. and *Poa* L., and important pasture and wild forage grasses (e.g., *Alopecurus* L., *Dactylis* L., *Elymus* L., *Phleum* L.). Phylogenetic analyses have identified numerous major lineages of Pooideae (Macfarlane and Watson 1980, 1982, Soreng et al. 1990, 2007, Catalán et al. 2004, Davis and Soreng 2007, Quintanar et al. 2007, Schneider et al. 2009, 2012, Saarela et al. 2010) classified as supertribes, tribes and subtribes (Clayton and Renvoize 1986, Watson and Dallwitz 1992 onwards, Grass Phylogeny Working Group 2001, Soreng et al. 2003, 2007, Schneider et al. 2009, 2011). Recent classifications recognize 14 (Soreng et al. 2015b) and 10 tribes (Kellogg 2015) in Pooideae. The largest of these is tribe Poeae R. Br., part of a large clade including tribes Brachypodieae, Bromeae, Littledaleeae and Triticeae (e.g., Soreng et al. 2007, 2015b, Saarela et al. 2015). Poeae includes ca. 2776 species in 118 genera distributed in cool-temperate, Mediterranean and Arctic climates (Soreng et al. 2015b). Taxa now recognized in Poeae were previously included in numerous smaller tribes and subtribes, including the Aveneae (the oat tribe) and the Poeae *sensu stricto* (s.str.), recognized based on morphological characteristics (Clayton and Renvoize 1986, Quintanar et al. 2007), but neither Aveneae nor Poeae s.str. are monophyletic in their traditional circumscriptions.

Phylogenetic analyses of plastid DNA have identified two major clades in Poeae (Soreng and Davis 2000, Döring et al. 2007, Quintanar et al. 2007, Schneider et al. 2009, Grass Phylogeny Working Group II 2012, Saarela et al. 2015). Soreng and Davis (2000) initially described these clades as “taxa with Aveneae-type plastid DNA” and “taxa with Poeae-type plastid DNA”, because most taxa in each clade were traditionally recognized in Aveneae or Poeae s.str. These clades have since been referred to informally in various ways, often labelled “1” or “2”, with “1” always referring to the clade with Aveneae-type plastid DNA and “2” always referring to the clade with Poeae-type DNA. The variations include “Poeae subclade 1” and “Poeae subclade 2” (Davis and Soreng 2007), “Plastid Group 1 (‘Aveneae-type’)” and “Plastid Group 2 (‘Poeae-type’)” (Soreng et al. 2007), “Poeae chloroplast group 1 (Aveneae type)” and “Poeae chloroplast group 2 (Poeae type)” (Soreng et al. 2015b), and “Poeae chloroplast group 1” and “Poeae chloroplast group 2” (Schneider et al. 2009, Saarela et al. 2015). We use the latter terminology here.

Several subtribes are recognized in Poeae chloroplast groups 1 and 2. Poeae chloroplast group 1 comprises seven subtribes: Agrostidinae Fr., Anthoxanthinae A. Gray, Aveninae J. Presl, Brizinae Tzvelev, Calothecinae Soreng, Phalaridinae Fr. and Torreyochloinae Soreng & J. I. Davis (Soreng et al. 2015b). Of these, all but Agrostidinae and Aveninae include only one or two genera. Anthoxanthinae comprises the genus *Anthoxanthum* L. (=*Hierochloe* R. Br.) (Pimentel et al. 2013), Phalaridinae the genus *Phalaris* L., Brizinae the genera *Airopsis* Desv. and *Briza* L., and Torreyochloinae the genera *Amphibromus* Nees and *Torreyochloa* Nees. The recently recognized subtribe Calothecinae (Soreng et al. 2015b) comprises *Relchela* Steud. and *Chascolytrum* Desv. *sensu lato* (s.l.). *Chascolytrum* s.l. now includes species previously treated in *Calotheca* Desv., *Gymnachne* Parodi, *Erianthecium* Parodi, *Lombardochloa* Roseng. & B.R. Arrill, *Microbriza* Parodi ex Nicora & Rúgolo, *Pooidium* Nees and *Rhombolytrum* Link (Essi et al. 2008, 2011). *Chascolytrum* s.l. was previously classified in Brizinae (Soreng et al. 2007), but Brizinae in this circumscription is not monophyletic (Davis and Soreng 2007, Döring et al. 2007, Quintanar et al. 2007, Soreng et al. 2007, Saarela et al. 2010, Grass Phylogeny Working Group II 2012). Many aspects of generic circumscription and relationship among and within the subtribes of Poeae chloroplast group 1 are unresolved. Moreover, in trees based on nuclear ribosomal DNA (nrDNA), subtribes Sesleriinae Parl. and Scolochloinae Tzvelev, which are part of Poeae chloroplast group 2, are closely related to taxa of Poeae chloroplast group 1 (Quintanar et al. 2007, Gillespie et al. 2008, Saarela et al. 2010, Soreng et al. 2015b), suggesting the possibility of an ancient hybrid origin of these lineages. The other subtribes recognized in Poeae chloroplast group 2 are Airinae Fr., Ammochloinae Tzvelev, Coleanthinae Rouy, Cynosurinae Fr., Dactylidinae Stapf, Holcinae Dumort., Loliinae Dumort., Miliinae Dumort., Parapholiinae Caro and Poinae Dumort. (Soreng et al. 2015b).

Aveninae and Agrostidinae are the most species-rich subtribes of Poeae chloroplast group 1. Aveninae comprises ca. 18 genera and ca. 300 species (Soreng et al. 2015b), although several genera and subtribal alignments remain problematic. Genera include *Arrhenatherum* P. Beauv., *Avellinia* Parl., *Avena* L., *Calamagrostis*/*Deyeuxia* p.p., *Gaudinia* P. Beauv., *Graphephorum* Desv., *Helictotrichon* Besser s.str., *Koeleria* Pers., *Lagurus* L., *Leptophyllochloa* C.E. Calderón, *Peyritschia* E. Fourn., *Pseudarrhenatherum* Rouy, *Rostraria* Trin., *Sphenopholis* Scribn., *Trisetaria* Forssk., *Tricholemma* (Röser) Röser, *Trisetopsis* Röser & Wölk, *Trisetum* Pers., *Tzveleviochloa* Röser & A. Wölk and ×*Trisetopsotrichon* Röser & A. Wölk. Of these, the best studied genus is *Avena* (oats) (Drossou et al. 2004, Peng et al. 2008, 2010a, 2010b, Li et al. 2009, Yan et al. 2014). Several studies have identified *Helictotrichon* s.l. (as traditionally defined) as paraphyletic or polyphyletic (Grebenstein et al. 1998, Röser et al. 2001, Quintanar et al. 2007, Wölk and Röser 2014, 2017), and it has been divided into the genera *Avenula* (Dumort.) Dumort., *Helictochloa* Romero Zarco, *Helictotrichon* s.str., *Tricholemma*, *Trisetopsis*, ×*Trisetopsotrichon* and *Tzveleviochloa* (Röser et al. 2009, Romero-Zarco 2011, Wölk and Röser 2013, 2017). These genera are part of Aveninae, except *Avenula* (*incertae sedis* in Poeae chloroplast group 2) and *Helictochloa* (Airinae, Poeae chloroplast group 2) (Soreng et al. 2015b). Two main clades have been identified within Aveninae. One clade comprises *Arrhenatherum*, *Avena*, *Helictotrichon* s.str., *Pseudarrhenatherum* and *Tricholemma* (Aveninae s.str.), commonly known as oat grasses (Quintanar et al. 2007, Schneider et al. 2009). The other clade comprises *Avellinia*, *Calamagrostis*/*Deyeuxia* p.p. *Gaudinia*, *Graphephorum*, *Koeleria*, *Lagurus*, *Leptophyllochloa*, *Peyritschia*, *Rostraria*, *Sphenopholis*, *Trisetaria*, *Trisetopsis*, ×*Trisetopsotrichon*, *Trisetum* and *Tzveleviochloa* (Quintanar et al. 2007, Saarela et al. 2010, Wölk and Röser 2013, 2014, 2017). Some authors separate the latter clade as subtribe Koeleriinae Asch. & Graebn. (Quintanar et al. 2007, 2010).

Agrostidinae, characterized by having single-flowered spikelets, includes ca. 16 genera and 600 species (Clayton and Renvoize 1986, Soreng et al. 2015b). Agrostidinae includes the diverse, ecologically important and taxonomically difficult genera *Agrostis* L. (ca. 220 species) and *Calamagrostis* Adans. s.l. (ca. 270 species), plus the following smaller genera: *Ammophila* Host (two species), *Ancistragrostis* S.T. Blake (one species), *Bromidium* Nees & Meyen (five species), *Chaetopogon* Janchen (one species), *Dichelachne* Endl. (five species), *Echinopogon* P. Beauv. (seven species), *Gastridium* P. Beauv. (two species), *Hypseochloa* C.E. Hubb. (two species), *Lachnagrostis* Trin. (ca. 20 species), *Limnodea* L.H. Dewey (one species), *Pentapogon* R. Br. (one species), *Podagrostis* (Griseb.) Scribn. & Merr. (ca. six species), *Polypogon* Desf. (18 species) and *Triplachne* Link (one species) (Soreng et al. 2015b). Kellogg (2015) also included *Cyathopus* Stapf in Agrostidinae, whereas Soreng et al. (2015b) included it in Poinae, consistent with molecular data (Hoffmann et al. 2013), and Kellogg (2015) included *Limnodea* in Poinae. Generic circumscriptions and evolutionary relationships among many taxa of Agrostidinae are poorly understood. For example, in a study based on sequences of the internal transcribed spacer (ITS) region of nrDNA and the plastid *trnL–trnF* region, Saarela et al. (2010) found considerable intermixing of multiple genera of Agrostidinae, and little backbone support in their trees.

Major unresolved taxonomic problems in Agrostidinae are the circumscriptions of *Calamagrostis* and *Deyeuxia* (hereafter *Calamagrostis*/*Deyeuxia*), which have been variously recognized globally as a single genus or separate genera (Clayton and Renvoize 1986, Clayton et al. 2006 onwards, Simon 2014, Soreng et al. 2015b). Saarela et al. (2010) made some progress towards resolving this issue by demonstrating polyphyly of *Calamagrostis*/*Deyeuxia*. Sampled species of *Calamagrostis*/*Deyeuxia* from north temperate regions resolved in Agrostidinae, whereas those from Mexico, Central and South America resolved in Koeleriinae. However, they sampled only one species of *Calamagrostis*/*Deyeuxia* from South America, where the genus is particularly diverse. Four South American species of *Calamagrostis*/*Deyeuxia* were included in a subsequent study focused on *Trisetopsis*, confirming their placement in Aveninae s.l. (Wölk and Röser 2014). Nevertheless, most South American taxa of *Calamagrostis*/*Deyeuxia* have not been included in molecular studies, and their affinities remain unresolved.

The objectives of this study are to clarify phylogenetic relationships in Poeae chloroplast group 1. We substantially increase taxonomic and genetic sampling of nrDNA and plastid regions across Poeae chloroplast group 1 compared to earlier studies. For example, our sampling includes 105 species of *Calamagrostis*/*Deyeuxia*. Although our focus is primarily on Poeae chloroplast group 1, we also include in our analyses a representative sampling of taxa of Poeae chloroplast group 2, given known intermixing of subtribes of Poeae chloroplast groups 1 and 2 and the lack of deep resolution in nrDNA trees. The ITS region, comprising internal transcribed spacers 1 (ITS 1) and 2 (ITS 2) and the intervening 5.8S gene, is part of the nrRNA cistron encoding the small ribosomal subunit (18S) and the large ribosomal subunits (5.8S and 26S) (Poczai and Hyvönen 2010). ITS is commonly sequenced in phylogenetic studies of grasses, but because ITS data alone do not resolve most deep branches in Poeae chloroplast group 1 (Saarela et al. 2010), we also sequenced the 3’-end of the external transcribed spacer region (ETS) of nrDNA, part of the same nrRNA cistron. The 3’-end of ETS is part of the intergenic spacer (IGS) between the repetitive 18S–5.8S–26S gene blocks including the ITS 1 and ITS 2 regions (Poczai and Hyvönen 2010). The ETS region has been used in phylogenetic studies in numerous angiosperm families (especially Asteraceae). In many cases ETS evolves faster than the ITS regions and is informative for phylogenetic reconstruction, especially when combined with ITS (Poczai and Hyvönen 2010). In Poaceae, the ETS region has been sampled in diverse grass genera (Duvall et al. 2003, Sallares and Brown 2004, Gillespie et al. 2009, Consaul et al. 2010, Catalán et al. 2012, Refulio-Rodriguez et al. 2012, Pimentel et al. 2013, Alonso et al. 2014, Birch et al. 2014, Scataglini et al. 2014, Soreng et al. 2015a), but of the subtribes of Poeae chloroplast group 1 its phylogenetic utility has only been characterized in Anthoxanthinae (Pimentel et al. 2013). Additionally, we present a densely sampled ITS phylogeny, including new and previously published ITS sequences of subtribe Poeae. Most of these sequences were generated as part of phylogenetic studies, but have not been analysed together in a single phylogenetic analysis. This builds on an earlier comprehensive ITS tree for Poeae chloroplast group 1 (Saarela et al. 2010) and provides a useful phylogenetic overview of the group, including a much broader sampling of taxa than is possible from matrices comprising more than one DNA region.

## Methods

### Taxon and genome sampling

The specimens included in this study were collected in the field by the authors and dried in silica-gel, or sampled from herbaria. Vouchers for specimens collected by the authors are deposited in the National Herbarium of Canada, Canadian Museum of Nature (CAN), the United States National Herbarium, Smithsonian Institution (US), and/or Herbarium of the Institute of Botany, Polish Academy of Sciences (KRAM). We aimed for broad taxonomic and geographic coverage of taxa in Poeae chloroplast group 1, and also sampled taxa representative of major lineages (subtribes) of Poeae chloroplast group 2, given known intermixing of subtribes of Poeae chloroplast groups 1 and 2 in nrDNA trees. We obtained new DNA sequence data from 421 individuals, with 1 to 17 (mean = 2.03 ± 1.88) individuals sampled per species. Following the classification of Soreng et al. (2015b), our sampling represents one subfamily (Pooideae), one tribe (Poeae), Poeae chloroplast group 1, Poeae chloroplast group 2 and 10 subtribes (Agrostidinae, Anthoxanthinae, Aveninae, Brizinae, Calothecinae, Holcinae, Loliinae, Phalaridinae, Poinae and Torreyochloinae). *Bromus vulgaris* (Hook.) Shear (tribe Bromeae) was designated as the outgroup given the close relationship between Bromeae and Poeae (Saarela et al. 2015). Voucher information and GenBank accession numbers for all new sequences are given in Appendix 1 and Suppl. material 1. Sources of previously published sequences are given in Suppl. material 2; in the figures these are appended with their GenBank accession number. Identifications of newly sequenced collections were made or confirmed by JMS, PMP, RJS and/or BP, and a large subset of the South American *Calamagrostis*/*Deyeuxia* material sampled from US had been identified or confirmed by Z.E. Rúgolo de Agrasar. Species of *Calamagrostis*/*Deyeuxia* from South America are referred to by their names in *Deyeuxia*, following Rúgolo de Agrasar (2006), except in the few cases where combinations in *Deyeuxia* are not available. Asian species of *Calamagrostis*/*Deyeuxia* are referred to by the generic names under which they were identified. The remaining north temperate species are referred to by their names in *Calamagrostis*, as commonly recognized.

### DNA sequencing and alignment

We extracted DNA from leaf material using a slightly modified version of the protocol outlined by Alexander et al. (2007). We sequenced the ITS and ETS regions of the nrDNA encoding ribosome subunits in eukaryotes. ITS includes the two internal transcribed spacer regions (ITS1 and ITS2) and the intervening 5.8S nrDNA locus. The following primers were used to amplify and sequence the ITS regions: ITS1, ITS2, ITS3, ITS4 (White et al. 1990); ITS_p2, ITS_p3, ITS_u2, ITS_u4 (Cheng et al. 2016); AB102, equivalent to 26SE from Sun et al. (1994); KRC (Torrecilla and Catalán 2002); and ITS5A (Stanford et al. 2000). The 3’-end of the ETS region was amplified and sequenced using primers RETS4-F (Gillespie et al. 2010) and 18S-R (Starr et al. 2003).

We sequenced five plastid regions, including (1) the ca. 841 bp central portion of the gene *matK* recommended for DNA barcoding; (2) the *trnL–trnF* region including a portion of the 5’-*trnL(UAA)* exon, the *3'-trnL(UAA)* exon, the *trnL(UAA)* intron, the *trnL(UAA)–trnF(GAA)* intergenic spacer and the 3’-*trnF(GAA)* gene; (3, 4) two intergenic spacer regions (*atpF–atpH*, *psbK–psbI*); and (5) the region spanning *trnH* to *psbA*. In grasses, the *rps19* gene is inserted between the *trnH* and *psbA* genes, so the widely sequenced “*psbA–trnH* intergenic spacer” comprises the *psbA–rps19* intergenic spacer, the *rps19* gene and the *rps19–trnH* intergenic spacer. For clarity, we refer to this region as *psbA–rps19–trnH*. *matK* was amplified and sequenced with matK-2.1F (Kress and Erickson 2007), matK-1326r (Cuénoud et al. 2002) and two new primers we designed: *matK*_po1F (5’-CGCTCTATTCATTCAATATTTC-3’) and *matK*_po3R (5’-CGTACCGTGCTTTTATGTTTACGAG-3’). *matK*_po1F has the same binding location as *matK*-390f (Cuénoud et al. 2002) but is modified by one nucleotide, and *matK*_po3R has the same binding location as MatK-3FKIM-r (Ki-Joong Kim, unpublished primer) but is modified by three nucleotides. For samples that would not amplify for the full *matK* fragment, internal primers matK_ag520F (5’-TGTTCGATATCAAGGAAAGGCA-3’) and matK_ag640R (5’-TCGCGGCTGAGTCCAAAAAG-3’) were designed to amplify and sequence the region in two overlapping fragments. The newly designed plastid primers were based on an alignment of Poeae chloroplast genomes (Saarela et al. 2015). *trnL–trnF* was amplified and sequenced with primers c, d, e and f developed by Taberlet et al. (1991) and five primers newly designed during this study: C_113f (5’-TCCTGAGCCAAATCCRTGTT-3’), F_1157rD (5’-AGCTATCCTGACCTTWTMTTRTG-3’), trnLF_181f (5’-AGGATAGGTGCAGAGACTCA-3’), trnLF_518f (5’- TGGATTAATCGGACGAGGACA-3’), and trnLF_808r (5’-TCTCTTCGCACTCCTTTGGG-3’). *atpF–atpH* and *psbK–psbI* were amplified and sequenced with the primers atpF, atpH, psbK and psbI (Fazekas et al. 2008). We also designed two new primers to amplify and sequence the *psbK–psbI* intergenic spacer: psbK_po1F (5’-TGGCAAGCTGCTGTAAGTTT-3’), psbI_po1R (5’-AAAGTTTGAGAGTAAGCAT-3’). *psbA–rps19–trnH* was amplified and sequenced with the primers psbAF (Sang et al. 1997) and trnH2 (Tate and Simpson 2003). Intron and exon boundaries for all plastid regions were determined by comparison with the complete plastid genome of *Agrostis stolonifera* L. (Saski et al. 2007).

PCR amplifications were performed in a 15 µl volume with 1X buffer, 1.5 mM of MgCl_2_, 0.2 mM dNTP, 0.5 µM of each primer, 0.3 U Phusion High-Fidelity DNA Polymerase, and 1 µL of DNA template. The thermal profile was initial denaturing of 30 sec at 98 °C; 34 cycles of 10 sec at 98 °C, 30 sec at 56 °C, and 30 sec at 72 °C; and a final extension of 5 min at 72 °C. Sequencing products were generated using BigDye Terminator v3.1 Cycle Sequencing Kits (ThermoFisher Scientific, Waltham, MA, U.S.A.) with 0.5 µl of BigDye Ready Reaction Mix in a 10 µl reaction with 1 µL of PCR product as template, and the following thermal profile: initial denaturing of 3 min at 95 °C, 30 cycles of 30 sec at 96 °C, 20 sec at 50 °C, and 4 min at 60 °C. Sequencing reactions were analyzed via capillary electrophoresis using an Applied Biosystems 3130*xl* Genetic Analyzer. We performed base-calling and contig assembly using Sequencher 4.7 (Genes Code Corporation, Ann Arbor, Michigan) and Geneious version 8.1.8 (http://www.geneious.com) (Kearse et al. 2012). Sites in nrDNA sequences with polymorphic bases were scored as N. Alignments were generated using MUSCLE (Edgar 2004) and other alignment tools in Geneious, and then edited manually.

We compiled individual matrices for each of the seven DNA regions studied. New sequences were validated (quality control) throughout the data collection phase. A large proportion of the variable characters in the alignments, particularly those near the beginnings and ends of contigs and when we observed infraspecific variation (i.e., when multiple individuals of a species were sampled), were carefully checked on chromatograms and edited as necessary to ensure accuracy in base calling. This process was conducted iteratively for each matrix as new sequences were added. To check for putative contamination, misidentification and/or other errors, we generated neighbour joining trees for each of the seven separate matrices using the PAUP* plugin in Geneious. These trees were examined for individuals that clustered in different parts of the trees compared to congeneric and/or conspecific taxa. We re-examined the voucher specimens for these problematic samples and corrected misidentified specimens as necessary. Some previously published sequences were grossly misplaced in the ITS tree in preliminary analyses. We concluded these are erroneous (data not shown), probably reflecting mis-identifications or laboratory mix ups, and excluded them from subsequent analyses. Once the matrices were finalized, we concatenated the two nrDNA and five plastid regions into single matrices (Suppl. materials 12–19). A summary of the seven matrices is presented in Table 1, including the number of new and previously published sequences in each matrix, the length of each aligned matrix, and the average, minimum and maximum lengths of sequences in each matrix.

In this study, we generated 2425 new sequences, and the number of new sequences per DNA region ranges from 294 (ITS) to 379 (*psbA–rps19–trnH*) (Table 1). The ITS matrix is the largest and includes 1079 accessions. The ETS matrix includes 352 sequences, of which 328 are new. The combined ITS+ETS matrix includes 338 samples, with both regions complete for each. The *psbA–rps19–trnH* (380 sequences), *atpF–atpH* (356) and *psbK–psbI* (391) matrices comprise new data for all accessions except *Bromus vulgaris*. The *matK* matrix includes 928 sequences, of which 367 are new. The combined plastid matrix includes 383 accessions, each with data for at least three of the five plastid regions.

**Table 1.** Summary statistics for nuclear ribosomal and plastid sequence data.

DNA region	No. of sequences in matrix	No. of new sequences in matrix	No. of published sequences in matrix	Alignment length	Unaligned sequence length (x‒ ± s.d.) (bp)	Maximum sequence length (bp)	Minimum sequence length (bp)
ITS [3’-18S–ITS1–5.8S–ITS2–5’-26S]	1079	294	785	1137	687 ± 154	1008	205
ITS 1				272	211 ± 15	221	16
5.8S				165	16 ± 22	165	1
ITS 2				266	209 ± 18	219	27
ETS	352	328	24	1925	548 ± 57	864	265
*atpF–atpH*	356	355	1	739^1^	599 ± 79	673	309
*matK*	928	367	561	1555	920 ± 295	1542	400
*matK* (reduced)^*^	368	367	1	966	774 ± 98	957	461
*psbA–rps19–trnH*	380	379	1	759^2^	583 ± 67	680	346
*psbK–psbI*	392	391	1	586^3^	362 ± 107	471	150
*trnL–trnF*	474	311	163	1481^4^	785 ± 118	1026	310
*trnL–trnF* (reduced)^**^	341	311	30	1418	816 ± 78	1026	498

^1^ The aligned *atpF–atpH* intergenic spacer is 521 bp; the entire region includes flanking portions of the *atpF* and *atpH* genes.

^2^ The *psbA–trnH* intergenic spacer is 142 bp, the *rsps19* gene is 219 bp, and the *rps19–trnH* intergenic spacer is 206 bp; the entire region includes flanking portions of the *psbA* and *trnH* genes.

^3^ The aligned *psbK–psbI* intergenic spacer is 519 bp; the entire region includes flanking portions of the *psbK* and *psbI* genes.

^4^ The aligned *trnL* intron is 671 bp, the aligned *3'-trnL* exon is 65 bp and the aligned *trnL–trnF* intergenic spacer is 668 bp; the entire region includes flanking portions of the *5'-trnL* exon and the *trnF* gene.

^*^ including only newly generated data and the outgroup

^**^ including newly generated data and a small subset of previously published data, including the outgroup

Two plastid matrices included small inversions, identified as the reverse complements of other individuals’ nucleotides in the same alignment positions and flanked by inverted repeats, similar to what has been found elsewhere (Kelchner and Wendel 1996, Graham et al. 2000). We replaced these inversions with their reverse complement sequences, as recommended by Whitlock et al. (2010). Inversions and the taxa in which they were observed are described in Appendix 1 and Suppl. material 1. A two base pair inversion in the *rps19–trnH* intergenic spacer region flanked by a six bp inverted repeat could not unambiguously be modified to the same inversion configuration because of a high level of intra and interspecific variation in the two bp; 9 of 12 possible permutations were present in the matrix (i.e., GG CC CG CT TG GA AG TC CA). We staggered this alignment region because it was impossible to simultaneously determine positional and inversion configuration homology.

### Phylogenetic analyses

All analyses were conducted on the CIPRES science Gateway (Miller et al. 2010). We conducted maximum likelihood (ML) and Bayesian inference (BI) analyses with the ITS+ETS and combined plastid matrices, and ML analyses with each individual plastid matrix and the ITS matrix, which was too large for BI analysis to reach convergence in the maximum available analysis time (168 hrs) on the CIPRES server (J.M. Saarela, pers. obs.). We determined models of evolution using the Akaike Information Criterion (AIC) in jModelTest2 (Darriba et al. 2012). The best fit models for the data partitions were General Time Reversible (GTR) incorporating a gamma distribution (GTR + G) for the ETS and the ITS+ETS matrices, and TVM + G for the combined plastid matrix. In all analyses, gaps were treated as missing data; we did not code indels as separate characters. We conducted BI analyses in MrBayes 3.2.6 (Ronquist et al. 2012), with default prior settings. For the plastid matrix, we used GTR+G, the model closest to TVM + G in MrBayes. The Markov chain Monte Carlo (MCMC) analysis was set to run for 2 × 50,000,000 generations with four chains and sampled every 1000 chains, and the analysis was set to stop early if the convergence diagnostic (average standard deviation of split frequencies) was less than 0.01. The combined plastid analysis reached convergence after 8,460,000 generations. ML analyses were performed using RAxML-HPC Black Box (Stamatakis 2014), with the GTR+G model (the only one available), and bootstrapping was automatically halted based on default criteria. Trees were visualized in FigTree v.1.4.2 (http://tree.bio.ed.ac.uk/software/figtree/).

We present phylograms of the ML trees in the main text, and report both ML bootstrap and BI posterior probabilities on the ITS+ETS and combined plastid ML trees. For each of the three analyses, we provide a summary tree in which major clades, often corresponding to subtribes, are collapsed to clearly show relationships among major lineages. We present the details of these trees in multiple figures, and on each summary tree note the subsequent figures in which detailed results of the tree are presented. The ITS+ETS tree is divided into six figures, the ITS tree into nine figures, and the plastid tree into six figures. A subset of the ITS tree (Airinae p.p., Holcinae p.p., Poinae, Miliinae and Coleanthinae) is not presented in the main text. All trees are provided in full in Suppl. materials 3–11, including all single-region ones. We use the terms ‘weak or poor’, ‘moderate’, and ‘strong’ in reference to clades that received bootstrap support values of <70%, 70–90% and 91–100%, respectively; and posterior probabilities <.8, .8–.94 and .95–1, respectively. We use the term ‘unsupported’ for clades with bootstrap support <50%.

## Results

### ITS+ETS and ITS analyses

Several clades corresponding to subtribes, subtribes in part and/or multiple subtribes are recovered with moderate to strong support in the ITS+ETS tree (Figs 1, 4–9, Suppl. material 3). The ITS tree (Figs 2, 10–18, Suppl. material 4) includes substantially greater taxon sampling than the ITS+ETS tree and the ETS tree (Suppl. material 5), and identifies many of the clades recovered in the ITS+ETS tree, but support across the tree is mostly weaker, especially for deeper branches.

**Figure 1.**
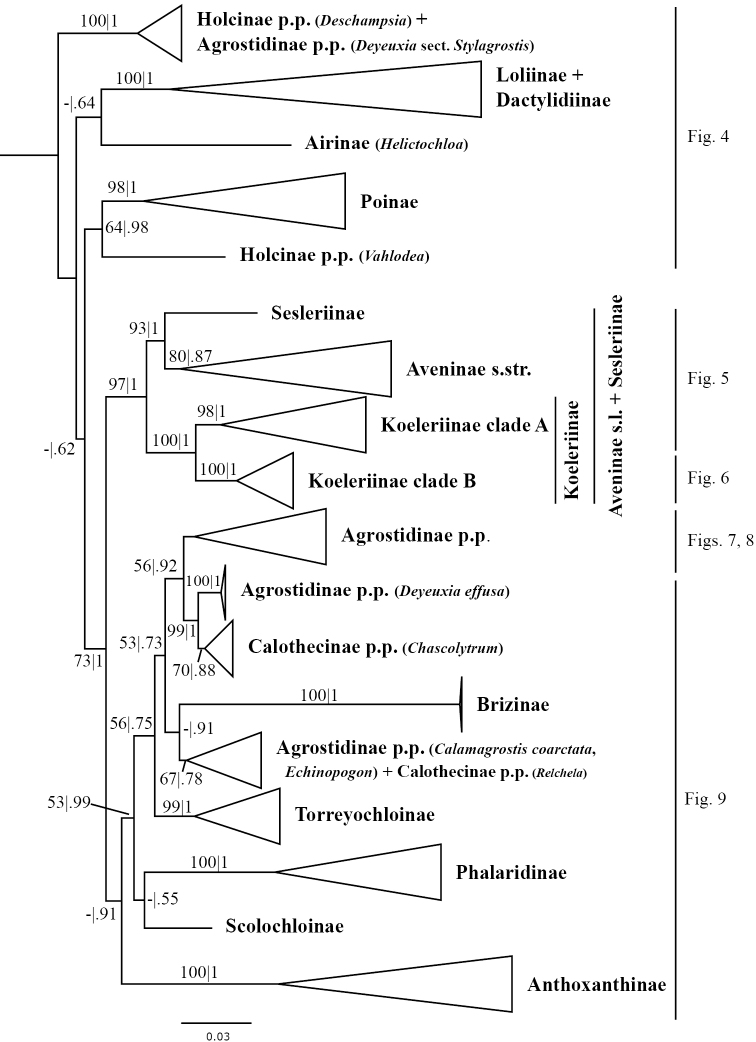
Overview of the maximum likelihood phylogram inferred from ITS+ETS data. Major clades in the complete tree are collapsed. The corresponding figures showing details of subsections of the tree are indicated. ML bootstrap support (left) and BI poster probabilities (right) are recorded along branches. A dash indicates bootstrap support <50%. No support is shown for branches with bootstrap support <50% and posterior probability <.5. The ML tree is presented in its entirety in Suppl. material 3.

**Figure 2.**
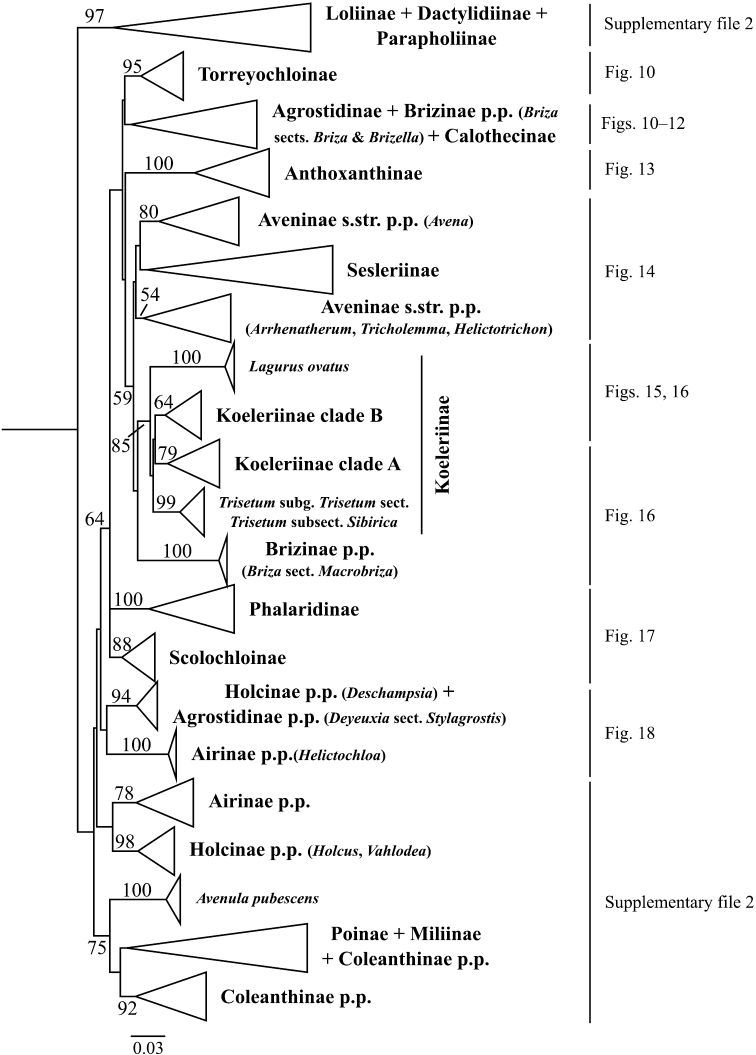
Overview of the maximum likelihood phylogram inferred from ITS data. Major clades in the complete tree are collapsed. The corresponding figures showing details of subsections of the tree are indicated. ML bootstrap support is recorded along branches. No support is shown for branches with bootstrap support <50%. The ML tree is presented in its entirety in Suppl. material 4.

Aveninae s.str. is monophyletic in the ITS+ETS tree because *Arrhenatherum*, *Avena* and *Helictotrichon* form a moderately supported clade (bootstrap support = 80%, posterior probability = .87; Figs 1, 5). In the ITS tree, however, Aveninae s.str. is not monophyletic because the four sampled genera form two separate clades: a moderately supported clade comprises *Avena* (80; Figs 2, 14) and a weakly supported clade comprises *Arrhenatherum*, *Helictotrichon* s.str. and *Tricholemma* (54; Figs 2, 14). All species of *Arrhenatherum* form a maximally supported clade (Fig. 14), and *Arrhenatherum* and *Tricholemma* are weakly supported as sister taxa (68; Fig. 14). All species of *Helictotrichon* s.str. form a moderately supported clade (76; Fig. 14). Also in the ITS tree, an unsupported lineage corresponds to Sesleriinae (Figs 2, 14) and is divided into two moderately to strongly supported subclades, one comprising *Oreochloa* Link and *Mibora* Adans. (79), the other *Echinaria* Link and *Sesleria* (98) (Fig. 14).

A clade corresponding to Koeleriinae is strongly supported in the ITS+ETS tree (100, 1; Figs 1, 5), and this clade is divided into two strongly supported subclades referred to here as Koeleriinae clade A (98, 1; Figs 1, 5) and Koeleriinae clade B (100, 1; Figs 1, 6). A clade corresponding to Koeleriinae is moderately supported in the ITS tree (85; Figs 2, 15, 16) and is divided into four lineages, including Koeleriinae clade A (79; Figs 2, 16) and Koeleriinae clade B (64; Figs 15, 16), both with weaker support than in the ITS+ETS tree. The two additional lineages comprise taxa not sampled in the ITS+ETS tree: *Lagurus ovatus* L., on a relatively long branch (Figs 2, 15), and a strongly supported clade corresponding to Trisetum subsect. Sibirica (Chrtek) Prob. (99; Figs 2, 16). Relationships among these four lineages of Koeleriinae in the ITS tree are unsupported.

Our analyses identify several lineages within Koeleriinae clade A in the ITS+ETS tree. One clade is strongly supported and comprises *Trisetum cernuum* Trin. (Trisetum sect. Trisetum) and *Graphephorum wolfii* J.M. Coult. (95, 1; Fig. 5), with *T. distichophyllum* P. Beauv. resolved as its sister group (90, .7; Fig. 5). This three taxon clade is sister to a large, weakly supported clade (59, .77; Fig. 5) including the following successively diverging lineages: (1) a maximally supported clade of *T. flavescens* (L.) P. Beauv. (Trisetum sect. Trisetum) and *Rostraria pumila* (Desf.) Tzvelev; (2) *Avellinia michelii* (Savi) Parl.; (3) *Gaudinia fragilis* (L.) P. Beauv.; and (4) a clade including species of *Koeleria*, Trisetum sect. Trisetaera Asch. & Graebn. and Trisetum sect. Trisetum p.p. (*T. macbridei* Hitchc., *T. irazuense* (Kuntze) Hitchc.). In the better-sampled ITS tree, Koeleriinae clade A similarly includes *Avellinia michelii*, *Gaudinia fragilis*, *Graphephorum*, *Rostraria*, *Trisetaria* and *Trisetum* sects. *Trisetum* and *Trisetaera*. However, most relationship among taxa in the clade are unsupported. Strongly supported lineages within Koeleriinae clade A in the ITS tree include clades of (1) *Trisetaria dufourei* (Boiss.) Paunero and *T. loeflingiana* (L.) Paunero (97; Fig. 16), and (2) *Rostraria* p.p. (three species) and Trisetum sect. Trisetum p.p. (three species, including *T. flavescens*) (99; Fig. 16). The latter clade corresponds to a more poorly sampled clade in the ITS+ETS tree.

Our analyses identify several lineages within Koeleriinae clade B in the ITS+ETS tree. Koeleriinae clade B is divided into three deep lineages that form a trichotomy (Fig. 6). One large, weakly to strongly supported clade includes Trisetum subg. Deschampsioidea (Louis-Marie) Finot, *Calamagrostis*/*Deyeuxia* p.p. (species from Mexico and South America) and *Leptophyllochloa* (56, 1; Fig. 6). Most of the species from Mexico (*Calamagrostis*, Trisetum subg. Deschampsioidea) form a clade. The four sampled species of *Sphenopholis* form a strongly supported clade (92, 1; Fig. 6). A weakly supported clade includes *Peyritschia deyeuxioides* (Kunth) Finot and five species of *Calamagrostis*/*Deyeuxia* from South America (53, -; Fig. 6). The ITS tree includes the same taxa as well as *Trisetopsis* (not sampled in the ITS+ETS tree), but relationships in the clade are more poorly resolved and supported (Fig. 15) than in the ITS+ETS tree. In the ITS tree, one clade of multiple species of *Calamagrostis*/*Deyeuxia* from South America is weakly supported (52; Fig. 15), the two sampled species of *Peyritschia* form a clade (63; Fig. 15), and the species of *Trisetopsis* form an unsupported clade (Fig. 15).

**Figure 3.**
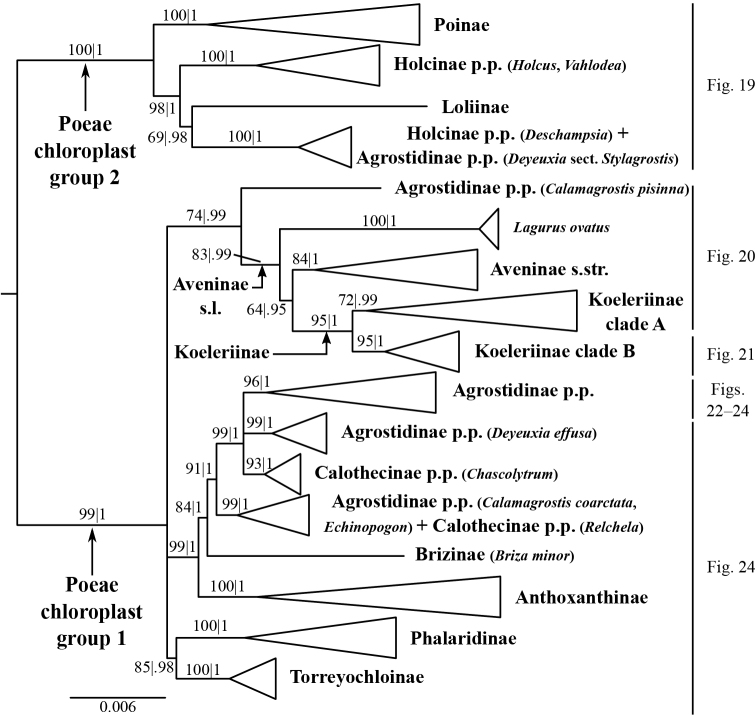
Overview of the maximum likelihood phylogram inferred from combined plastid data (*atpF–atpH*, *psbK–psbI*, *psbA–rps19–trnH*, *matK*, *trnL–trnF*). Major clades in the complete tree are collapsed. The corresponding figures showing details of subsections of the tree are indicated. ML bootstrap support (left) and BI poster probabilities (right) are recorded along branches. No support is shown for branches with bootstrap support <50% and posterior probability <.5. The ML tree is presented in its entirety in Suppl. material 6.

A large clade comprising taxa of Agrostidinae, Brizinae and Calothecinae is weakly supported in the ITS+ETS tree (53, .73; Figs 1, 7–9) and unsupported in the ITS tree (Figs 2, 10–12), and none of these subtribes are monophyletic in the nrDNA trees. This clade includes four main lineages in the ITS+ETS tree: (1) Agrostidinae p.p. (unsupported), including most taxa currently classified in the subtribe; (2) a strongly supported clade of *Deyeuxia effusa* (Agrostidinae p.p.) and *Chascolytrum* (Calothecinae p.p.) (99, 1; Figs 1, 9); (3) Brizinae; (4) a weakly supported clade of *Calamagrostis coarctata* Eaton, *Echinopogon caespitosus* C.E. Hubb. (both Agrostidinae p.p.) and *Relchela panicoides* Steud. (Calothecinae p.p.) (67, .78; Figs 1, 9). These same clades are not resolved in the ITS tree. Calothecinae is not monophyletic in the nrDNA trees because *Chascolytrum* and *Relchela* do not form a clade. *Chascolytrum*, however, is monophyletic: the two sampled species (*C. subaristatum* Desv. and *C. monandrum* (Hack.) Essi, Longhi-Wagner & Souza-Chies) form a moderately supported clade (70, .88; Fig. 9) in the ITS+ETS tree, and the multiple sampled species form an unsupported clade in the ITS tree (Fig. 12). Neither Brizinae nor *Briza* are monophyletic in the ITS tree, because *Briza* and *Airopsis* do not form a clade and two lineages of *Briza* are resolved. One lineage of *Briza* is represented by *B. maxima* L., which is part of weakly supported clade including Aveninae s.str., Koeleriinae and Sesleriinae (Figs 2, 16). The other lineage includes the four other species of *Briza* sampled, which form a strongly supported clade of unclear relationship relative to taxa of Agrostidinae and Calothecinae (99; Figs 2, 10). The relationship of *Airopsis* to *Briza* and taxa of Agrostidinae and Calothecinae is similarly unclear. We were not able to test the monophyly of Brizinae or *Briza* in the ITS+ETS analyses because only one species of *Briza* is sampled there.

Agrostidinae is not monophyletic in the nrDNA trees given the placements of *Calamagrostis coarctata*, *Echinopogon* and *Deyeuxia effusa* in the broader Agrostidinae + Brizinae + Calothecinae clade, and some species of *Calamagrostis*/*Deyeuxia* in a clade with *Deschampsia* P. Beauv. (see below). Moreover, even though most other genera and species traditionally recognized in Agrostidinae and sampled here are part of the Agrostidinae + Brizinae + Calothecinae clade, they do not resolve in a supported clade in the nrDNA trees (Figs 1, 2). However, the broader clade comprising Agrostidinae p.p., *Deyeuxia effusa* and *Chascolytrum* is weakly supported in the ITS+ETS tree (Figs 1, 8). There is also some clear phylogenetic structure among subsets of taxa of Agrostidinae p.p. Species of *Agrostis*, *Lachnagrostis* and *Polypogon* are intermixed in two strongly supported clades in the nrDNA trees (Figs 7, 11, 12). In the ITS+ETS tree, a maximally supported clade comprises all species of *Agrostis* except *A. exarata* Trin., and *P. elongatus* Kunth (Polypogon sect. Polypogonagrostis Asch. & Graeb.) (Fig. 7). A similar strongly supported clade is present in the ITS tree, and also includes three species of *Lachnagrostis* not sampled in the ITS+ETS tree and *Chaetopogon fasciculatus* (Link) Hayek. (also not sampled in the ITS+ETS tree) (94; Fig. 12). The other clade in the ITS+ETS tree is maximally supported and comprises the four sampled species of Polypogon sect. Polypogon, *A. exarata* and *L. adamsonii* (Fig. 7). The equivalent clade in the ITS tree is strongly supported and includes Polypogon sect. Polypogon, *A. exarata* and three species of *Lachnagrostis*, of which only *L. adamsonii* is sampled in the ITS+ETS tree (97, Fig. 11).

**Figure 4.**
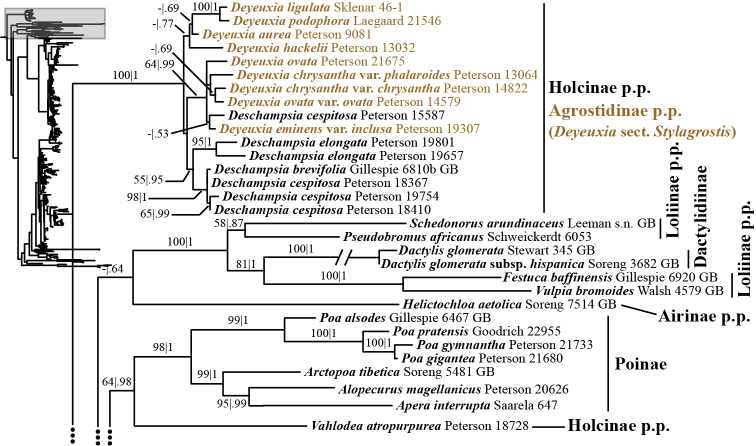
A portion (Holcinae p.p., Agrostidinae p.p., Loliinae, Dactylidinae and Poinae) of the maximum likelihood phylogram inferred from ITS+ETS data. ML bootstrap support (left) and BI poster probabilities (right) are recorded along branches. A dash indicates bootstrap support <50%. No support is shown for branches with bootstrap support <50% and posterior probability <.5. The shaded area of the smaller tree on the left indicates the location in the overall tree of the portion shown. The branch subtending Dactylidinae, with double slashes, is shortened for presentation. Backbone branches represented by ellipses are shown only in Figure 1.

**Figure 5.**
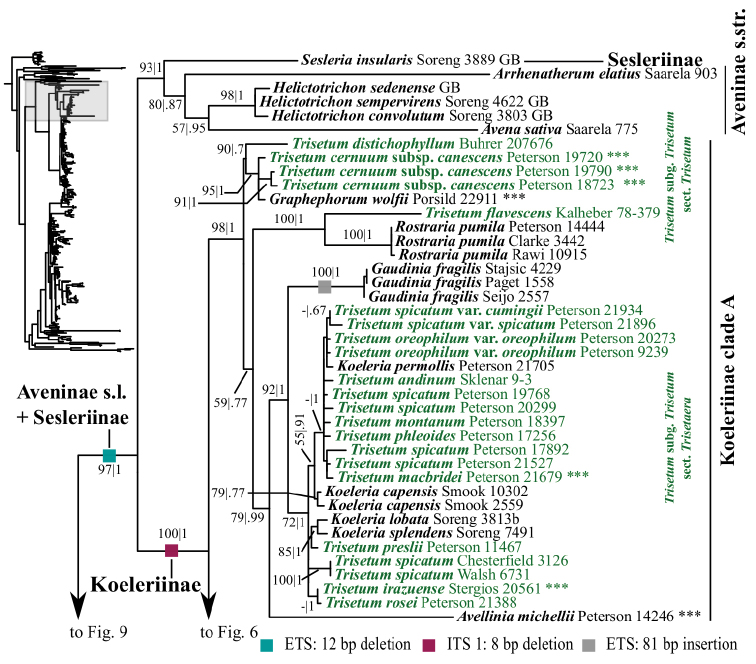
A portion (Sesleriinae, Aveninae s.str., Koeleriinae clade A) of the maximum likelihood phylogram inferred from ITS+ETS data. ML bootstrap support (left) and BI poster probabilities (right) are recorded along branches. A dash indicates bootstrap support <50%. No support is shown for branches with bootstrap support <50% and posterior probability <.5. The shaded area of the smaller tree on the left indicates the location in the overall tree of the portion shown. Placements of samples with asterisks (***) are incongruent in nrDNA and plastid trees. Two indels in ETS and one in ITS are mapped onto the phylogram.

**Figure 6.**
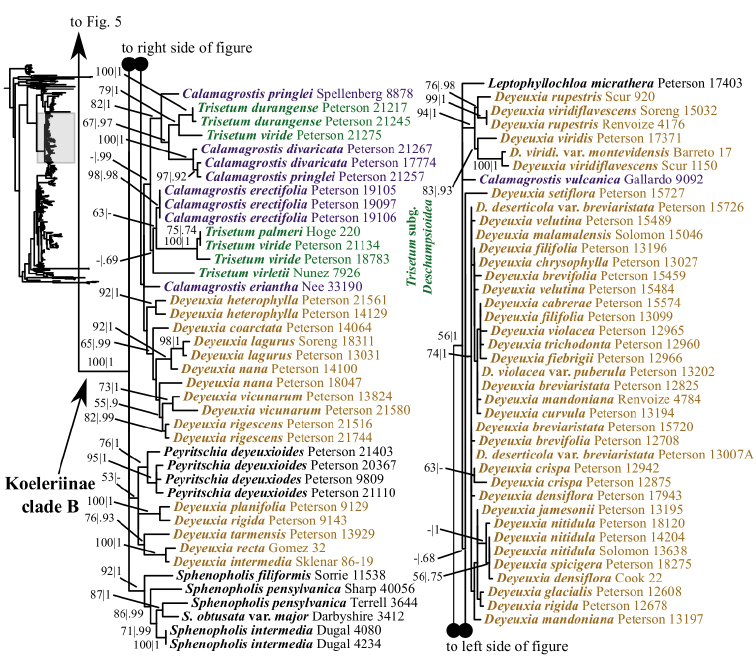
A portion (Koeleriinae clade B) of the maximum likelihood phylogram inferred from ITS+ETS data. ML bootstrap support (left) and BI poster probabilities (right) are recorded along branches. A dash indicates bootstrap support <50% or posterior probability <.5. No support is shown for branches with bootstrap support <50% and posterior probability <.5. The shaded area of the smaller tree on the left indicates the location in the overall tree of the portion shown.

**Figure 7.**
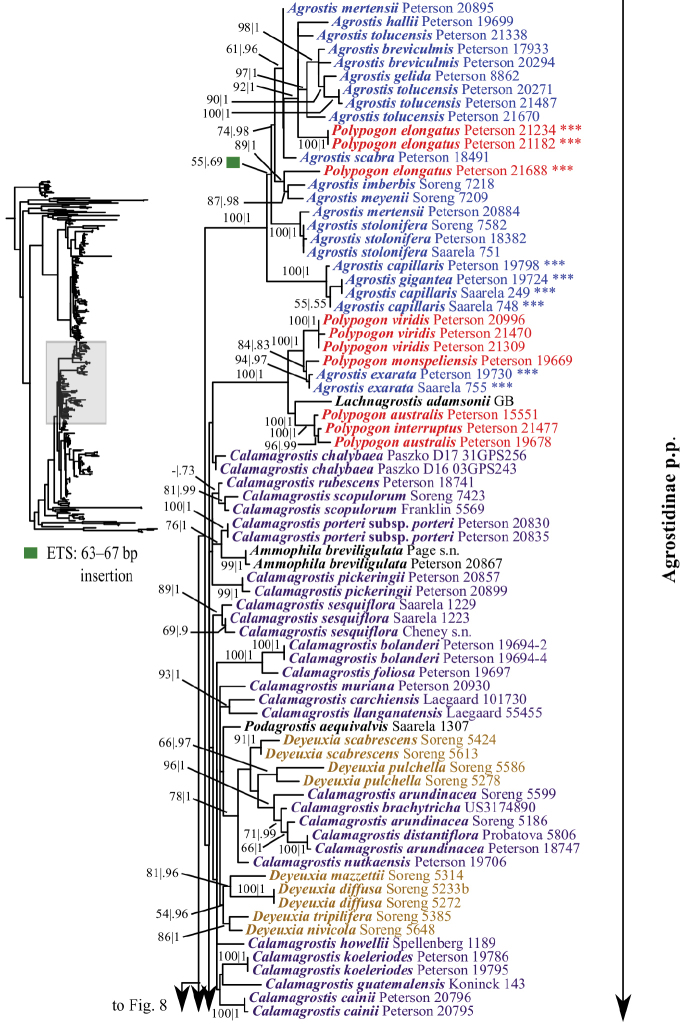
A portion (part of Agrostidinae p.p.) of the maximum likelihood phylogram inferred from ITS+ETS data. ML bootstrap support (left) and BI poster probabilities (right) are recorded along branches. A dash indicates bootstrap support <50%. No support is shown for branches with bootstrap support <50% and posterior probability <.5. The shaded area of the smaller tree on the left indicates the location in the overall tree of the portion shown. Placements of samples with asterisks (***) are incongruent in nrDNA and plastid trees. One indel in ETS is mapped onto the phylogram.

**Figure 8.**
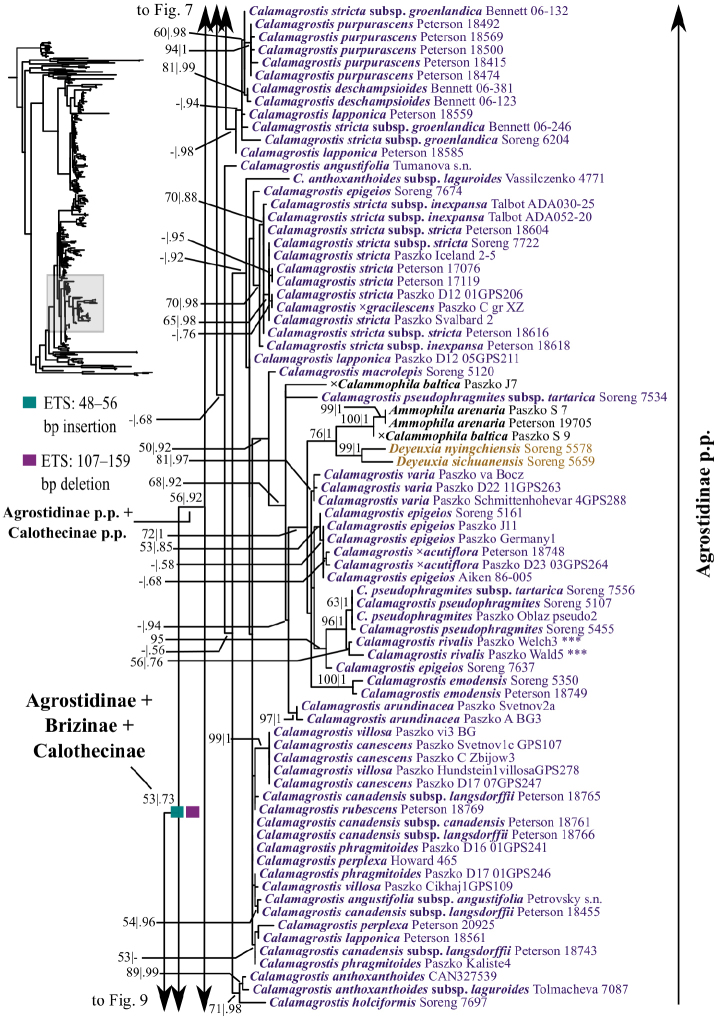
A portion (part of Agrostidinae p.p.) of the maximum likelihood phylogram inferred from ITS+ETS data. ML bootstrap support (left) and BI poster probabilities (right) are recorded along branches. A dash indicates bootstrap support <50%. No support is shown for branches with bootstrap support <50% and posterior probability <.5. The shaded area of the smaller tree on the upper left indicates the location in the overall tree of the portion shown. Placements of samples with asterisks (***) are incongruent in nrDNA and plastid trees. Two indels in ETS are mapped onto the phylogram.

The species of *Calamagrostis*/*Deyeuxia* that are part of the Agrostidinae + Brizinae + Calothecinae, excluding the more distantly related *Calamagrostis coarctata* and *Deyeuxia effusa*, do not form a clade in the nrDNA trees. However, some smaller clades of *Calamagrostis*/*Deyeuxia* are resolved. Moreover, the two species of *Ammophila* are included in different clades with species of *Calamagrostis*/*Deyeuxia*: *Ammophila* is not monophyletic. *Ammophila breviligulata* Fernald and *Calamagrostis porteri* A. Gray form a clade in the ITS+ETS (76, 1; Fig. 7) and ITS trees (64, Fig. 11). The broader affinities of this two-taxon clade are unsupported in the ITS+ETS tree, whereas the clade is part of a broader weakly supported clade in the ITS tree also including *C. pickeringii* A. Gray, *C. perplexa* Scribn. and *C. cainii* Hitchc. (75; Fig. 11). *Ammophila arenaria* (L.) Link, one accession of ×*Calammophila baltica* (Flüggé ex Schrad.) Brand. and two Chinese species of *Deyeuxia* (*D. nyingchiensis* P.C. Kuo & S.L. Lu and *D. sichuanensis* (J.L. Yang) S.M. Phillips & W.L. Chen.) form a clade in the ITS+ETS tree (76, 1; Fig. 8), and these three taxa are part of a broader clade including a second accession of ×*Calammophila baltica*, *C. arundinacea* (L.) Roth p.p., C. × acutiflora (Schrad.) DC., *C. emodensis* Griseb., *C. epigeios* (L.) Roth p.p., *C. pseudophragmites* (Haller f.) Koeler, *C. rivalis* H. Scholz and *C. varia* (Schrad.) Host. (72, 1; Fig. 8). *Ammophila arenaria* and the same two Chinese species of *Deyeuxia*, along with *C. coarctata*, form an unsupported clade in the ITS tree that is part of a broader unsupported clade including *Echinopogon* and *Relchela* (Fig. 10). Other clades with two or more taxa of *Calamagrostis*/*Deyeuxia* in the ITS+ETS tree comprise (1) *C. bolanderi* Thurb. and *C. foliosa* Kearney (100, 1; Fig. 7); (2) *C. nutkaensis* (J. Presl) J. Presl ex Steud., *C. arundinacea* p.p., *C. brachytricha* Steud., *C. distantiflora* Luchnik, *D. scabrescens* (Griseb.) Munro ex Duthie and *D. pulchella* (Griseb.) Hook. f. (78, 1; Fig. 7); (3) *D. diffusa* Keng, *D. mazzettii* Veldkamp, *D. tripilifera* Hook. f. and *D. nivicola* Hook. f. (54, .96; Fig. 7); (4) C. stricta subsp. groenlandica (Schrank) Á. Löve p.p., *C. purpurascens* R. Br. and *C. deschampsioides* Trin. (60, .98; Fig. 8); (5) *C. epigeios* p.p., *C. stricta* (Timm) Koeler p.p., C. × gracilescens Blytt (70, .98; Fig. 8); (6) *C. canescens* (Weber ex F.H. Wigg.) Roth and *C. villosa* (Chaix) J.F. Gmelin (99, 1; Fig. 8); (7) *C. anthoxanthoides* Regel p.p. and *C. holciformis* Jaub. & Spach (71, .98; Fig. 8). Some similar clades are resolved in the ITS tree, but most aspects of relationship among species of *Calamagrostis*/*Deyeuxia* in the ITS tree are poorly supported (Figs 10–12). The ITS tree also includes four accessions of *Dichelachne*, which form a clade, and these species are allied with four species of *Deyeuxia* from Australia and New Zealand (Fig. 12). *Gastridium* and *Triplachne* are maximally supported as sister taxa (Fig. 11) in the ITS tree, but their broader affinities are unresolved; these genera are not sampled in the ITS+ETS tree.

**Figure 9.**
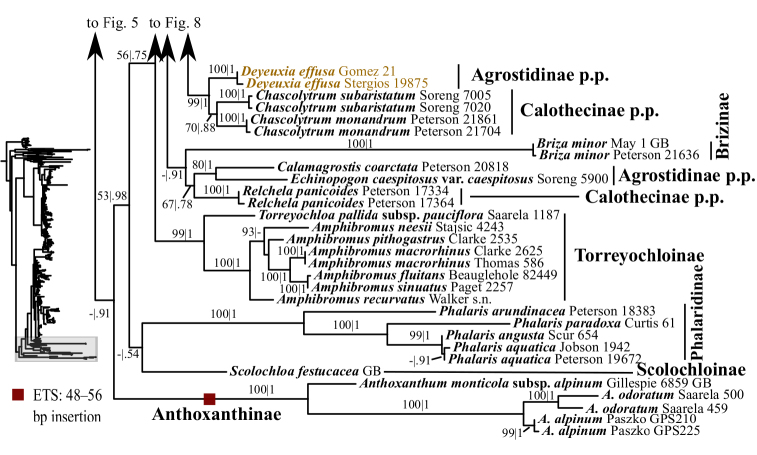
A portion (part of Agrostidinae p.p., Anthoxanthinae, Brizinae, Calothecinae, Phalaridinae, Scolochloinae and Torreyochloinae) of the maximum likelihood phylogram inferred from ITS+ETS data. ML bootstrap support (left) and BI poster probabilities (right) are recorded along branches. A dash indicates bootstrap support <50%. No support is shown for branches with bootstrap support <50% and posterior probability <.5. The shaded area of the smaller tree on the left indicates the location in the overall tree of the portion shown. One indel in ETS is mapped onto the phylogram.

**Figure 10.**
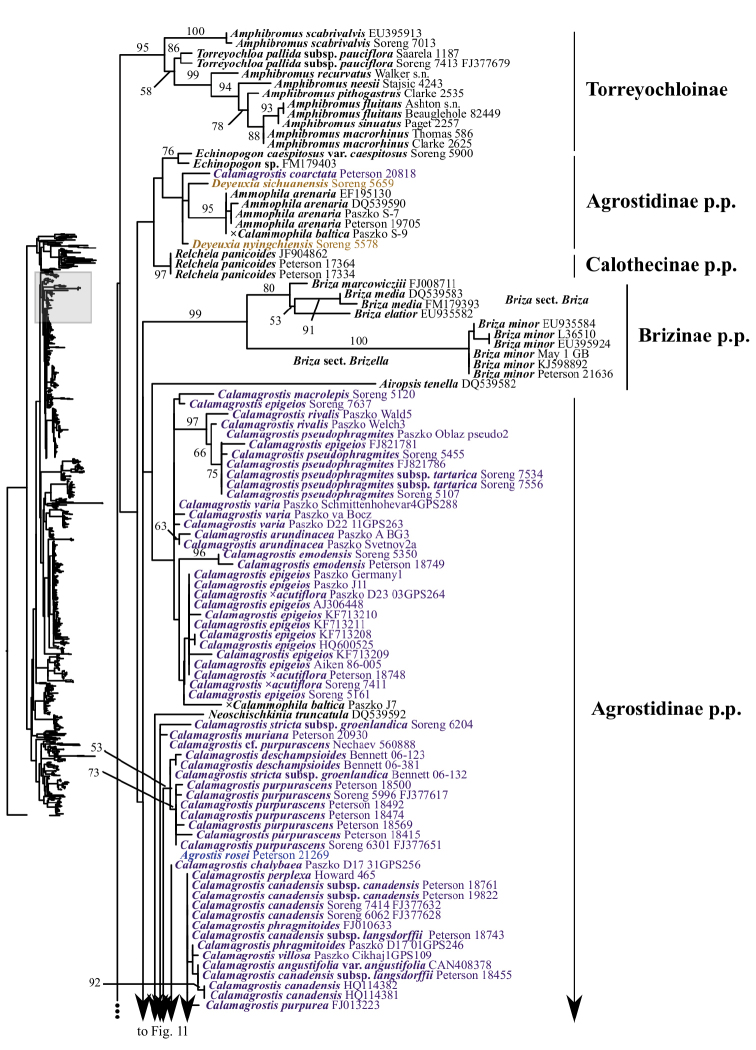
A portion (part of Agrostidinae p.p., Brizinae p.p., Calothecinae p.p. and Torreyochloinae) of the maximum likelihood phylogram inferred from ITS data. ML bootstrap support is recorded along branches when >50%. The shaded area of the smaller tree on the left indicates the location in the overall tree of the portion shown. Backbone branches represented by ellipses are shown only in Figure 2.

The other sampled tribes of Poeae chloroplast group 1 include Torreyochloinae, Phalaridinae, Scolochloinae and Anthoxanthinae. Torreyochloinae is monophyletic and strongly supported in the ITS+ETS (99, 1; Figs 1, 9) and ITS (95; Figs 2, 10) trees. In the ITS+ETS tree, Torreyochloinae and the Agrostidinae + Brizinae + Calothecinae clade are weakly supported as sister groups (56, .75; Figs 1, 9). Within Torreyochloinae, *Torreyochloa pallida* (Torr.) G.L. Church is sister to a maximally supported *Amphibromus* clade in the ITS+ETS tree, (Fig. 9), whereas *A. scabrivalvis* Swallen (not sampled in the ITS+ETS tree) is sister to a weakly supported clade comprising *T. pallida* and the remainder of *Amphibromus* (Fig. 10) in the ITS tree. *Amphibromus* is not monophyletic in this tree. Phalaridinae (*Phalaris*) is maximally supported in the ITS+ETS (Fig. 1, 9) and ITS trees (Figs 2, 17). In the ITS tree, *Phalaris* sects. *Digraphis* Link and *Caroliniana* Voshell, Stephanie M., Baldini & Hilu are sister groups (63; Fig. 17), and relationships among this clade, *Phalaris* sects. *Bulbophalaris* Tzvelev + *Heterachne* Dumort. (intermixed in a clade) and Phalaris sect. Phalaris are unresolved (Fig. 17). In the ITS+ETS tree, Phalaridinae and Scolochloinae (*Scolochloa*) are part of a broader weakly supported clade with Torreyochloinae, Agrostidinae, Calothecinae and Brizinae. The two genera of Scolochloinae (*Dryopoa* Vickery and *Scolochloa* Link) are sampled in the ITS tree, and the subtribe is monophyletic (88; Fig. 17). Anthoxanthinae (*Anthoxanthum*) is maximally supported in the ITS+ETS (Figs 1, 9) and ITS trees (Figs 2, 13). Clades corresponding to *Anthoxanthum* sects. *Anthoxanthum* and *Ataxia* (R. Br.) Stapf are maximally supported in the ITS tree, and two other clades comprising the rest of the sampled species are resolved.

In addition to Sesleriinae and Scolochloinae, which are classified in Poeae chloroplast group 2 but closely related to taxa of Poeae chloroplast group 1 in nrDNA trees, we newly sampled exemplars representing five other subtribes of Poeae chloroplast 2: Airinae, Holcinae, Dactylidinae, Loliinae and Poinae. Unexpectedly, a subset of species of *Calamagrostis*/*Deyeuxia* from South America recognized in Deyeuxia sect. Stylagrostis (Mez) Rúgolo & Villav. form a strongly supported clade with *Deschampsia* in the ITS+ETS (100, 1; Figs 1, 4) and ITS trees (94; Figs 2, 18); the clade in the ITS tree also includes *Scribneria bolanderi* (not sampled in ITS+ETS analyses). We refer to this clade in the text as the “*Deschampsia* clade”. Affinities of the *Deschampsia* clade are unresolved in both nrDNA trees. Moreover, our analyses show that Holcinae, of which we sampled *Deschampsia*, *Vahlodea* and *Holcus*, is not monophyletic. A lineage corresponding to Holcinae p.p. in the ITS+ETS tree is represented by *Vahlodea*, which is included in a weakly supported clade with Poinae (Figs 1, 4), and in the ITS tree *Holcus* and *Vahlodea* form a strongly supported clade (Fig. 2, Suppl. material 4).

### Combined plastid analyses

The combined plastid tree (hereafter referred to as the plastid tree except when comparing and contrasting the combined plastid and single plastid region trees) includes all samples with data for at least three of the five plastid regions (Figs 3, 19–24, Suppl. material 6) and taxon sampling comparable to the ITS+ETS tree. Relationships in the plastid tree are mostly congruent with and better resolved than in the ITS+ETS and ITS trees, and there are instances of incongruence between nrDNA and plastid trees. We consider a taxon’s placement to be incongruent or discordant if it is part of different moderately to strongly supported clades in nrDNA and plastid trees. We did not conduct an incongruent length difference (ILD) test to characterize incongruence statistically because we did not conduct analyses with combined nrDNA and plastid data.

The plastid tree recovers Poeae chloroplast groups 1 (99, 1; Figs 3, 24) and 2 (100, 1; Figs 3, 19) with strong support. Poeae chloroplast group 1 consists of Agrostidinae p.p., Anthoxanthinae, Aveninae s.str., Brizinae, Calothecinae, Koeleriinae, Phalaridinae and Torreyochloinae. Phalaridinae and Torreyochloinae are sister taxa (85, .98; Figs 3, 24). Torreyochloinae is monophyletic (Fig. 24). A moderately to strongly supported clade (74, .99) includes the following four successively-diverging lineages: (1) *Calamagrostis pisinna* (Agrostidinae p.p.); (2) *Lagurus ovatus*; (3) Aveninae s.str. (84, 1); and (4) Koeleriinae excluding *L. ovatus* (95, 1) (Figs 3, 20, 21). *Lagurus ovatus*, Aveninae s.str. and Koeleriinae form a clade corresponding to Aveninae s.l. (83, .99; Figs 3, 20), and *L. ovatus* is the sister taxon of Aveninae s.str. + Koeleriinae (64, .95; Figs 3, 20). This placement of *L. ovatus* is discordant with the ITS tree (the taxon is not sampled in the ITS+ETS tree). Within Aveninae s.str., *Arrhenatherum* and *Avena* form a clade (79, 1; Fig. 20). Koeleriinae (excluding *Lagurus ovatus*) is strongly supported (95, 1; Figs 3, 20) and divided into two clades: Koeleriinae clade A (72, .99; Figs 3, 20) and Koeleriinae clade B (95, 1; Figs 3, 21). Within Koeleriinae clade A, the following three lineages diverge successively: (1) *Trisetum distichophyllum*; (2) *Avellinia michelii*, *T. flavescens* and *Rostraria pumila* (88, 1), with *T. flavescens* and *R. pumila* forming a maximally supported clade; and (3) a maximally supported clade including *Koeleria*, *Gaudinia fragilis* and Trisetum sect. Trisetaera (Figs 20). Within the latter clade, all species of Trisetum sect. Trisetaera and three species of *Koeleria* form a clade (84, 1; Fig. 20) with little internal resolution. Another clade includes the other three species of *Koeleria* (83, 1; Fig. 20). Relationships among these two lineages and *Gaudinia* are unsupported.

Koeleriinae clade B comprises *Calamagrostis*/*Deyeuxia* p.p. (species from Mexico and a subset of species from South America), *Graphephorum*, *Leptophyllochloa*, *Peyritschia*, *Sphenopholis*, Trisetum subg. Deschampsioidea and Trisetum sect. Trisetum p.p. (Fig. 21). Within Koeleriinae clade B, a large clade includes all but three species of *Calamagrostis*/*Deyeuxia* from South America that are part of Koeleriinae (51, .96; Fig. 21). *Sphenopholis* is the only genus resolved as monophyletic (93, 1; Fig. 21). *Graphephorum wolfii* and *Trisetum cernuum* (*Trisetum sect. Trisetum*) form a clade (70, .7; Fig. 21). Placement of this clade in Koeleriinae clade B conflicts with its placement in Koeleriinae clade A in the nrDNA trees. *Trisetum macbridei* and *T. irazuense* (*Trisetum sect. Trisetum*) are part of Koeleriinae clade B in the plastid tree, whereas they are part of Koeleriinae clade A in the ITS+ETS tree. A few lineages with more than one species receive some support in the plastid tree, but most relationships among taxa in Koeleriinae clade B are unresolved.

A large clade comprising Agrostidinae p.p., Anthoxanthinae, Brizinae and Calothecinae is strongly supported (99, 1; Figs 3, 24). Anthoxanthinae and Brizinae are successively diverging lineages sister to a strongly supported clade comprising Agrostidinae and Calothecinae (91, 1; Figs 3, 22–24). However, neither Agrostidinae nor Calothecinae are monophyletic. The Agrostidinae + Calothecinae clade includes four main lineages, all strongly supported: (1) Agrostidinae p.p. (*Calamagrostis coarctata*, *Echinopogon*) + Calothecinae p.p. (*Relchela*) (99, 1; Figs 3, 24); (2) Calothecinae p.p. (*Chascolytrum*) (93, 1; Figs 3, 24); (3) Agrostidinae p.p. (*Deyeuxia effusa*) (99, 1; Figs 3, 24); and (4) Agrostidinae p.p. (96, 1; Figs 3, 22–24), including most genera traditionally included in the subtribe. Calothecinae is not monophyletic in the plastid tree because *Chascolytrum* and *Relchela* do not form a clade.

There is no deep resolution within the large Agrostidinae p.p. clade in the plastid tree, although several clades of two or more species of *Calamagrostis*/*Deyeuxia* are identified. The branches that define each of these clades are very short. These clades and the multiple species of *Calamagrostis*/*Deyeuxia* not included in a clade form a polytomy along the Agrostidinae p.p. backbone. Furthermore, like in the nrDNA trees, *Ammophila* is not monophyletic. *Ammophila breviligulata* is part of a clade with multiple species of *Calamagrostis*/*Deyeuxia*, whereas *A. arenaria* is part of the polytomy. Multispecies clades of *Calamagrostis*/*Deyeuxia* in the plastid tree include (1) *C. epigeios*, *C. arundinacea*, *C. varia*, *C. pseudophragmites*, *C. rivalis* p.p., and C. × acutiflora (50, .97; Fig. 22); (2) *Ammophila breviligulata*, *C. purpurascens*, *C. rubescens* Buckley, *C. foliosa*, *C. sesquiflora* (Trin.) Kawano, *C. pickeringii*, *C. scopulorum* M.E. Jones, *C. koelerioides* Vasey, *C. howellii* Vasey, *C. cainii* and C. *guatemalensis* Hitchc. (75, .58; Fig. 22); (3) *C. llanganatensis* Laegaard and *C. carchiensis* Laegaard (84, 1; Fig. 22); and (4) *C. lapponica* (Wahlenb.) Hartm., C. stricta subsp. groenlandica, *C. deschampsioides*, *C. emodensis*, *C. macrolepis* Litv., *C. perplexa*, *C. epigeios* p.p., C. stricta subsp. stricta p.p., *C. nutkaensis*, C. stricta subsp. inexpansa (A. Gray) C.W. Greene, *C. stricta*, *C. rivalis*, *C. villosa*, C. × gracilescens and *C. canescens* (57, 1; Fig. 23). Another clade (51, 1; Figs 23, 24) includes 13 species of *Calamagrostis* (*C. arundinacea* p.p., *C. brachytricha*, *C. distantiflora*, *C. canadensis* (Michx.) P. Beauv., C. stricta subsp. inexpansa p.p., *C. porteri*, *C. angustifolia* Komarov, *C. phragmitoides* Hartman, *C. chalybaea* Fr., *C. lapponica* p.p., C. cf. purpurascens, *C. rubescens* and *C. montanensis* (Scribn.) Vasey) and a maximally supported clade including *Agrostis*, *Polypogon*, *Calamagrostis bolanderi*, *Podagrostis aequivalvis* (Trin.) Scribn. & Merr. and four other species of *Calamagrostis*/*Deyeuxia* (*D. tripilifera*, *D. nivicola*, *D. diffusa*, *D. mazzettii*) (Figs 22–24). The latter large clade includes four main lineages: (1) a strongly supported clade including *C. bolanderi* and *Podagrostis aequivalvis* (98, 1; Fig. 24); (2) a moderately supported clade including five species of *Calamagrostis*/*Deyeuxia* and *Agrostis rosei* Scribn. & Merr. (88, 1; Fig. 24); and (3) a large strongly supported clade including species of *Agrostis* and *Polypogon* (99, 1; Fig. 24). The *Agrostis* + *Polypogon* clade is divided into two maximally supported clades. One includes all species of *Agrostis* except *A. capillaris* L. p.p., *A. gigantea* Roth p.p. and *A. rosei*. The other includes three sublineages: (1) *A. capillaris* p.p. and *A. gigantea* p.p. (95, 1; Fig. 24); (2) *Polypogon australis* Brongn. and *P. interruptus* Kunth; and (3) *P. elongatus*, *P. monspeliensis* (L.) Desf. and *P. viridis* (Gouan) Breistr. (92, 1; Fig. 24).

The plastid tree includes exemplars from three subtribes of Poeae chloroplast 2: Holcinae, Loliinae and Poinae. As in the nrDNA trees, a subset of species of *Calamagrostis*/*Deyeuxia* from South America recognized in Deyeuxia sect. Stylagrostis are part of a strongly supported clade with *Deschampsia* (100, 1; Figs 3, 19). There is little deep structure within this clade. Moreover, Holcinae, of which we sampled *Deschampsia*, *Vahlodea* and *Holcus*, is not monophyletic because *Holcus* and *Vahlodea* form a maximally supported clade separate from the *Deschampsia* clade.

### Indels

Numerous small indels representing tandem repeats likely arose as a result of slipped-strand mispairing and were present in each plastid matrix except *matK*. These indels are highly homoplasious, thus we did not score them and do not discuss them further. Non-tandem repeat indels in the plastid matrices were also present. We did not score these as separate characters in the analysis, but summarize them briefly; we also mapped these onto the trees. Several unambiguous indels are present in the *psbK–psbI* intergenic spacer (Appendix 1, Suppl. material 1). One is a 247 bp deletion (298 bp in the aligned matrix gaps) present in 107 accessions of 59 species, including all species in Koeleriinae clade B, *Deyeuxia pulchella*, *Calamagrostis pisinna* and *Avellinia michauxii* (Figs 21, 22 Suppl. material 9). An 84 bp deletion (117 bp in the aligned matrix) is shared by six accessions of four species of *Calamagrostis* (*C. macrolepis*, C. stricta subsp. stricta, *C. perplexa* and *C. epigeios* p.p.) (Fig. 23, Suppl. material 9). A 10 bp deletion is shared by all accessions of *Agrostis*, *C. bolanderi*, *Deyeuxia diffusa*, *D. mazzettii*, *D. nivicola*, *D. tripilifera*, *Podagrostis aequivalvis* and all accessions of *Polypogon* (Agrostidinae p.p.; Fig. 24); *Anthoxanthum odoratum* L. (Anthoxanthinae; Fig. 24); *Arrhenatherum elatius* (L.) P. Beauv., *Avena fatua* L. and *A. sativa* L. (Fig. 20); *Briza minor* L. (Fig. 24, Suppl. material 9); *Gaudinia fragilis*, all accessions of *Koeleria* and Trisetum sect. Trisetaera, *Rostraria pumila*, *T. flavescens* and *T. distichophyllum* (Fig. 20). In the *atpF-H* intergenic spacer region, a 260–268 bp deletion (varying in length at the 5’-end) is shared by one individual of *Calamagrostis anthoxanthoides* and six genera of Poinae (Figs 19, 22, Suppl. material 9).

The 3’-end of the ETS region sampled here includes relatively conserved 5’- and 3’-ends and more rapidly evolving middle regions. There are several unambiguous indels in the ETS alignment (Appendix 1, Suppl. material 1), including a 63–67 bp insertion present in *Polypogon elongatus* and all accessions of *Agrostis* except *A. exarata*, *A. capillaris* and *A. gigantea* (Fig. 7), and a 12 bp deletion in all taxa of Aveninae s.str. and Koeleriinae (Fig. 5). Presence of the latter indel in *Helictotrichon*, however, is unclear because the 12 bp deletion overlaps with a 26 bp insertion present in the two *Helictotrichon* samples. An 81 bp insertion is present in *Gaudinia fragilis* and *Rostraria cristata* (L.) Tzvelev (Fig. 5). Species of *Anthoxanthum* share an 86–192 bp insertion (Fig. 9). All taxa of the Agrostidinae + Brizinae + Calothecinae clade share a 48–56 bp insertion (73 bp in the alignment) (Fig. 8). The clade is also defined by a 107–159 bp deletion (excluding the outgroup from the alignment, and 190 bp in the alignment, including gapped sites) (Fig. 8). Including *B. vulgaris*, the indel is 472 bp (426 bp in *B. vulgaris* excluding gapped sites). Each of these latter two indels includes additional substructure we have not attempted to describe.

## Discussion

Our broadly sampled molecular phylogenetic analyses of nrDNA and plastid DNA identify several major clades mostly corresponding to the subtribes of Poeae as now recognized. The biparentally-inherited tandemly repeated units of nrDNA are commonly used to reconstruct phylogenetic relationships because nrDNA is present in thousands of copies in plants and is readily PCR-amplified, and concerted evolution is believed to homogenize repetitive DNA sequences, either by gene conversion, unequal crossing over, or both, such that the repetitive sequences do not evolve independently of each other (Liao 1999). Indeed, the ITS region of nrDNA is commonly sequenced in phylogenetic relationships of grasses, as it is here. We also sequenced the ETS region, which has not previously been studied in most subtribes of Poeae chloroplast group 1, and explored the phylogenetic utility of combined ITS+ETS data in the group. Although the presence of large indels in the ETS region made parts of the alignment challenging, our results demonstrate that combining ETS with ITS is beneficial for clarifying the nrDNA phylogenetic history of Poeae at both deep and shallow parts of the tree. Resolution and support along the backbone of the combined ITS+ETS tree is better than that of the ITS tree, like in other phylogenetic studies using both markers (Poczai and Hyvönen 2010). Nevertheless, there are still some poorly supported branches in the ITS+ETS tree, and resolution of these will probably require greater amounts of sequence data from other regions of the nuclear genome.

**Figure 11.**
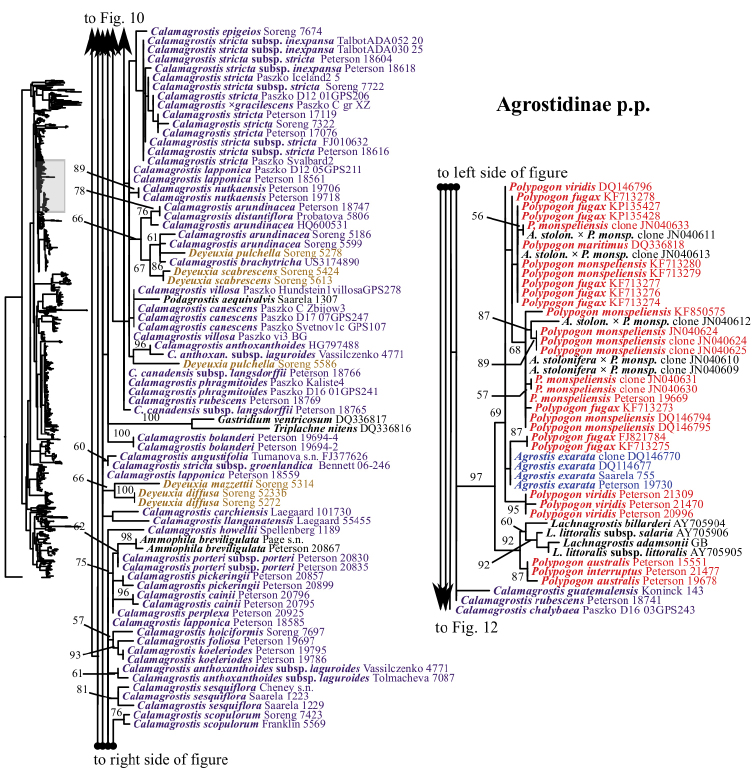
A portion (part of Agrostidinae p.p.) of the maximum likelihood phylogram inferred from ITS data. ML bootstrap support is recorded along branches when >50%. The shaded area of the smaller tree on the left indicates the location in the overall tree of the portion shown.

The large ITS tree we generated, incorporating the new and most relevant previously published data from the grass subtribes studied here, represents the most comprehensive sampling to date of Poeae chloroplast group 1. Although increased taxon sampling can increase phylogenetic accuracy (Zwickl and Hillis 2002), most aspects of relationship among major clades (i.e., deep relationships) in the ITS tree are unresolved, like in other studies (Quintanar et al. 2007, Saarela et al. 2010). Even with this limitation, however, this large ITS tree provides a useful overview of phylogenetic diversity of Poeae chloroplast group 1 because most samples are resolved in major clades. Furthermore, analysing together conspecific ITS sequences generated by independent workers provides new insight into infraspecific variation, and increases confidence in the accuracy of the sequences and of identifications of vouchers when the conspecific sequences group together. This is especially true for species represented in previous ITS trees by only single sequences (e.g., *Ammophila arenaria*, *Avellinia michelii*, *Briza maxima*, *Graphephorum wolfii*, *Lagurus ovatus*, *Sphenopholis obtusata* (Michx.) Scribn.).

Despite the generally higher rate of evolution of nrDNA compared to plastid DNA in plants and the widespread use of nrDNA for reconstructing phylogeny, caution is required when inferring phylogeny from nuclear ribosomal sequences (Alvarez and Wendel 2003). Concerted evolution of nrDNA may hide evidence of ancient or recent reticulation, polyploidization and recombination among copies, if polymorphic ITS copies are homogenized towards one of the repeat types, as has been demonstrated in multiple genera (Wendel et al. 1995, Fuertes Aguilar et al. 1999, Bao et al. 2010, Zozomová‐Lihová et al. 2014, Xu et al. 2017). As such, an inferred tree may not accurately reflect evolutionary history. Furthermore, multiple studies in diverse plant groups have demonstrated that concerted evolution within individuals is not always complete, resulting in within-individual polymorphisms (Matyášek et al. 2012, Simon et al. 2012, Song et al. 2012, Xu et al. 2017). Intra-individual polymorphisms are evident in chromatograms when more than one peak at site is present, and can be further characterized by both cloning (Xiao et al. 2010) and next-generation sequencing methods (Simon et al. 2012). Phylogenetic analysis of divergent copies (paralogs) can provide insight into evolutionary history. Both of these issues may be particularly problematic for grasses: all diploid grasses are considered paleopolyploids, and more than 60% of grasses are considered polyploids (neopolyploids) (Levy and Feldman 2002). Nevertheless, many grass phylogenetic studies have been based on ITS, in whole or in part, including studies of Poeae (see Suppl. material 2 for the list of studies that generated new ITS sequences included in analyses here), of which only a few characterized infraspecific variation in ITS by cloning (Grebenstein et al. 1998, Brysting et al. 2004, Reichman et al. 2006, Nikoloudakis et al. 2008, Winterfeld et al. 2009b, Rotter et al. 2010, Zapiola and Mallory-Smith 2012, Wölk and Röser 2014, 2017). Although we did not conduct cloning studies to characterize incomplete concerted evolution in the grasses studied here, this is an obvious avenue for future research. Study of low-copy nuclear genes is also needed, as these are biparentally and independently inherited and can be used to characterize reticulation within lineages. Low-copy nuclear genes have been explored in taxa of Poeae chloroplast group 1 in a few studies (Essi et al. 2008, Winterfeld et al. 2012, Wölk and Röser 2014, 2017, Hochbach et al. 2015, Minaya et al. 2015, Wölk et al. 2015).

**Figure 12.**
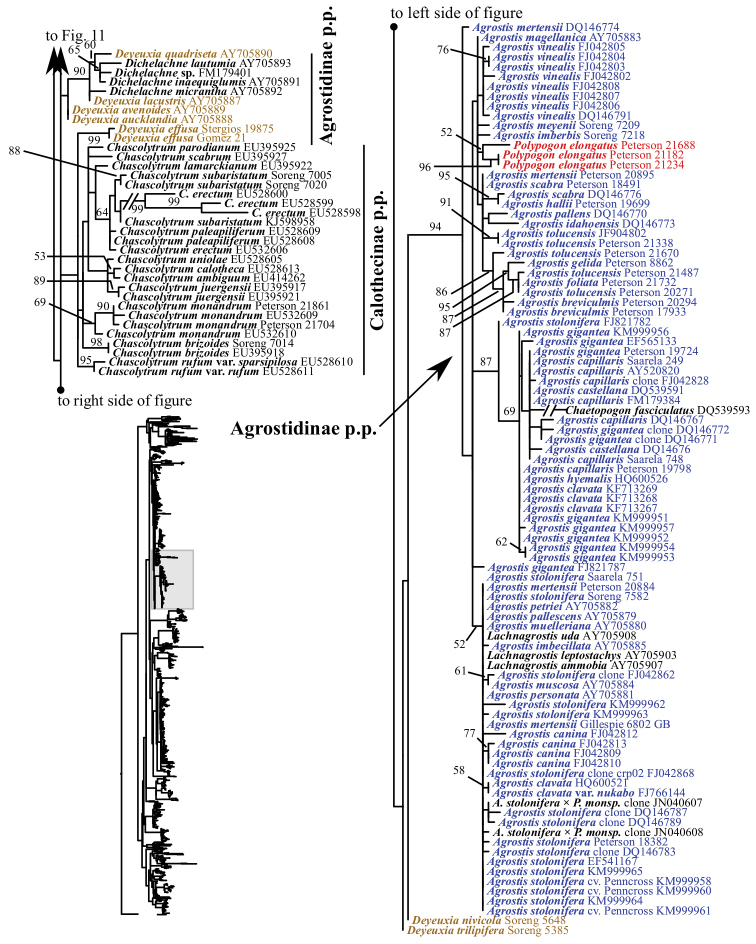
A portion (part of Agrostidinae p.p. and Calothecinae p.p.) of the maximum likelihood phylogram inferred from ITS data. ML bootstrap support is recorded along branches when >50%. The shaded area of the smaller tree on the left indicates the location in the overall tree of the portion shown.

We sequenced five plastid DNA regions, and like in other studies, the plastid analyses strongly support the clades referred to as Poeae chloroplast groups 1 and 2. Furthermore, strong backbone support within Poeae chloroplast group 1 is an improvement compared to plastid studies of the group based on fewer gene regions (Döring et al. 2007, Quintanar et al. 2007, Saarela et al. 2010, Wölk and Röser 2014, 2017). However, clades corresponding to Poeae chloroplast groups 1 and 2 are not recovered in the nrDNA trees. Instead, the ITS+ETS tree identifies a moderately to strongly supported clade including all subtribes of Poeae chloroplast group 1 plus Sesleriinae and Scolochloinae. Other trees based on nrDNA also found Sesleriinae and Scolochloinae to be closely related to subtribes of Poeae chloroplast group 1, but in those the clade is not as strongly supported as it is here (Quintanar et al. 2007, Gillespie et al. 2008, Saarela et al. 2010). The other subtribes of Poeae chloroplast group 2 do not form a clade in the ITS+ETS tree, as in previous studies of nrDNA (Quintanar et al. 2007, Saarela et al. 2010).

Some deep relationships within Poeae chloroplast group 1 are moderately to strongly supported in the plastid tree: Torreyochloinae and Phalaridinae are sister taxa, a large clade consists of the successively diverging lineages Anthoxanthinae, Brizinae and Agrostidinae + Calothecinae, and a large clade includes Aveninae s.l., Koeleriinae, *Lagurus* and *Calamagrostis pissina*. However, relationships among these three clades are unresolved. A sister group relationship between Torreyochloinae and Phalaridinae was first identified in a phylogeny based on complete plastomes (Saarela et al. 2015). Support for this relationship is poorer in the few-gene tree, especially ML bootstrap support, compared to the maximally supported Torreyochloinae + Phalaridinae clade in the plastome study. The ITS+ETS tree, however, identifies a conflicting topology: Torreyochloinae is part of a weakly supported clade with Agrostidinae, Brizinae and Calothecinae; Phalaridinae and Anthoxanthinae are excluded from this clade. Such a clade was not identified in the one previous nrDNA phylogeny, based on ITS, that sampled both Torreyochloinae and Phalaridinae, in which all deep branches of the tree were weakly supported or unresolved (Saarela et al. 2010). Hybridization may have been involved in the origin of Torreyochloinae, given its different affinities in plastid and nrDNA trees. A phylogeny based on low copy nuclear genes could be constructed to test this hypothesis.

**Figure 13.**
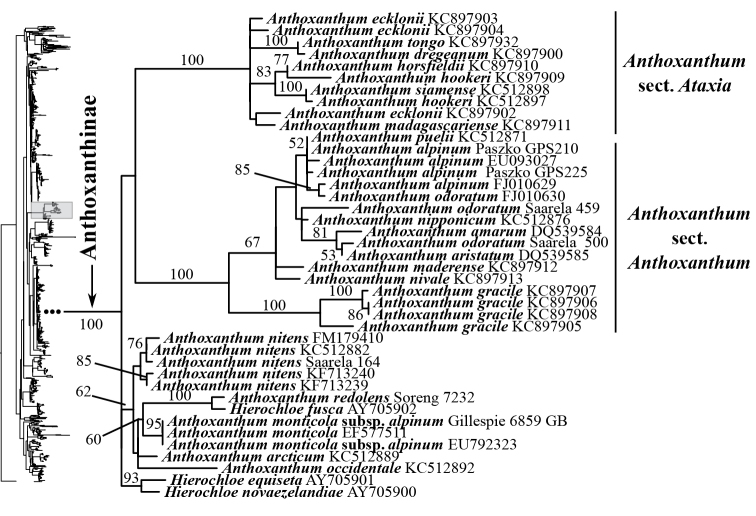
A portion (Anthoxanthinae) of the maximum likelihood phylogram inferred from ITS data. ML bootstrap support is recorded along branches when >50%. The shaded area of the smaller tree on the left indicates the location in the overall tree of the portion shown. The backbone branch represented by ellipses is shown only in Fig. 2.

Inclusion of Anthoxanthinae in a strongly supported clade with the Agrostidinae + Brizinae + Calothecinae clade in the plastid tree is congruent with a recent plastome phylogenomic study, in which the topology was maximally supported (Saarela et al. 2015), and with a recent few-gene plastid tree (Wölk and Röser 2017), in which the topology was weakly supported. Prior to the plastome study, however, this topology was found in plastid trees in only two other studies, with weak support in both (Bouchenak-Khelladi et al. 2008, Saarela et al. 2010). On the other hand, this topology is incongruent with other plastid, nuclear and combined trees, in which Anthoxanthinae is included in a clade with Aveninae, in some cases with only weak support (Davis and Soreng 2007, Döring et al. 2007, Quintanar et al. 2007, Schneider et al. 2009, Minaya et al. 2013). The topology of the ITS+ETS tree is not consistent with the plastid tree because in the former tree Anthoxanthinae form a weakly supported clade with Scolochloinae, Phalaridinae, Torreyochloinae, Agrostidinae, Brizinae and Calothecinae.

### Aveninae and Koeleriinae

Aveninae s.l. is a subtribe of annual and perennial grasses with lemmas awnless, mucronate or with an abaxial awn, awns geniculate and hila short or linear (Kellogg 2015). Aveninae s.l. is not monophyletic in nrDNA trees because Sesleriinae is nested within it. Support for the Aveninae s.l. + Sesleriinae clade is stronger in the ITS+ETS tree than in the ITS tree, and is further supported by a 12 bp deletion in the ETS alignment (data are missing for *Sesleria* in this part of the ETS matrix). In the plastid tree, Aveninae s.l. is monophyletic and Sesleriinae is part of Poeae chloroplast group 2. Support for the Aveninae s.l. clade in the combined plastid tree is higher than in most trees based on fewer plastid regions (e.g., Quintanar et al. 2007, Schneider et al. 2009, Saarela et al. 2010).

**Figure 14.**
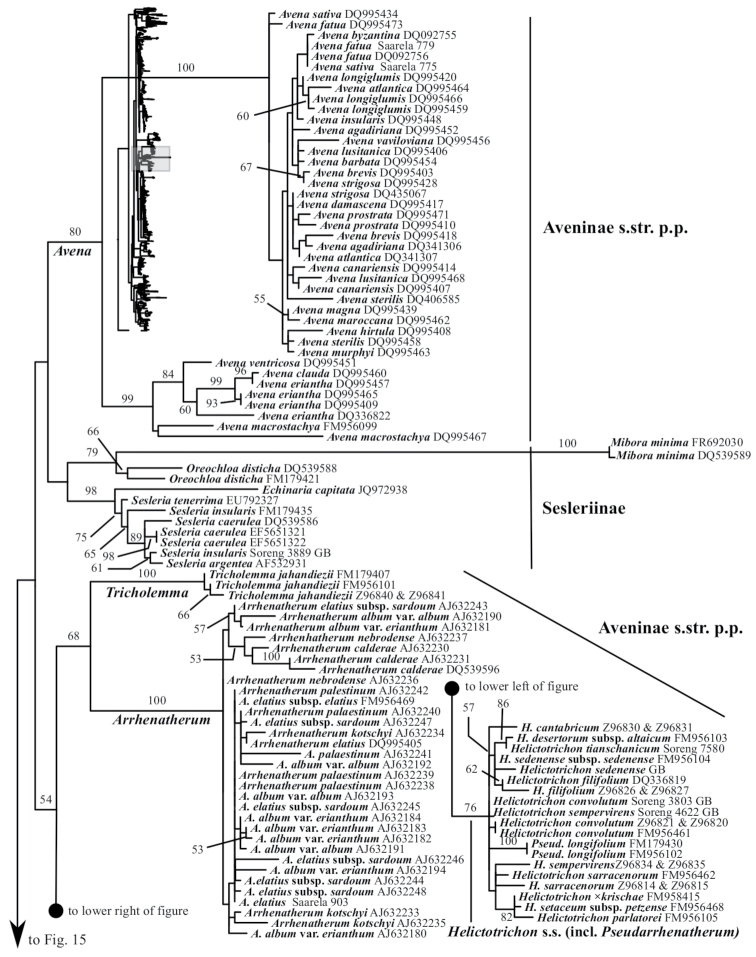
A portion (Aveninae s.str., Sesleriinae) of the maximum likelihood phylogram inferred from ITS data. ML bootstrap support is recorded along branches when >50%. The shaded area of the smaller tree on the upper left indicates the location in the overall tree of the portion shown.

Aveninae s.l. is divided into two subclades. In ITS+ETS and plastid trees, one subclade includes *Arrhenatherum*, *Avena* and *Helictotrichon* s.str. (Aveninae s.str.); in the nrDNA trees, *Sesleria* (Sesleriinae) is part of this lineage. In the ITS tree, the equivalent subclade includes *Arrhenatherum*, *Avena*, *Helictotrichon* and *Tricholemma* (not sampled in the ITS+ETS tree). Within the clade in the ITS tree, *Arrhenatherum*, *Avena* and *Helictotrichon* s.str. are densely sampled, each genus is resolved as monophyletic, *Arrhenatherum* and *Tricholemma* are sister taxa, and *Arrhenatherum* + *Tricholemma* and *Helictotrichon* are sister groups, as in other studies (e.g., Quintanar et al. 2007, Wölk and Röser 2014). In the ITS+ETS tree, however, *Avena* and *Helictotrichon* are sister taxa. This different placement for *Helictotrichon* compared to the ITS tree may be due to the poorer taxon sampling in the ITS+ETS tree, conflicting signal in the ITS and ETS regions, or both. In the plastid tree, *Avena* and *Arrhenatherum* form a clade, a topology consistent with an earlier combined nrDNA and plastid tree (Winterfeld et al. 2009a), but conflicting with the ITS and ITS+ETS trees. In a recent plastid tree, relationships among *Avena*, *Helictotrichon*, *Arrhenatherum* and ×*Trisetoptrichon* Röser & A. Wölk (*Helictotrichon* × *Trisetopsis*) are mostly unresolved (Wölk and Röser 2017).

The second subclade of Aveninae s.l. is recovered in the ITS+ETS and plastid trees with moderate to strong support. This clade has been recovered in previous studies based on plastid and nuclear data (Saarela et al. 2010, Wölk and Röser 2017), and corresponds to Koeleriinae (type: *Koeleria*) *sensu* Quintanar et al. (2007, 2010). All taxa in this clade share an eight bp deletion in ITS 1, an informative indel first noted by Quintanar et al. (2007) in a subset of the taxa sampled here. Because a considerable portion of our results focus on the latter subclade, and because being able to refer to this lineage with a rank-based scientific name rather than an informal one facilitates clear communication, we accept Koeleriinae as a subtribe separate from Aveninae s.str., following Quintanar et al. (2007, 2010). Moreover, recognition of Aveninae s.str. and Koeleriinae as subtribes results in a classification consistent with both nrDNA and plastid trees. However, if Koeleriinae is included in Aveninae s.l., as in Soreng et al. (2015b), Aveninae s.l. is paraphyletic in the context of nrDNA trees given the placement of Sesleriinae in those trees.

**Figure 15.**
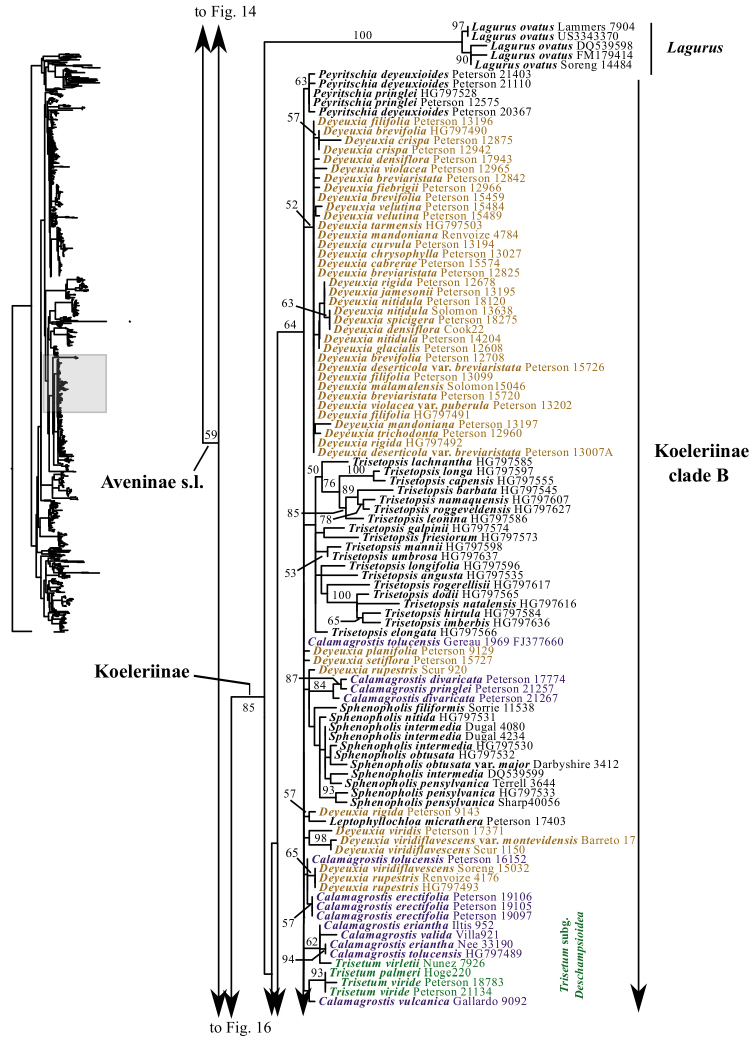
A portion (*Lagurus*, Koeleriinae clade B) of the maximum likelihood phylogram inferred from ITS data. ML bootstrap support is recorded along branches when >50%. The shaded area of the smaller tree on the left indicates the location in the overall tree of the portion shown.

Relationships in Koeleriinae in the trees reported here are generally congruent with previous phylogenetic studies of the subtribe. Quintanar et al. (2007) sampled seven species of *Koeleria*, six of *Rostraria*, 14 of *Trisetum* and five of *Trisetaria*, and species of each genus were intermixed with each other and with species of *Avellinia*, *Gaudinia* and *Graphephorum* in ITS and plastid trees. Saarela et al. (2010) increased sampling of New World species of Koeleriinae, and identified two major clades of Koeleriinae in an ITS tree. They referred to these clades as the “Old World *Trisetum* Alliance” and the “New World *Trisetum* Alliance”, because species of *Trisetum* were present in each clade and most species in each clade were from either the Old or New World. These major clades are strongly supported in the current ITS+ETS and plastid trees, and we refer to them more simply as Koeleriinae clade A and Koeleriinae clade B, respectively. In Wölk and Röser (2017), Koeleriinae clade B is resolved in both ITS and plastid trees, but Koeleriinae clade A is resolved only in their ITS tree. In our analyses, Koeleriinae clade A includes *Avellinia*, *Gaudinia*, *Koeleria*, *Rostraria*, *Trisetaria* and *Trisetum* p.p. We did not sample the new genus *Tzveleviochloa*, with two species, recently described by Wölk and Röser (2017), which is also part of Koeleriinae clade A. Koeleriinae clade B includes many taxa of *Calamagrostis*/*Deyeuxia* sampled from Mexico, Central and South America, *Leptophyllochloa*, *Peyritschia*, *Sphenopholis*, *Trisetum* p.p., and *Trisetopsis*. A 247 bp deletion in the *psbK-psbI* intergenic spacer region is present in all taxa of Koeleriinae clade B except *Deyeuxia tripilifera*, and in two taxa not part of this clade, *Avellinia michauxii* and *Calamagrostis pisinna* (sister to Aveninae s.l.). The distribution of this indel indicates it may be symplesiomorphic in the clade comprising *C. pisinna* and Aveninae s.l. We also identify a lineage of Koeleriinae in the ITS and *matK* (Suppl. material 7) trees comprising two species classified in Trisetum subsect. Sibirica (Veldkamp and van der Have 1983): *T. bifidum* (Thunb.) Ohwi, from China, eastern Asia and Papuasia, and *T. sibiricum* Rupr., from Eurasia and northwestern North America. A similar lineage of these two species is identified in the ITS tree in Wölk and Röser (2017). Relationships among this lineage and Koeleriinae clades A and B are unresolved in the trees. Increased sampling of genes and taxa is needed to better resolve the placement of this lineage and to identify all the species that are part of it.

**Figure 16.**
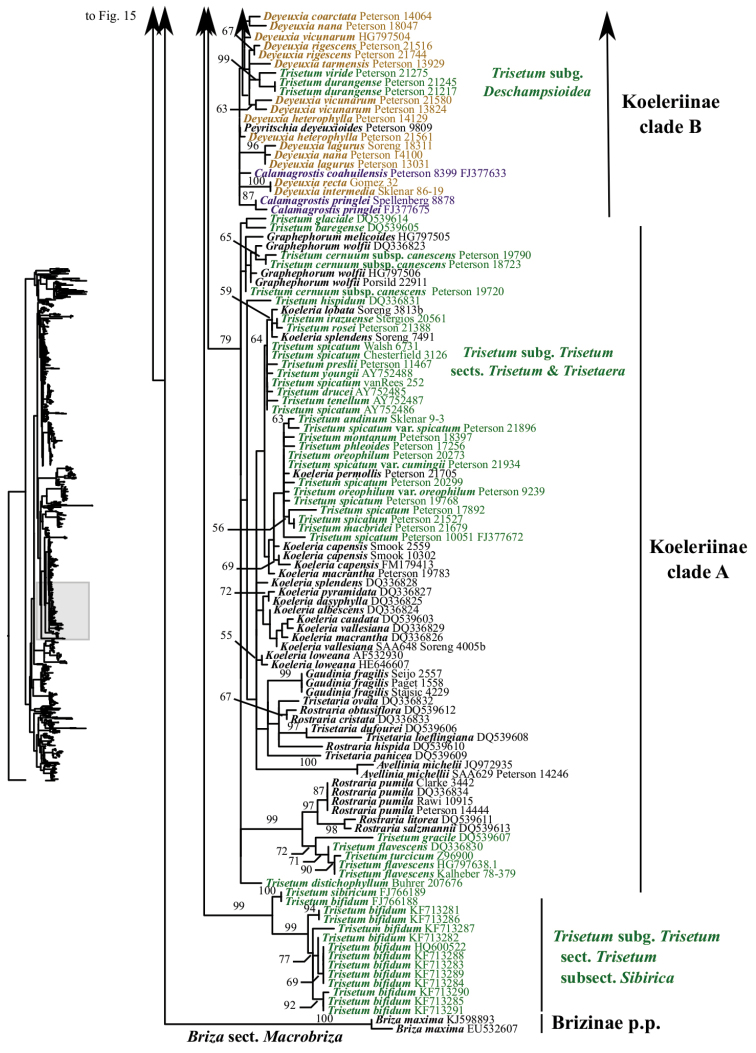
A portion (Koeleriinae clade A, part of Koeleriinae clade B, Trisetum subsect. Sibirica and Brizinae p.p.) of the maximum likelihood phylogram inferred from ITS data. ML bootstrap support is recorded along branches when >50%. The shaded area of the smaller tree on the left indicates the location in the overall tree of the portion shown.

### Koeleriinae Clade A

#### *Trisetum* p.p., *Trisetaria*, *Koeleria*, *Rostraria*, *Avellinia* and *Gaudinia*

Circumscription of *Trisetum* and *Trisetaria* has been problematic. *Trisetum* is a worldwide, temperately-distributed genus of 70 to 96 perennial species generally characterized by having first glumes one- to three-nerved, second glumes three- to five-nerved, lemma apices with two to four short awns with the central awn usually inserted above the middle of the lemma (sometimes near the middle), paleas not tightly enclosed by the lemma and an androecium of three stamens (Finot et al. 2004, Simon 2014). *Trisetum flavescens* is the lectotype of the genus (Hitchcock 1920, Committee for Spermatophyta 1987). A group of ca. 15 annual species, characterized by having small spikelets, contracted panicles and distributed mostly in the Mediterranean, has been variously included in *Trisetum* (e.g., Quintanar et al. 2007), sometimes as Trisetum subg. Trichaeta (P. Beauv.) Rchb., or in *Trisetaria* (Clayton and Renvoize 1986, Clayton et al. 2006 onwards, Quintanar et al. 2010), as treated here. The type species of *Trisetaria* is *T. linearis* Forssk. When combined in a single genus, the name *Trisetaria* (Forsskål and Niebuhr 1775) has priority over *Trisetum* (Persoon 1805), as treated in a revision of the group in Spain by Paunero R. (1950). A synopsis of the subdivision of *Trisetum* was presented by Quintanar et al. (2010), but no detailed global synthesis or classification of *Trisetum* exists, nor have classifications of the genus been examined explicitly in a molecular context.

**Figure 17.**
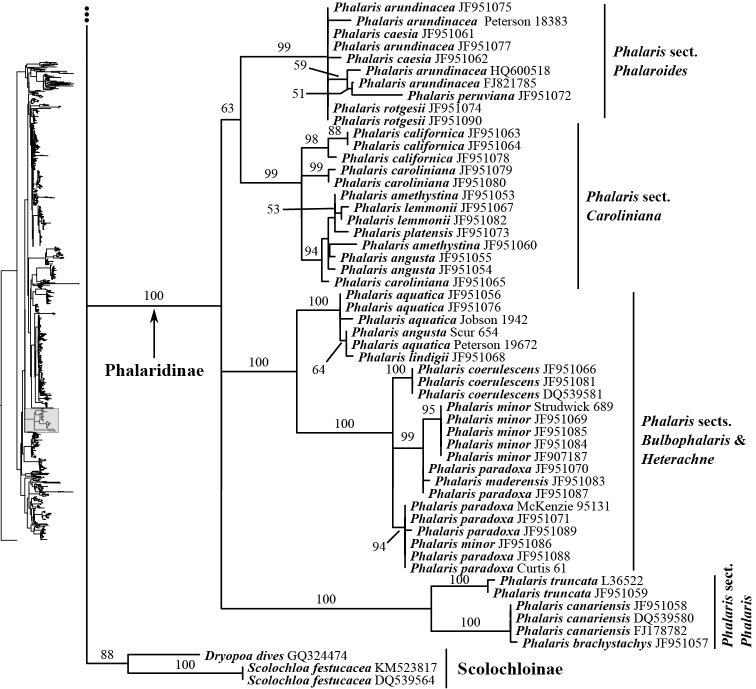
A portion (Phalaridinae and Scolochloinae) of the maximum likelihood phylogram inferred from ITS data. ML bootstrap support is recorded along branches when >50%. The shaded area of the smaller tree on the left indicates the location in the overall tree of the portion shown. The subdivisional classification of *Phalaris* follows Voshell et al. (2016). The backbone branch represented by ellipses is shown only in Fig. 2.

Classifications of *Trisetum* in the Old World have been proposed by numerous authors. Ascherson and Graebner (1899) recognized Trisetum sect. Eutrisetum Asch. & Graebn., nom. inval. (=Trisetum sect. Trisetum) and Trisetum sect. Trisetaera Asch. & Graebn. Trisetum sect. Trisetaera included only the type, *T. spicatum*, and was distinguished by having a contracted panicle and densely pubescent panicle rachises, features also characteristic of *Koeleria*. Hermann (1956) recognized four informal groups in the genus: *Trisetum*, “Ventenata” (now understood to be a genus of subtribe Poinae), *Rostraria*, and “Argentaria”, with two species, *T. distichophyllum* and *T. argenteum* (Willd.) Roem. & Schult. Chrtek and Jirásek (1963) removed three western Mediterranean species (*T. glaciale* (Bory) Boiss., *T. gracile* E. Fourn., *T. antoni-josephii* Font Quer & Munoz Medina) from Trisetum sect. Trisetum and placed them in Trisetum sect. Gracilia Chrtek & Jirásek, based on leaf anatomical characteristics. Chrtek (1965) later recognized four subgenera of *Trisetum* in Europe, differing in leaf anatomy: (1) Trisetum subg. Trisetum; (2) Trisetum subg. Distichotrisetum Chrtek, comprising *T. distichophyllum* (type) and *T. argenteum* from central to eastern Europe; (3) Trisetum subg. Glaciotrisetum Chrtek, comprising *T. glaciale* (type) and *T. antoni-josephii* from southern Spain; and (4) Trisetum subg. Graciliotrisetum Chrtek (=Trisetum sect. Gracilia), comprising *T. gracile* from Sardinia. Chrtek (1965) recognized five sections in Trisetum subg. Trisetum: (1) Trisetum sect. Trisetum, comprising most species of the genus; (2) Trisetum sect. Trisetaera, comprising *T. spicatum*; (3) Trisetum sect. Rigida Chrtek, comprising *T. rigidum* (Bieb.) Roem. & Schult., *T. macrotrichum* Hack., *T. buschianum* Seredin and *T. transcaucasicum* Seredin from Eurasia (Tzvelev 1976); (4) Trisetum sect. Hispanica Chrtek, comprising *T. velutinum* Boiss. and *T. hispidum* Lange from the Iberian Peninsula; and (5) Trisetum sect. Carpatica Chrtek, comprising *T. fuscum* (Kit.) Roem. & Schult. from the Carpathians. Chrtek (1968) later described Trisetum sect. Trisetum series Sibirica Chrtek (=Trisetum subsect. Sibirica (Chrtek) Prob.) comprising *T. sibiricum* (type) and *T. turcicum* Chrtek. Other sectional taxa of *Trisetum* are synonyms in other genera, including Trisetum sect. Avenula Dumort. (=*Avenula*), Trisetum sect. Colobanthus (Trin.) Rchb. (=*Sphenopholis*), Trisetum sect. Discolops Dumort. (=*Aira* L.) and Trisetum sect. Koeleria E. Desv. (=*Koeleria*). All of the Old World species of *Trisetum* included in previous phylogenetic studies are part of Koeleriinae clade A (Quintanar et al. 2007, Saarela et al. 2010). None of the four species of Trisetum sect. Rigida (Tzvelev, 1976) have been included in phylogenetic studies, nor have most species from New Zealand (Edgar 1998) and China (Chrtek 1990, Wu and Philips 2006).

**Figure 18.**
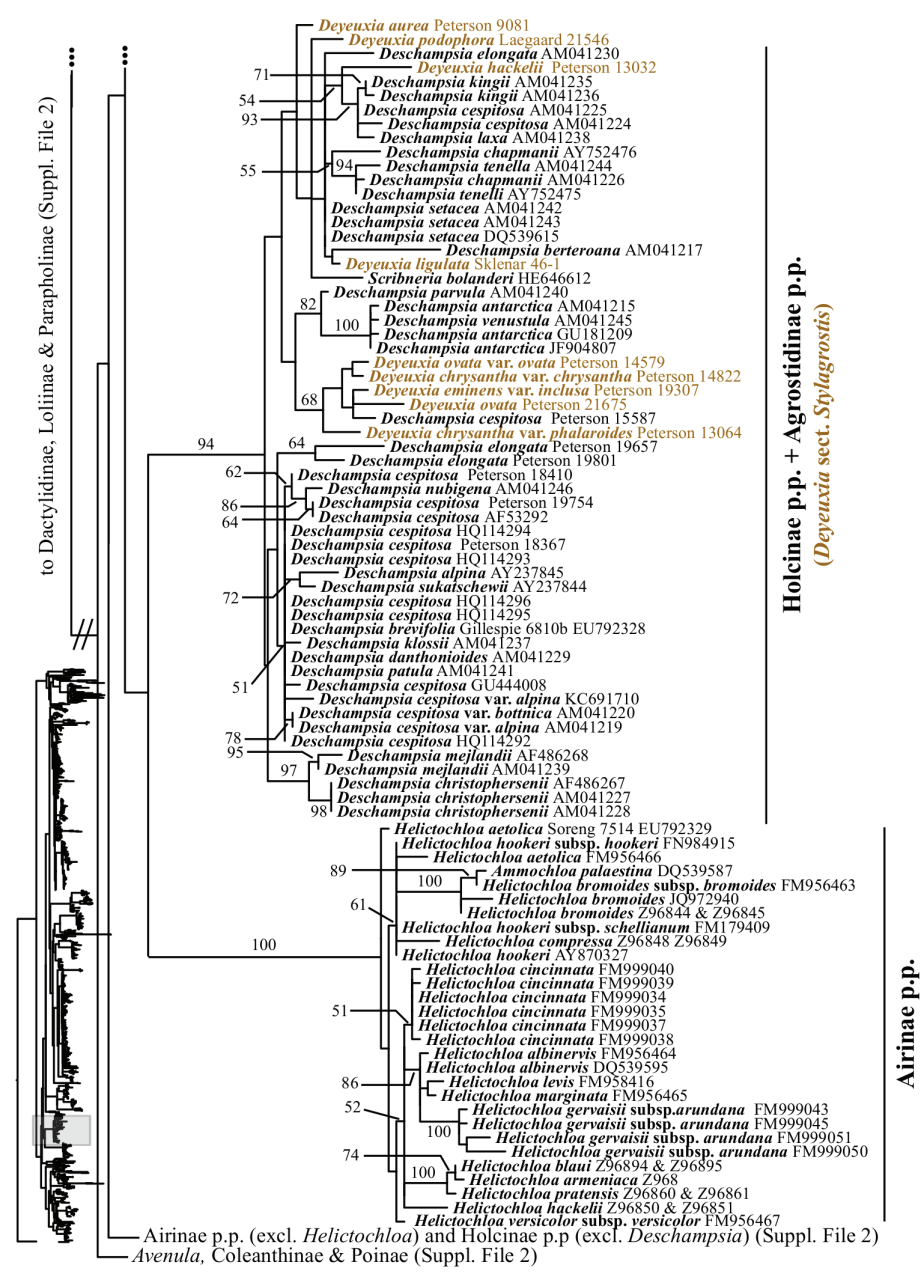
A portion (Agrostidinae p.p., Holcinae p.p. and Airinae p.p.) of the maximum likelihood phylogram inferred from ITS data. ML bootstrap support is recorded along branches when >50%. The shaded area of the smaller tree on the bottom left indicates the location in the overall tree of the portion shown. Backbone branches represented by ellipses are shown only in Fig. 2.

Classifications of *Trisetum* in the New World have been proposed by numerous authors. Early treatments of *Trisetum* for North, Central and South America include those of Steudel (1854), Hitchcock (1927a) and Louis-Marie (1928). *Trisetum* and allies were recently revised in the New World, and classified in Trisetum subg. Trisetum sects. *Trisetum* and *Trisetaera* and Trisetum subg. Deschampsioidea (Finot et al. 2004, 2005a, 2005b, 2006b). In the New World, seven species of Trisetum sect. Trisetum are recognized: *T. cernuum*, *T. curvisetum* Morden & Valdés-Reyna, *T. flavescens* (introduced), *T. irazuense*, *T. montanum* Vasey, *T. orthochaetum* Hitch., and *T. sibiricum*; and 14 of Trisetum sect. Trisetaera (Finot et al. 2004, 2005a, 2005b). Trisetum subg. Deschampsioidea was previously recognized as Trisetum subsect. Deschampsioidea Louis-Marie (1928), with three species mentioned in the protologue (two are species of *Deschampsia*). Finot et al. (2004) raised the subsection to the rank of subgenus, designated *T. palmeri* as its lectotype, and recognized eight Mexican species: *T. durangense* Finot & P.M. Peterson, *T. martha-gonzaleziae* P.M. Peterson & Finot, *T. palmeri*, *T. pinetorum* Swallen, *T. spellenbergii* Soreng, Finot & P.M. Peterson, *T. tonduzii* Hitchc., *T. viride* (Kunth) Kunth and *T. virlettii* E. Fourn. Trisetum subg. Deschampsioidea is distinguished from Trisetum subg. Trisetum by having lemma apices hyaline, without nerves or with both intermediate and marginal nerves extended beyond the apex as four short awns (vs. lemma apices opaque, the intermediate nerves extended beyond the apex as two short awns) and awns inserted on the middle of the lemma (vs. awns inserted on the upper third of the lemma) (Finot et al. 2004). Species of Trisetum subg. Deschampsioidea are part of Koeleriinae clade B (Saarela et al. 2010, Wölk and Röser 2014).

**Figure 19.**
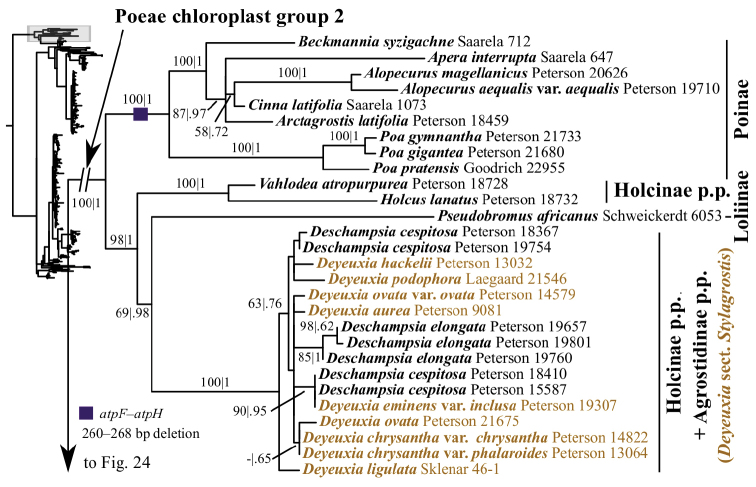
A portion (Agrostidinae p.p., Holcinae, Loliinae and Poinae) of the maximum likelihood phylogram inferred from combined plastid data (*atpF–atpH*, *psbK–psbI*, *psbA–rps19–trnH*, *matK*, *trnL–trnF*). ML bootstrap support (left) and BI poster probabilities (right) are recorded along branches. No support is shown for branches with bootstrap support <50% and posterior probability <.5. The shaded area of the smaller tree on the left indicates the location in the overall tree of the portion shown. Slashes (//) identify a branch shortened for presentation. An indel in *atpF–atpH* is mapped onto the phylogram.

The genera *Koeleria* Persoon (1805) and *Rostraria* Trinius (1820) are closely related to each other and to *Trisetum*, and have been variously circumscribed. *Koeleria* consists of ca. 47 meso- to xerophytic perennial species (Clayton et al. 2006 onwards) distributed in temperate regions around the world. *Koeleria* differs from *Trisetum* by having lemmas muticous, mucronate or inconspicuously-awned apically or subapically (Cafferty et al. 2000, Quintanar et al. 2010). The taxonomic history of *Koeleria* is reviewed in detail by Quintanar and Castroviejo (2013). *Koeleria* and *Trisetum* are known to hybridize. Hybrids between *Koeleria asiatica* Domin and *Trisetum agrostideum* (Laest.) Fr. from Asia have been described in the nothogenus ×*Trisetokoeleria* Tzvelev (Tzvelev 1971 [1970]). *Rostraria* includes ca. 13 annual species from the Mediterranean and Middle East (Clayton et al. 2006 onwards). Clayton and Renvoize (1986) considered *Rostraria* to be “an annual derivative of *Koeleria*” with more developed awns. Domin (1907) treated *Rostraria* as a synyonym of *Koeleria*, and placed its species in four subsections of Koeleria subg. Lophochloa (Rchb.) Domin. Species of *Koeleria* and *Rostraria* have consistently been resolved as part of Koeleriinae clade A (Davis and Soreng 2007, Quintanar et al. 2007, Saarela et al. 2010, Schaefer et al. 2011, Grass Phylogeny Working Group II 2012, Minaya et al. 2013).

**Figure 20.**
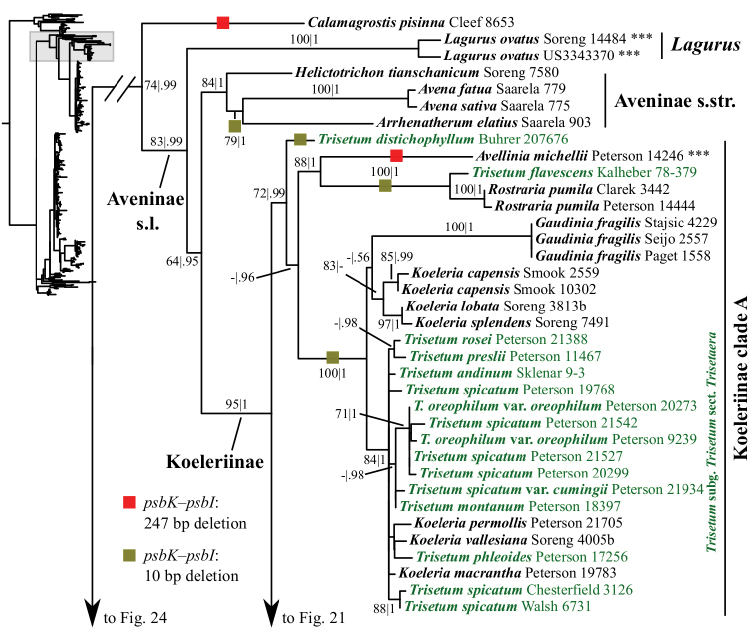
A portion (*Calamagrostis pisinna*, *Lagurus*, Aveninae s.str. and Koeleriinae clade A) of the maximum likelihood phylogram inferred from combined plastid data (*atpF–atpH*, *psbK–psbI*, *psbA–rps19–trnH*, *matK*, *trnL–trnF*). ML bootstrap support (left) and BI poster probabilities (right) are recorded along branches. No support is shown for branches with bootstrap support <50% and posterior probability <.5. A dash indicates bootstrap support <50%. The shaded area of the smaller tree on the left indicates the location in the overall tree of the portion shown. Placements of samples with asterisks (***) are incongruent in nrDNA and plastid trees. Slashes (//) identify a branch shortened for presentation. Two indels in *psbK–psbI* are mapped onto the phylogram.

*Avellinia* and *Gaudinia* are also closely related to the above-mentioned genera. *Avellinia* comprises two annual Mediterranean species, *A. michelii* (Savi) Parl. (2*n*=14, Rice et al. 2015) and *A. festucoides* (Link) Valdés & H. Scholz (Watson and Dallwitz 1992 onwards), characterized by having narrow spike-like or slightly loose panicles, spikelets with a rachilla extension, first glumes nearly bristle-like and second glumes longer than the lemma (Jessop et al. 2006). Both species have been treated in other genera: *Avellinia michelii* in *Trisetaria* as *T. michelii* (Savi) D. Heller (Clayton and Renvoize 1986), and *A. festucoides* in *Rostraria* as *R. festucoides* (Link) Romero Zarco (Romero Zarco 1996, Clayton et al. 2006 onwards). *Avellinia* has been sampled in only a few molecular studies, in which it is resolved in Koeleriinae clade A (Quintanar et al. 2007, Minaya et al. 2013).

*Gaudinia* is a small genus of four annual or biennial species endemic to the Mediterranean (Clayton and Renvoize 1986), distinguished by having fragile bilateral raceme inflorescences with spikelets that are sessile, several-flowered and disarticulating below the glumes, and three of the four species have a dorsal geniculate awn (*G. hispanica* Stace & Tutin is awnless) (Clayton and Renvoize 1986). Three of the four species of *Gaudinia* have previously been included in molecular studies (*G. fragilis*, *G. coarctata* T. Durand & Schinz and *G. hispanica*). Two studies included two species of *Gaudinia* (Soreng et al. 2007, Schaefer et al. 2011) where they formed a clade, whereas most included only one species (Soreng and Davis 2000, Quintanar et al. 2007, Bouchenak-Khelladi et al. 2008, Grass Phylogeny Working Group II 2012, Hochbach et al. 2015, Wölk and Röser 2017). In all these phylogenetic studies, *Gaudinia* is resolved in Koeleriinae clade A.

**Figure 21.**
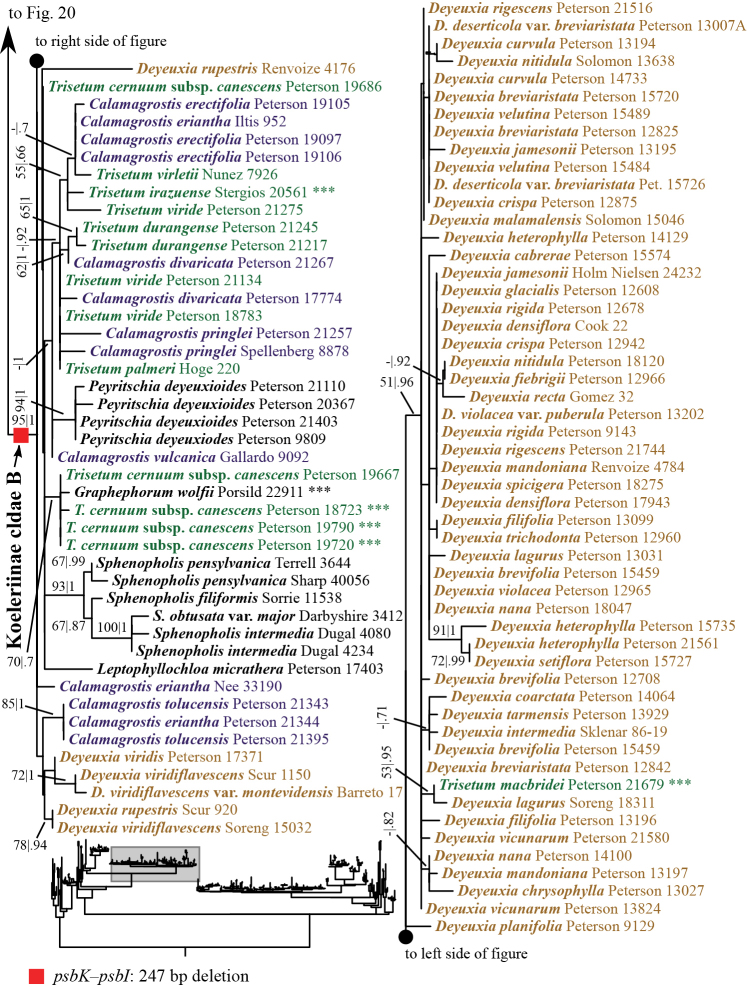
A portion (Koeleriinae clade B) of the maximum likelihood phylogram inferred from combined plastid data (*atpF–atpH*, *psbK–psbI*, *psbA–rps19–trnH*, *matK*, *trnL–trnF*). ML bootstrap support (left) and BI poster probabilities (right) are recorded along branches. A dash indicates bootstrap support <50%. No support is shown for branches with bootstrap support <50% and posterior probability <.5. The shaded area of the smaller tree on the bottom left indicates the location in the overall tree of the portion shown. Placements of samples with asterisks (***) are incongruent in nrDNA and plastid trees. An indel in *psbK–psbI* is mapped onto the phylogram.

Several strongly supported lineages in Koeleriinae clade A are identified in our analyses. In the ITS+ETS tree, one strongly supported clade includes the North American species *T. cernuum* (Trisetum subg. Trisetum sect. Trisetum) and *Graphephorum wolfii*, which are sister taxa, and the European species *T. distichophyllum* (Trisetum subg. Distichotrisetum). In the ITS tree, the two sampled species of *Graphephorum* are part of Koeleriinae clade A. In the plastid tree, however, *T. cernuum* and *G. wolfii* are part of Koeleriinae clade B. These discordant placements of *Graphephorum* and *T. cernuum* within Koeleriinae in nrDNA and plastid trees are consistent with earlier studies (Quintanar et al. 2007, Wölk and Röser 2014, 2017). Moreover, in phylogenies of the nuclear gene *topo6*, some clones of *topo6* from *G. melicoides* are part of a lineage corresponding to Koeleriinae clade A, and other clones are part of a lineage corresponding to Koeleriinae clade B (Wölk and Röser 2014, 2017). *Graphephorum* Desvaux (1810), a small genus of two species (*G. melicoides* (Michx.) Desv., *G. wolfii*) endemic to North America, differs from *Trisetum* in having an entire lemma apex, the dorsal awn reduced to a subapical mucro, and paleas tightly enclosed by the margins of the lemma (Finot et al. 2005a). The species of *Graphephorum* have been included in *Trisetum* (Louis-Marie 1928, Rumely 2007). Given the evidence from plastid, ribosomal and non-ribosomal nuclear DNA, the two species of *Graphephorum* and *T. cernuum* are probably of hybrid origin, although the parental species from which they might have arisen in Koeleriinae clades A and B are unknown. We are not aware of chromosome counts for *Graphephorum*, but they are likely polyploid given the multiple copies of *topo6* identified by Wölk and Röser (2014).

**Figure 22.**
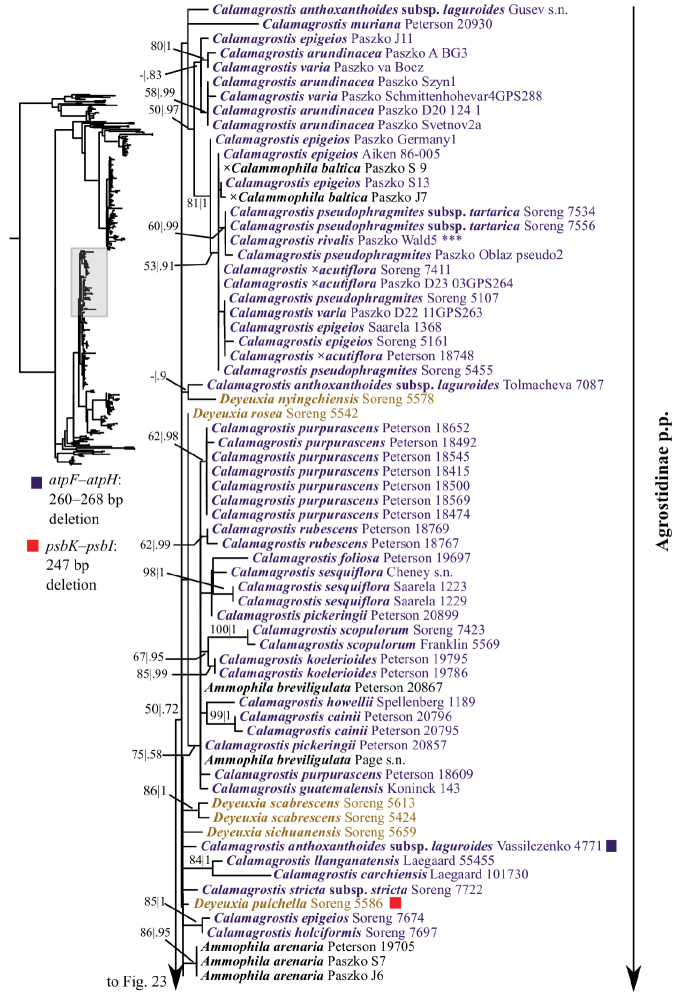
A portion (part of Agrostidinae p.p.) of the maximum likelihood phylogram inferred from combined plastid data (*atpF–atpH*, *psbK–psbI*, *psbA–rps19–trnH*, *matK*, *trnL–trnF*). ML bootstrap support (left) and BI poster probabilities (right) are recorded along branches. A dash indicates bootstrap support <50%. No support is shown for branches with bootstrap support <50% and posterior probability <.5. The shaded area of the smaller tree on the left indicates the location in the overall tree of the portion shown. Placement of the sample with asterisks (***) is incongruent in nrDNA and plastid trees. One indel in *psbK–psbI* and one in *atpF–atpH* are mapped onto the phylogram.

In the ITS+ETS tree, a second strongly supported clade within Koeleriinae clade A includes four successively diverging and moderately to strongly supported lineages: (1) *Trisetum flavescens* and *Rostraria pumila*; (2) *Avellinia michelii*; (3) *Gaudinia fragilis*; and (4) Trisetum sect. Trisetaera, *Koeleria* and *T. irazuense*, a species from Central and South America classified in Trisetum sect. Trisetum (Finot et al. 2005b). This topology is similar to the better-sampled ITS tree in Quintanar et al. (2007), who identified a clade of three species of *Trisetum* (*T. flavescens*, *T. turcicum*, *T. gracile*) sister to a clade of three species of *Rostraria* (*R. litorea*, *R. salzmannii*, *R. pumila*), and a clade comprising four species of *Trisetaria* (*T. duforei*, *T. loeflingiana*, *T. ovata* (Pers.) Paunero, *T. panicea* (Lam.) Paunero), three of *Rostraria* (*R. obtusiflora*, *R. hispida* (Savi) Doğan, *R. cristata*) and *Gaudinia fragilis*. It is also similar to the ITS tree in Wölk and Röser (2017), who also found *Trisetaria aurea* and *T. linearis* (type of *Trisetaria*) to form a clade with *Trisetum flavescens*. We did not sample any species of *Trisetaria* in the ITS+ETS tree. In the ITS tree, deep relationships in the clade are mostly unresolved; many species and multi-species clades form a polytomy. Moderate bootstrap support for the Trisetum sect. Trisetaera + *Koeleria* + *T. irazuense* lineage in the ITS+ETS tree is higher than bootstrap support for the equivalent clade in earlier ITS trees (Quintanar et al. 2007, Saarela et al. 2010). However, *T. irazuense* is part of Koeleriinae clade B in the plastid tree. As such, this species may be of hybrid origin, and its polyploid cytology (2*n*=28, 42) (Finot et al. 2005b) is consistent with this possibility. Persson and Rydin (2016) similarly found a different ITS accession of *T. irazuense* to be part of Koeleriinae clade A, but they did not obtain plastid data for their sample.

**Figure 23.**
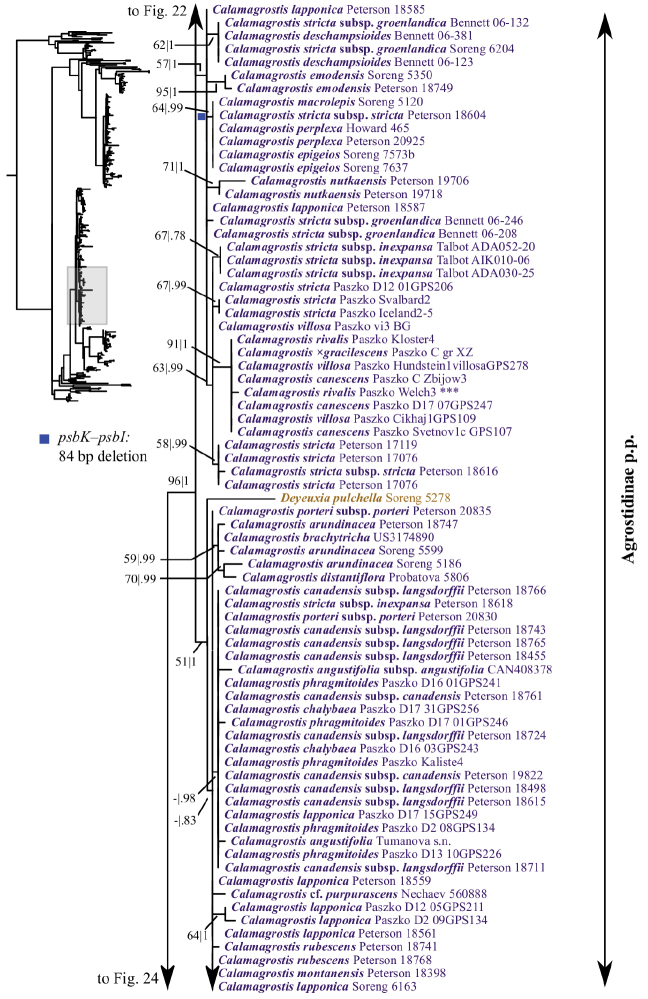
A portion (part of Agrostidinae p.p.) of the maximum likelihood phylogram inferred from combined plastid data (*atpF–atpH*, *psbK–psbI*, *psbA–rps19–trnH*, *matK*, *trnL–trnF*). ML bootstrap support (left) and BI poster probabilities (right) are recorded along branches. A dash indicates bootstrap support <50%. No support is shown for branches with bootstrap support <50% and posterior probability <.5. The shaded area of the smaller tree on the left indicates the location in the overall tree of the portion shown. Placement of the sample with asterisks (***) is incongruent in nrDNA and plastid trees. An indel in *psbK–psbI* is mapped onto the phylogram.

*Avellinia michelii* is part of Koeleriinae clade A in all trees, and is unique in Koeleriinae by having a 298 bp deletion in the *psbK–psbI* intergenic spacer region. However, affinities of *A. michelii* are discordant in nrDNA and plastid trees. In the ITS+ETS tree, *A. michelii* is the sister group of a Trisetum sect. Trisetaera + *Koeleria* + *Gaudinia* clade. The topology of the more poorly resolved ITS tree in Wölk and Röser (2017) is consistent with this. By contrast, in the combined plastid and *matK* (Suppl. material 7) trees, *A. michelii* is sister to *T. flavescens* + *Rostraria*, consistent with an earlier plastid study in which *Avellinia* falls on a long branch and groups with *Trisetum glaciale*, *T. paniceum*, *T. barengense* and a clade of *T. flavescens*, *T. gracile* and three species of *Rostraria* (*R. litorea*, *R. obtusiflora* (Boiss.) Holub, *R. salzmannii* and *R. pumila*) (Quintanar et al. 2007). The plastid tree is better resolved than the one in Wölk and Röser (2017), in which *Avellinia* forms a polytomy with several other lineages of Koeleriinae clade A.

*Rostraria* is monophyletic in plastid but not nrDNA trees. The two newly sampled accessions of *R. pumila* in the plastid and ITS+ETS trees are sister to *T. flavescens*, and the *matK* tree (Suppl. material 7) includes five species of *Rostraria* (*R. azorica* S. Henderson, *R. cristata*, *R. pumila* and *R. salzmanii*), which form a clade sister to *T. flavescens*. In the ITS tree, however, *R. cristata*, *R. hispida* and *R. obtusiflora* do not form a clade with *R. pumila*, *R. litorea* and *R. salzmanii*, as in Quintanar et al. (2007). *Rostraria cristata*, the accepted name for the lectotype of the genus (*R. pubescens* Trin., *nom. illeg.*), is one of the species with discordant placements in nrDNA and plastid trees, potentially complicating circumscription of the genus. This taxon may have a hybrid origin. Ploidy levels of 2*n*=14, 21 and 28 are recorded for *R. cristata* (Watson and Dallwitz 1992 onwards, Spies et al. 1996b), consistent with a putative hybrid origin of at least some cytotypes. Several species of *Rostraria* have not yet been sampled in phylogenetic work, including *R. balansae* (Coss. & Durieu) Holub (north Africa), *R. berythea* (Boiss. & C.I. Blanche) Holub (western Asia), *R. clarkeana* (Domin) Holub (India) and *R. rohlfsii* (Asch) Holub (north and west tropical Africa). These were originally described as species of *Koeleria*. Clarification of generic circumscription of species of *Rostraria* awaits better taxon sampling of the genus and taxonomic decisions for the whole clade.

**Figure 24.**
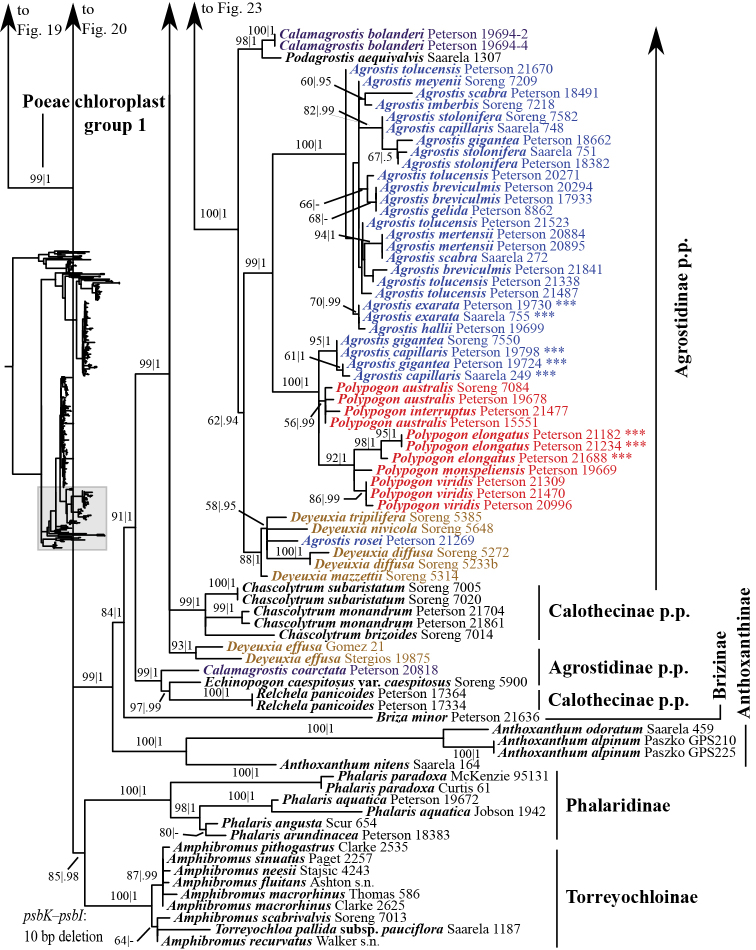
A portion (part of Agrostidinae p.p., Anthoxanthinae, Brizinae, Calothecinae, Phalaridinae and Torreyochloinae) of the maximum likelihood phylogram inferred from combined plastid data (*atpF–atpH*, *psbK–psbI*, *psbA–rps19–trnH*, *matK*, *trnL–trnF*). ML bootstrap support (left) and BI poster probabilities (right) are recorded along branches. A dash indicates posterior probability <.5. No support is shown for branches with bootstrap support <50% and posterior probability <.5. The shaded area of the smaller tree on the left indicates the location in the overall tree of the portion shown. Placements of samples with asterisks (***) are incongruent in nrDNA and plastid trees. An indel in *psbK–psbI* is mapped onto the phylogram.

Affinities of *Gaudinia* reported here in plastid and nrDNA trees are mostly better resolved and supported than in Quintanar et al. (2007). In the ITS+ETS tree, *Gaudinia* is sister to a Trisetum sect. Trisetaera + *Koeleria* clade, and the topology is similar in the plastid tree. In ITS trees here and elsewhere (Quintanar et al. 2007, Wölk and Röser 2017), however, affinities of *Gaudinia* are unsupported. Moreover, in the better sampled *matK* tree (Suppl. material 7), *Gaudinia* is not monophyletic because *Trisetaria loeflingiana* is nested in a clade with three species of *Gaudinia* and sister to *G. hispanica*. *Trisetaria loeflingiana* (not sampled in the main analyses) is similarly closely related to *Gaudinia* in the *trnL–trnF* phylogeny in Quintanar et al. (2007), and the two taxa form a weakly supported clade in the *trnL–trnF* tree here (Suppl. material 11).

Of the 13 species of Trisetum sect. Trisetaera recognized in the New World, most from South America (Finot et al. 2005b, Finot 2010), we sampled *T. andinum* Benth., *T. macbridei*, *T. montanum* Vasey, *T. oreophilum* Louise-Marie, *T. phleoides* (d’Urv.) Kunth, *T. preslii* (Kunth) Hitchc., *T. rosei* Scribn. & Merr. and *T. spicatum*. Except for *T. spicatum*, none of these species has been included in previous phylogenetic studies. The results confirm these taxa are closely related to each other and to the globally widespread *T. spicatum*, consistent with their classification together in Trisetum sect. Trisetaera. However, there is little resolution of relationships among these species in the trees here, and no clades correspond to the three morphologically-defined clusters of species of Trisetum sect. Trisetaera identified by Finot (2010). Placement of *T. montanum*, a species Finot et al. (2005a) included in Trisetum sect. Trisetum on the basis of its lax, open to more or less contracted panicle, among species of Trisetum sect. Trisetaera in plastid and nrDNA trees is unsurprising because *T. montanum* is often treated as a synonym or subspecies of *T. spicatum* (Weber 1976, Rumely 2007). As well, our results suggest *T. macbridei*, endemic to Peru (Finot et al. 2005b), has a hybrid origin between unknown species of Koeleriinae clades A and B, given its placement in either clade in the nrDNA and plastid trees, respectively. In the plastid tree, *T. macbridei* is part of a large clade of South American species *Calamagrostis*/*Deyeuxia*.

Although the species of Trisetum sect. Trisetaera are closely related to one another, the section is paraphyletic with respect to some or all species of *Koeleria*. In the ITS+ETS tree, all species of Trisetum sect. Trisetaera and *Koeleria* form a clade, with little internal structure. In the plastid tree, however, Trisetum sect. Trisetaera and three species of *Koeleria* (*K. macrantha* (Ledeb.) Schult., *K. permollis* Nees ex Steud. and *K. vallesiana* Asch. & Graebn) form a clade, but three other species of *Koeleria* (*K. capensis* (Steud.) Nees, *K. lobata* (Bieb.) R. & S. and *K. splendens* C. Presl) are excluded from the clade and their affinities in Koeleriinae clade A are unresolved. A similar topology is found in the plastid tree in Wölk and Röser (2017), who included some of the same species of *Koeleria*, but also some different ones. These different topologies in nrDNA and plastid trees may be due to ancient hybridization. Additional sampling of *Koeleria* is needed to clarify its evolutionary history, especially since *Koeleria* is the most poorly sampled genus in Koeleriinae. Nevertheless, the molecular data indicate that all taxa in this clade should be recognized in the same genus.

#### *Lagurus*

*Lagurus* includes one annual species endemic to the Mediterranean region and introduced in North and South America, southern Africa and Australia (Clayton and Renvoize 1986, Tucker 2007). *Lagurus ovatus* is diploid (2*n*=14) (Romero Zarco 1988, Spies and Voges 1988, Spies et al. 1996a, Rice et al. 2015) and characterized by having panicles spiciform and ovate, spikelets one-flowered with a rachilla extension, glumes white villous and acuminate or with a slender awn, and lemmas two-awned at the tip with a geniculate dorsal awn (Clayton and Renvoize 1986). Morphology-based classifications included *Lagurus* in Agrostidinae based on its one-flowered spikelets (reviewed in Quintanar et al. 2007). Within Agrostidinae, Clayton and Renvoize (1986) hypothesized a close relationship among *Lagurus*, *Agrostis*, *Triplachne* and *Gastridium*. However, other characteristics of the genus, including a glabrous ovary, a short hilum and liquid endosperm, support a relationship with Koeleriinae (Quintanar et al. 2007), consistent with molecular data.

Molecular analyses place *Lagurus* in Aveninae s.l., but nrDNA and plastid trees are incongruent regarding its affinities in the clade. *Lagurus* is part of the Aveneae lineage in a phylogeny based on combined chloroplast DNA restriction site data and morphology (Soreng and Davis 2000), and is more closely related to *Avena* than *Trisetum spicatum* in a phylogeny based on the 5S nrDNA spacer (Röser et al. 2001). Quintanar et al. (2007) found *Lagurus* to be part of Koeleriinae in their ITS tree, whereas the genus is part of a broader unresolved clade including all taxa of Koeleriinae and Aveninae s.str. in their plastid tree. Other plastid trees identify *Lagurus* as the sister group of a clade including *Arrhenatherum*, *Avena*, *Gaudinia* and *Rostraria* (Grass Phylogeny Working Group II 2012); as part of a polytomy with *Tricholemma jahandiezii* (Litard. ex Jahand. & Maire) Röser, a clade of four *Helictotrichon* species and a clade comprising the rest of Aveninae s.str. and Koeleriinae (Wölk and Röser 2017); and as part of a clade with *Helictotrichon jahandiezii* (Litard. ex Jahand. & Maire) Potztal (=*Tricholemma jahandiezii*) that is sister to a clade including *Arrhenatherum*, *Avena*, *Helictotrichon*, *Koeleria* and *Pseudarrhenatherum* (Schneider et al. 2009). (The latter clade also included a species of *Hierochloe*, likely the result of an identification or laboratory error because this genus is not part of the Aveninae lineage.) In a nuclear *topo6* tree, *L. ovatus* was part of a clade with taxa of Koeleriinae (*Trisetum flavescens*, *Graphephorum* and a *Koeleria* + *Trisetum sect. Trisetaera* clade) (Wölk and Röser 2014), consistent with ITS trees.

Affinities of *Lagurus* in the trees reported here are congruent with these earlier studies. In the ITS tree, *Lagurus* is part of the moderately supported Koeleriinae clade, but is excluded from Koeleriinae clades A and B, whereas in the plastid tree *Lagurus* is sister to a clade comprising the remainder of Aveninae s.str. and Koeleriinae. In the *matK* tree (Suppl. material 7), however, *Tricholemma* (not sampled in the combined plastid tree) and *Lagurus* are successively diverging sisters of a clade comprising the rest of Aveninae s.str. and Koeleriinae, a topology congruent with, and better resolved than, the tree in Wölk and Röser (2014). In both plastid and nrDNA trees, *L. ovatus* falls on long branches, representing an accelerated rate of evolution along the lineage possibly related to its annual habit. The incongruence between nrDNA and plastid trees may indicate a hybrid origin for the genus, or its placement may be a long-branch artefact in one or both analyses. Despite the incongruence, the phylogenetic evidence is consistent with inclusion of *Lagurus* in Aveninae s.l. (Soreng et al. 2015b). In a classification recognizing both Aveninae s.str. and Koeleriinae, however, appropriate placement for *Lagurus* is unclear, because of the incongruence. An alternative solution is to recognize *Lagurus* it in its own subtribe, Lagurinae, here proposed (see Taxonomy), reflecting the apparently unique origins of its plastid and nrDNA, and its unique morphology in the subtribe, particularly its combination of one-flowered spikelets and villous glumes.

#### Koeleriinae Clade B

Compared to a previous study (Saarela et al. 2010), we substantially increased sampling of taxa in Koeleriinae clade B. We newly sampled four (*Trisetum virletii*, *T. viride*, *T. palmeri*, *T. durangense*) of the eight Mexican species of Trisetum subg. Deschampsioidea, five of the six species of *Sphenopholis*, and multiple species of *Calamagrostis*/*Deyeuxia* from Mexico, Central America and South America. Species of Trisetum subg. Deschampsioidea are more closely related to *Peyritschia*, *Graphephorum* and *Trisetum cernuum* (in the plastid tree), *Trisetopsis* and *Calamagrostis*/*Deyeuxia* p.p. than to most species of Trisetum subg. Trisetum. This is consistent with some evidence from morphology. Finot (2006) found that variation in leaf epidermis characteristics distinguishes most species of Trisetum subg. Deschampsioidea from Trisetum subg. Trisetum. He did not, however, compare leaf epidermis morphology of Trisetum subg. Deschampsioidea with any species of *Calamagrostis*/*Deyeuxia* or the other genera of this lineage.

#### *Calamagrostis*/*Deyeuxia*

Our analyses confirm the polyphyly of *Calamagrostis*/*Deyeuxia* as demonstrated previously with ITS, plastid and *topo6* data, although only few species were sampled in earlier studies (Saarela et al. 2010, Wölk and Röser 2014, 2017). In the plastid and nrDNA trees, all but a small subset of species of *Calamagrostis*/*Deyeuxia* from Mexico, Central and South America are part of Koeleriinae clade B (the exceptions are discussed under Agrostidinae and *Deschampsia*). Placement of these species in the Koeleriinae clade, rather than the Agrostidinae clade, indicates they are grossly misclassified. This misclassification was based, in part, on the one-flowered spikelets of these taxa. With the exception of *Lagurus*, species of Koeleriinae (and Aveninae s.l.) as previously understood have two or more flowers in each spikelet. Further work is needed to characterize the evolutionary origins of one vs. two-or-more flowered spikelets within Koeleriinae clade B.

There is some phylogenetic structure within Koeleriinae clade B. All but five of the species of *Calamagrostis*/*Deyeuxia* that are part of the clade form a clade with Trisetum subg. Deschampsioidea and *Leptophyllochloa*. Within this clade, three of the five sampled species of *Calamagrostis*/*Deyeuxia* from Mexico and all sampled species of Trisetum subg. Deschampsioidea form a strongly supported clade in the plastid and nrDNA trees. Two species in this clade, *T. durangense* and *C. divaricata*, were described recently from Durango, Mexico (Finot et al. 2004, Peterson et al. 2004). At the time of its description, *C. divaricata* was known only from its type locality. One sample of this taxon here is from the holotype (*Peterson et al. 17774*) and the other (*P.M. Peterson & J.M. Saarela 21267*, US, CAN-602202) is from a nearby but previously unreported site for the species, ca. 12 km [air] from the holotype collection site. The sites of the two samples of *T. durangense*, both from Durango, are also new records for the species.

Rúgolo de Agrasar (2006) presented a provisional classification of *Deyeuxia* in South America, in which she recognized five sections (including Deyeuxia sect. Stylagrostis, which is discussed below), and the molecular phylogeny provides support for some aspects of this classification. The *Calamagrostis*/*Deyeuxia* + Trisetum subg. Deschampsioidea + *Leptophyllochloa* clade includes a strongly supported lineage of three species (*D. rupestris* (Trin.) Rúgolo, *D. viridiflavescens* (Poir.) Kunth and *D. viridis* Phil.) in nrDNA and plastid trees that corresponds to Deyeuxia sect. Viridiflavescentia Rúgolo & Villav.; three other species included in the section are not sampled here. This section is defined by having calluses recurved, rachillas prolonged beyond the paleas, small anthers, and caryopses with soft endosperm and high lipid content. An unsupported clade in the ITS+ETS tree includes *D. heterophylla* (Wedd.) Pilg., *D. nana* Rúgolo, *D. rigescens* (J. Presl) Türpe (the type) and *D. vicunarum* Wedd., all classified in Deyeuxia sect. Chamaeacalamus (Pilg.) Rúgolo & Villav., as well as *D. coarctata* Kunth (not treated in the classification) and *D. lagurus* Wedd. (Deyeuxia sect. Deyeuxia). A subclade formed by these species but excluding *D. heterophylla* is weakly to strongly supported in the ITS+ETS tree. None of these species form a clade in the plastid tree. Deyeuxia sect. Chamaecalamus includes about ten high Andean species distinguished by having lemmas with four aristate or deltoid teeth, cleistogamous flowers, anthers 0.3–0.6 mm long and generally adhered to the apex of the fruit, rachillas 0.3–2.2 mm long and glabrous or scarcely hairy, and caryopses fusiform (Rúgolo de Agrasar 2006). An unsupported to weakly supported clade in the ITS+ETS tree includes most sampled species of *Deyeuxia* sects. *Deyeuxia* and *Pungentes* Rúgolo. However, *Deyeuxia rigida* and *D. recta*, included in “Grupo Rigida” of Deyeuxia sect. Deyeuxia, are not part of this clade; instead, they form a clade with *Peyritschia deyeuxioides* and three other species of *Deyeuxia* not treated in the classification. Limited molecular variation among many of the sampled *Calamagrostis*/*Deyeuxia* taxa that are part of Koeleriinae clade B may be the result of a rapid radiation in South America.

#### *Leptophyllochloa*

*Leptophyllochloa* is a monotypic genus from the southern Andean ranges (Quintanar et al. 2010) whose affinities have been uncertain. *Leptophyllochloa micranthera* (E. Desv.) C.E. Calderon was initially described as *Trisetum macratherum* E. Desv. and has also been treated as *Koeleria micranthera* (E. Desv.) Griseb. (Clayton and Renvoize 1986, Watson and Dallwitz 1992 onwards). The taxon is characterized by having loose panicles, three-nerved lemmas and subapically inserted short-awned lemmas. *Leptophyllochloa* was sampled for the first time in a recent molecular study (Wölk and Röser 2017); in their plastid tree it was placed in Poeae chloroplast group 2, and in their ITS tree it was sister to a strongly supported clade comprising *Avenula* + *Parvotrisetum* Chrtek, Torreyochloinae + Agrostidinae + Calothecinae and Aveninae s.l. By contrast, we find *Leptophyllochloa* to be part of Koeleriinae clade B in both nrDNA and plastid trees, consistent with the morphology-based classifications that have considered it to be closely related to other species of Koeleriinae. Although its precise affinities in the clade are unresolved, the results do not support treating the taxon in *Koeleria* (Clayton and Renvoize 1986, Watson and Dallwitz 1992 onwards), whose species are part of Koeleriinae clade A. Given the substantial differences in placement of the single samples of *Leptophyllochloa* in the current study and in Wölk and Röser (2017), we suspect one of the placements for the genus is an error. Further samples of *Leptophyllochloa* are needed to confirm its phylogenetic affinities.

#### *Peyritschia*

*Peyritschia* Fournier (1886) was based on a single species, *P. koelerioides* (Peyr.) E. Fourn., from southern Mexico and Guatemala originally described as *Aira koelerioides* Peyr. (Finot et al. 2004). The genus has also been treated as a synonym of *Deschampsia* and *Trisetum* (Koch 1979, Hernandez Torres and Koch 1988). A second species, *P. pringlei* (Scribn.) S.D. Koch, distributed from Mexico to Venezuela and Ecuador, was transferred from *Deschampsia* to *Peyritschia* by Koch (1979). More recently, five species of *Trisetum* from Mexico, Central and South America were transferred to *Peyritschia* (*P. conferta* (Pilg.) Finot, *P. deyeuxioides*, *P. humilis* (Louis-Marie) Finot, *P. howellii* (Hitchc.) Finot & P.M. Peterson, *P. pinetorum* (Swallen) Finot & P.M. Peterson) (Finot 2003, Finot et al. 2006b), bringing the number of species currently recognized in the genus to seven. *Peyritschia koelerioides*, *P. deyeuxiodes* and *P. pringlei* are tetraploids (2*n*=28) (Pohl and Davidse 1971, Koch 1979). We are not aware of chromosome counts for the other four species. *Peyritschia* differs from *Trisetum* by having one-nerved glumes, bilobed lemmas awned from near the base or from the middle of the back or awns reduced to a subapical mucro, paleas tightly enclosed by the margins of the lemma and an androecium of two stamens (Torres and Koch 1988, Finot et al. 2006b). It also differs from *Trisetum* in multiple lemma epidermal characteristics: the four species of *Peyritschia* studied by Finot et al. (2006a) lack silica cells, macrohairs and prickle hairs on the lemma epidermis, whereas these characters generally are present in species of Trisetum subg. Trisetum and Trisetum subg. Deschampsioidea. Leaf epidermis characteristics, however, do not distinguish *Peyritschia* and *Trisetum* (Finot 2006).

*Peyritschia* has been poorly sampled in molecular phylogenies. In a previous study, one individual each of *P. pringlei* and *P. deyeuxioides* was sampled, and in plastid and nrDNA trees these were intermixed with species of *Calamagrostis*, *Sphenopholis* and *Trisetum* (Saarela et al. 2010). *Peyritschia pringlei*, *P. deyeuxiodes* and *P. koelerioides* were variously sampled in other recent studies, and they form a clade of unresolved affinity in Koeleriinae clade B (Wölk and Röser 2014, 2017). The ITS+ETS and plastid trees reported here only include *P. deyeuxioides*. It is not closely related to any of the Mexican species of *Trisetum*, consistent with the lemma epidermal data, but forms a weakly supported clade with five species of *Calamagrostis*/*Deyeuxia* from Central to South America. Given the poor taxon sampling here and elsewhere (less than half the species of the genus have been sampled) the monophyly of *Peyritschia* in its current circumscription remains unconfirmed.

#### *Sphenopholis*

*Sphenopholis* (type *S. obtusata*) is a small genus of six to seven perennial species endemic to North and Central America (Finot et al. 2004; Daniel 2007). The genus is characterized by having spikelets that disarticulate below the glumes and between florets, upper glumes oblanceolate to obovate (the glumes are strongly dimorphic), and lemmas with entire or two-toothed apices and awnless or awned just below the apex (Finot et al. 2004). All species of *Sphenopholis* are diploid (Erdman 1965). A detailed taxonomic history of the genus is given in Erdman (1965). Although some species recognized in *Sphenopholis* have been included in *Trisetum* (e.g., Trisetum subg. Colobanthus (Trin.) Rchb.) (Trinius 1830, Reichenbach 1841, Hitchcock 1951), the genus is recognized widely in the floristic literature.

Few species of *Sphenopholis* have been studied phylogenetically. Only *S. intermedia* (Rydb.) Rydb. was included in Quintanar et al. (2007). In their ITS tree, it formed a polytomy with *Lagurus ovatus* and a clade comprising species of *Gaudinia*, *Graphephorum*, *Koeleria*, *Rostraria* and *Trisetum*. In their plastid tree, *S. intermedia* and *Graphephorum wolfii* formed a clade that was part of a polytomy with two other lineages formed by species of *Koeleria*, *Trisetum*, *Gaudinia* and *Avellinia* (these are part of Koeleriinae clade A here). In a previous ITS tree, *S. intermedia* was part of a lineage including species of Koeleriinae clade B (Saarela et al. 2010). In a nuclear multi-gene study (Hochbach et al. 2015), *S. obtusata* was closely related to *Limnodea arkansana* (Benth.) L.H. Dewey, a monotypic genus from the southeastern United States and adjacent Mexico now included in Agrostidinae, and these two species were part of a clade with *Gaudinia* and *Koeleria*. (*Limnodea* is sampled here only in the *matK* tree (Suppl. material 7). It is part of Koeleriinae clade B, but its affinities with *Sphenopholis* and all other taxa in the clade are unresolved.) Wölk and Röser (2014, 2017) included four species of *Sphenopholis* in their analyses; the genus was resolved as part of Koeleriinae clade B in all their trees, but was recovered as monophyletic only in their *topo6* trees.

We analyzed four species of *Sphenopholis*. The genus is part of Koeleriinae clade B and is recovered as monophyletic in the ITS+ETS and plastid trees, with strong support in both. *Sphenopholis filiformis* Trin., distributed across the southeastern United States, is sister to the rest of the genus in the ITS+ETS tree, but not in the plastid tree. *Sphenopholis longiflora* (Vasey ex L.H. Dewey) Hitchc. (Texas, Arkansas and Louisiana) and *S. interrupta* (Buckley) Scribn. (southern U.S.A. and Mexico) are the only species of the genus not yet sampled in a molecular study. *Sphenopholis interrupta* should be a priority for future sampling because the taxon has been treated in both *Sphenopholis* (Scribner 1906, Finot et al. 2004) and *Trisetum* (as *T. interruptum* Buckley) (Rumely 2007). Placement of this species in *Sphenopholis* is supported by epidermis micromorphological characters (Finot et al. 2006a).

#### *Trisetopsis*

*Trisetopsis* is a recently described genus of ca. 24 species distributed in tropical and subtropical Africa, Madagascar and the Arabian Peninsula (Wölk and Röser 2013). The few species for which chromosome numbers are known are polyploid (Wölk and Röser 2014). All species were previously recognized in *Helictotrichon* (Schweickerdt 1937, Mashau et al. 2010), and some were initially described as species of *Trisetum* (Wölk and Röser 2013). *Trisetopsis* is distinguished from *Helictotrichon* s.str. by having deeply bifid lemmas usually extending to the insertion of the awn, ovaries sparsely ciliate apically, and lodicules narrowly to broadly ovate and apically narrowed or bi- to trifid (Wölk and Röser 2013, 2014). ITS, plastid and *topo6* trees all place *Trisetopsis* in Koeleriinae clade B, and *topo6* data indicate an allopolyploid origin for the genus. Wölk and Röser (2014) identified two copy types (A and B) of *topo6* in *Trisetopsis*. Copy type A formed a strongly supported lineage with the more distantly-related species *Arrhenatherum elatius* (Aveninae s.str.), whereas copy type B was part of a lineage with species of *Calamagrostis*, *Peyritschia*, *Sphenopholis* and two cloned sequences of *Graphephorum melicoides*. Copy type B likely originated from New World taxa or the common ancestor of these taxa and *Trisetopsis*. Wölk and Röser (2014) did not include any species of Trisetum subg. Deschampsioidea in their study. We did not newly sample any species of *Trisetopsis*, but included the published ITS sequences in the analysis. *Trisetopsis* is recovered as a weakly supported clade in Koeleriinae clade B in the ITS tree, but its precise affinities are unresolved. The *matK* tree (Suppl. material 7) includes one species of *Trisetopsis*, which is part of Koeleriinae clade B, and its affinities with other taxa in the clade are unresolved. Morphological characters supporting the molecular placement of *Trisetopsis* in Koeleriinae have not yet been characterized (Wölk and Röser 2014).

#### Generic classification in Koeleriinae

Given the current phylogenetic evidence, substantial generic re-circumscriptions will likely be necessary for a natural classification of the Koeleriinae. None of the recognized genera are monophyletic in plastid or nrDNA trees, except *Sphenopholis*. Recognition of multiple, narrowly circumscribed genera in Koeleriinae may be complicated by putative reticulation in the origins of some taxa, both within Koeleriinae clade A (e.g., *Avellinia*, *Gaudinia*, *Rostraria* p.p., *Trisetaria* p.p.) and between Koeleriinae clades A and B (e.g., *Graphephorum wolfii*, *Trisetum cernuum*, *T. irazuense*, *T. macbridei*). Incongruence between plastid and nrDNA may also be present in taxa not yet included in molecular phylogenies. The previously-published evidence from a low copy nuclear gene supporting a putative allopolyploid origin for *Trisetopsis*, possibly involving a parental taxon related to *Arrhenatherum*, must also be taken into account for classification. Indeed, all or a subset of other Koeleriinae may have similar origins. Further study of low copy nuclear genes in the subtribe is likely to be insightful in this regard.

One possible solution to the problem of generic classification in Koeleriinae has already been proposed. Kellogg (2015) treated *Avellinia*, *Gaudinia*, *Koeleria*, *Leptophyllochloa*, *Peyritschia*, *Rostraria* and *Trisetum* as synonyms of *Trisetaria*, the genus name with priority, and kept *Graphephorum*, *Lagurus* and *Sphenopholis* separate; she did not, however, propose the many needed new combinations in *Trisetaria*. Kellogg (2015) alternatively suggested the name *Trisetum* could be conserved against *Trisetaria*, and all species recognized in *Trisetum*. This was proposed by Quintanar and Castroviejo (2010), but the proposal was rejected by the Nomenclature Committee for Vascular Plants (Applequist 2017).

An alternative solution to classification may be to recognize Koeleriinae clades A and B and Trisetum subsect. Sibirica as separate genera (Kellogg 2015), although the lineages have not yet been characterized morphologically. Under this scenario, it would be appropriate to continue to recognize *Graphephorum* (including *Trisetum cernuum* and perhaps other closely related species) as a putative hybrid genus between taxa of the two main clades. The name with priority in Koeleriinae clade A is *Trisetaria*. The name with priority in Koeleriinae clade B is *Cinnagrostis* Grisebach (1874), if the type species, *Cinnagrostis polygama* Griseb. (=*Calamagrostis polygama* (Griseb.) Parodi), from western and southern South America (Clayton et al. 2006 onwards), is found to be part of Koeleriinae clade B like most other South American species of *Calamagrostis*/*Deyeuxia*, and as long as *Graphephorum*, the oldest generic name available, is excluded from the genus. Because transferring all species of Koeleriinae clade B to *Cinnagrostis* would necessitate many new combinations, conservation of another available and more-widely used name over *Cinnagrostis* would be an option. This would not, however, greatly minimize the number of combinations needed, because the available generic names in the clade (e.g., *Peyritschia*, *Sphenopholis*) each have few existing relevant combinations. Yet another option may be to propose conservation of the name *Deyeuxia* with a type that is part of Koeleriinae clade B. This option would minimize the number of new combinations needed because the majority of taxa in the clade already have names in *Deyeuxia*. Whatever the nomenclatural solution at the genus level, developing a natural classification below the rank of genus will be as complicated as trying to circumscribe numerous genera because the same patterns of reticulation will have to be dealt with, and because many aspects of relationships in each major clade are as yet unresolved.

#### Agrostidinae + Calothecinae + Brizinae

Our phylogenetic analyses identify a large clade that includes taxa of Agrostidinae, Calothecinae and Brizinae, but neither Agrostidinae nor Calothecinae are monophyletic. The clade is moderately to strongly supported in the plastid tree and weakly supported in the ITS+ETS tree. A similar, poorly supported clade was identified in an earlier study with poorer taxon and gene sampling (Saarela et al. 2010). Persson and Rydin (2016) also identified a similar but poorly supported clade, also including Anthoxanthinae, in their ITS+GBSSI tree. The Agrostidinae + Calothecinae + Brizinae clade here is further defined by having one large insertion and one large deletion in the ETS region. Both indels are present in all taxa in the clade. The insertion is also present in the distantly related Anthoxanthinae, perhaps reflecting homoplasy. Three main lineages are identified in this large clade: (1) *Chascolytrum* (Calothecinae p.p.) and *Deyeuxia effusa* (Agrostidinae p.p.); (2) Agrostidinae p.p.; (3) Brizinae; and (4) *Relchela* (Calothecinae p.p.), *Echinopogon*, *Calamagrostis coarctata* and *Dichelachne* (Agrostidinae p.p.); *Dichelachne* is sampled only in the ITS and *matK* (Suppl. material 7) trees. Of these lineages, *Chascolytrum* + *D. effusa*, Agrostidinae p.p. and Brizinae form a weakly supported clade in the ITS+ETS tree and *Chascolytrum* + *D. effusa* and Agrostidinae p.p. form a moderately to strongly supported clade in the plastid tree, the strongest obtained to date for the lineage. Kellogg (2015) included *Briza*, *Chascolytrum* and *Relchela* in a more broadly circumscribed Agrostidinae, defined by having lemmas awnless or with an abaxial awn, the awn often geniculate, and paleas generally hyaline with the margins often wider than the space between the veins. Such a circumscription of Agrostidinae is consistent with the current phylogenetic evidence.

#### *Chascolytrum* (Calothecinae p.p.) + *Deyeuxia effusa* (Agrostidinae p.p.)

Calothecinae in its current circumscription, including *Chascolytrum* and *Relchela*, is not monophyletic. Previous studies have, however, found *Chascolytrum* s.l. to be monophyletic (Essi et al. 2008, Persson and Rydin 2016), as we also find. The species of *Chascolytrum* we sampled form a clade in the ITS+ETS and plastid trees, and all species of *Chascolytrum* in the ITS tree form a weakly supported clade. A novel result here, however, is a strongly supported relationship between *Chascolytrum* and the western and northern South American species *Deyeuxia effusa* (=*Calamagrostis effusa* (Kunth) Steud.) in the ITS+ETS tree. In the plastid tree, *D. effusa* and *Chascolytrum* are not resolved in a clade, and these plus Agrostidinae p.p. form a polytomy within a strongly supported clade. Further study is needed to identify putative morphological similarities between *D. effusa* and *Chascolytrum*, and to determine if other species of *Calamagrostis*/*Deyeuxia* are allied with *Chascolytrum*.

#### *Relchela* (Calothecinae p.p.) + *Echinopogon* + *Calamagrostis coarctata* + *Dichelachne* (Agrostidinae p.p.)

The three taxon clade including *Relchela* and a subclade comprising *Echinopogon caespitosus* and *Calamagrostis coarctata* is weakly supported in the ITS+ETS tree and strongly supported in the plastid tree. None of these taxa have previously been thought to be closely related to one another. The monotypic *Relchela* (*R. panicoides*), distributed in Argentina and Chile, is characterized by having a perennial habit, panicles contracted, spikelets one- to two-flowered with or without a rachilla extension, glumes longer than the hard lemma(s), callus pubescent and ovary apex hairy (Clayton and Renvoize 1986, Kellogg 2015). *Relchela panicoides* has been variously classified in Agrostidinae and treated as a species of *Agrostis* or *Calamagrostis* (Muñoz 1941), in Brizinae as *Relchela* (Soreng et al. 2007) or *Briza* (in Briza sect. Relchela (Steud.) Pilg.) (Björkman 1960), in Aveninae (Clayton and Renvoize 1986, Soreng et al. 2003) and in Calothecinae (Soreng et al. 2015b). None of these classifications is consistent with molecular phylogenetic data. *Relchela* has only been sampled previously in two studies. In an ITS tree, it is part of a clade of Agrostidinae taxa (Refulio-Rodriguez 2007), generally congruent with the current results. In the plastid tree in Wölk and Röser (2017), *Relchela* is sister to a clade including *Briza media*, *Agrostis* and *Calamagrostis*, similar to the trees here but with the branching order of *Relchela* and *Briza* flipped. In the ITS tree in Wölk and Röser (2017), *Relchela* is sister to *Agrostis* and *Calamagrostis*, whereas *B. media* forms a clade with Anthoxanthinae.

Placement of *Calamagrostis coarctata* in a clade with *Echinopogon* and *Relchela* was unexpected. *Calamagrostis coarctata* [=*C. cinnoides* (Muhl.) W.P.C. Barton, *nom. illeg.*, as treated in Marr et al. (2007)] is an eastern North American species not previously included in a phylogenetic analysis. The taxon was considered unusual in its genus over a century ago. Kearney (1898) noted *C. coarctata* to be “an extremely isolated type without near relations” differing from other North American species of *Calamagrostis* by having a rachilla prolongation naked until just below the apex where there is a circle of long hairs (vs. rachilla prolongation villous along its whole length to just below the apex) and a hairy ovary. *Relchela* also has an apically pubescent ovary, which may be morphological synapomorphy supporting the close relationship between these taxa. Additional sampling of *C. coarctata* is needed to confirm its placement, since we sampled only one accession of the taxon.

In previous studies, *Echinopogon* has been placed in a clade with other species of Agrostidinae, and a close relationship between *Echinopogon* and *Dichelachne* (not sampled in the main analyses) has also been found. *Echinopogon* is a genus of seven perennial polyploid (2*n*=42) species from New Guinea, Australia and New Zealand characterized by having panicles spiciform to capitate, spikelets one-flowered with a rachilla extension, lemmas 5–11-nerved with a stiff terminal or subapical awn and calluses shortly bearded (Clayton and Renvoize 1986, Kellogg 2015). *Dichelachne* is a genus of five polyploid (2*n*=70) species also native to new Guinea, Australia and New Zealand characterized by having a perennial habit, panicles contracted, spikelets one-flowered with or without minute rachilla extension, lemmas with a long wavy dorsal to subapical awn two to six times its length and calluses pubescent (Clayton and Renvoize 1986, Kellogg 2015). Veldkamp (1974) considered *Dichelachne* to be closely related to *Deyeuxia*, and Kellogg (2015) included *Dichelachne* in *Deyeuxia*.

In a previous plastid analysis, two species of *Echinopogon* resolved as part of subtribe Agrostidinae and the genus was paraphyletic with respect to *Dichelachne* (Grass Phylogeny Working Group II 2012). In a previous ITS analysis, one accession of *Echinopogon* sp. resolved in a clade with *Briza media*, *Dichelachne* sp., *Calamagrostis purpurascens* and *Agrostis capillaris* (Schneider et al. 2009). In the ITS tree reported here, the same *Echinopogon* accession is part of a clade with a new sample for *E. caespitosus*. Soreng et al. (2007) found a strongly supported *Echinopogon* + *Dichelachne* clade. In the *matK* tree here, *Echinopogon* (two species), *Dichelachne* (at least three species), *Relchela* and *Calamagrostis coarctata* do not form a clade, but are excluded from the main Agrostidinae p.p. clade (Suppl. material 7). In the ITS tree, a strongly supported clade includes *Dichelachne* and two species of *Deyeuxia* (*D. lacustris* Edgar & Connor and *D. quadriseta* (Labill.) Benth.) from Australia and New Zealand. A sample determined as *E. ovatus* (G. Forst.) P. Beauv. in Persson and Rydin (2016), however, is sister to *Desmazeria* Dumort. (Parapholiinae) in their nuclear and plastid trees, while a sample of *E. caespitosus* is part of the Agrostidinae + Calothecinae clade. The *E. ovatus* data in Persson and Rydin (2016) are likely erroneous because other plastid data for this taxon place it among taxa of Agrostidinae (Grass Phylogeny Working Group II 2012). Further morphological and molecular study of all taxa in this small clade is warranted.

#### Brizinae

Brizinae includes *Airopsis* and *Briza* (Soreng et al. 2015b). *Airopsis* (*A. tenella* Coss. & Durand.) is a monotypic Mediterranean genus characterized by having an annual habit, spikelets two-flowered and nearly spherical in outline, and lemmas orbicular without awns (Clayton and Renvoize 1986). In earlier classifications, *Airopsis* was variously included in Aveneae, Aveninae and Airinae (Quintanar et al. 2007). *Briza* includes three to five species of annuals and perennials native to Eurasia with spikelets 3–12(–15)-flowered and oval to elliptic in outline, and lemmas inflated without awns (Kellogg 2015). Quintanar et al. (2007) found *A. tenella* to be allied with *B. media* L. in plastid analyses, as in the *trnL–F* tree reported here (Suppl. material 11), whereas affinities of *A. tenella* relative to *Briza* and taxa of Agrostidinae in their ITS analyses were unresolved, as in the ITS tree here. (The ITS and *trnL–trnF* sequences of *A. tenella* in the trees here are the ones reported in Quintanar et al. (2007)). In the ITS tree, *A. tenella* and species of *Briza* all fall on long branches relative to other taxa in the tree. We did not sample *A. tenella* in the main plastid and nrDNA analyses. Further characterization of the placement of *Airopsis*, particularly in nuclear trees, is needed.

Of the species of *Briza*, we newly sampled only *B. minor*. In the ITS+ETS tree, *B. minor* is a poorly supported sister to the *Calamagrostis coarctata + Echinopogon* + *Relchela* clade. In the nuclear tree (ITS and GBSSI) in Persson and Rydin (2016), *Briza* s.str. (they included in *Briza* s.l. many species now treated in *Chascolytrum*) is weakly supported as sister to Anthoxanthinae, a topology different from that in the nrDNA trees and one not identified elsewhere. In the plastid tree here, *B. minor* is strongly supported as the sister group of the broad Agrostidinae + Calothecinae + *Deyeuxia effusa* clade, a topology that differs from the ITS+ETS tree. The plastid tree is congruent with the one in Quintanar et al. (2007), in which Brizinae are strongly supported as sister to Agrostidinae (Calothecinae not sampled there), and with the plastome tree in Saarela et al. (2015), in which *Briza* and Agrostidinae are sister taxa (although taxon sampling in the plastome study is comparatively sparse). The ITS and *matK* (Suppl. material 7) trees have increased sampling of *Briza* s.str. species, and phylogenetic affinities of these species in these trees are similar to the ITS+ETS and plastid trees, but with poorer support. The different placements of *Briza* in the nrDNA and plastid trees may be an artifact related to the long branch in the former, or may reflect ancient hybridization in the origin of the genus.

The increased taxon sampling of *Briza* in the ITS tree is sufficient to demonstrate that the subdivision of *Briza* is consistent with phylogeny. Three sections of *Briza* are recognized. Tzvelev (1976) treated *B. marcowiczii* Woronow, *B. media* L. and *B. elatior* Sibth. & Sm. in Briza sect. Briza, and *B. minor* in the monotypic Briza sect. Brizella Tzvelev. These four species are sampled in the ITS tree, in which Briza sect. Briza is monophyletic, and the multiple accessions of *B. minor* are its sister group. Multiple samples of *B. media* and *B. minor* are included in the *matK* tree (Suppl. material 7), and they are sister taxa, with the exception of one sequence of *B. minor* (KJ529358) whose placement basal to other samples is the result of some missing data. Persson and Rydin (2016) found the same topology for these species of *Briza* in nuclear (ITS and GBSSI) and plastid trees. They also identified a putative case of plastid introgression or hybridization involving a species of *Calamagrostis* (or a close relative) in one individual of *B. minor*; this requires confirmation.

*Briza maxima* L., native to the Mediterranean and cultivated ornamentally (Snow 2007), has been recognized in its own section (Briza sect. Macrobriza Tzvelev) and genus (*Macrobriza* Tzvelev). *Briza maxima* differs from *B. media* and *B. minor* by having panicles raceme-like with 3–8 spikelets (vs. not raceme-like with numerous spikelets) and lemmas 7–10 mm long (vs. 2.5–8 mm long) (Tzvelev 1976). There is discordance between nrDNA and plastid DNA for *B. maxima*. In the ITS tree, two independently published sequences (Essi et al. 2008, Birch et al. 2014) from *B. maxima* are weakly supported as part of the Aveninae clade; the taxon falls on a long branch and its affinity with other Aveninae lineages is unclear. An ETS sequence of *B. maxima* reported by Birch et al. (2014), from the same specimen as their ITS sequence, is 99% identical to an ETS sequence from *Lachnagrostis adamsonii*, and is probably an error (data not shown). The placement of *B. maxima* in a clade with *B. media* and *B. minor* in the nuclear tree (ITS, GBSSI) in Essi et al. (2008) is discordant with the topology in the ITS tree here. This may be an artifact of sampling because Essi et al. (2008) only sampled species of *Briza* s.str. and *Chascolytrum* s.l., or the placement of *B. maxima* with the other *Briza* taxa may be due to stronger signal in the GBSSI data compared to ITS. In contrast to the nrDNA trees, *matK* sequences of *B. maxima* form a strongly supported clade with the other species of *Briza*, *B. maxima* and *B. minor* + *B. media* are weakly supported sisters (Suppl. material 7). This strong incongruence suggests a possible hybrid origin for *B. maxima* involving a species of *Briza* (the maternal parent) and an unknown taxon (the paternal parent). Persson and Rydin (2016) found the same conflict for *B. maxima* between nuclear (ITS and GBSSI) and plastid data with an increased sampling of individuals, but they did not consider the possibility of a hybrid origin for the taxon. A different flavonoid pattern documented in *B. maxima* compared to *B. minor* and *B. media* (Williams and Murray 1972) may be due to its putative hybrid origin. Given the topologies of nuclear and plastid trees, treatment of *B. maxima* as either a section of *Briza* or its own genus is consistent with the phylogenetic evidence.

An Asian species variously recognized as *Briza humilis* M. Bieb or *Brizochloa humilis* (M. Bieb.) Chrtek & Hadač (Tzvelev 1999, Valdés and Scholz 2006, Hoffmann et al. 2013) is part of a clade including subtribe Poinae in the ITS tree (Suppl. material 4), as in Hoffmann et al. (2013), and is not closely related to *Briza*. Soreng et al. (2015b) recognized *Brizochloa* as a distinct genus and included it in Poineae. Persson and Rydin (2016) increased sampling of *Briza humilis* and similarly found it to be allied with taxa of Poinae in nuclear and plastid trees, confirming this taxon is more appropriately classified in the genus *Brizochloa. Brizochloa* differs from *Briza* in the shape of its lemma (Jirásek and Chrtek 1967).

#### Agrostidinae p.p.

The ITS+ETS and plastid trees reported here include the broadest sampling thus far for Agrostidinae, and identify a major clade that includes most genera currently classified in the subtribe, including *Agrostis*, *Ammophila*, *Calamagrostis*/*Deyeuxia* p.p., *Lachnagrostis*, *Podagrostis* and *Polypogon*. Support for the clade is weak in the ITS+ETS tree, but strong in the plastid tree. This strong support from plastid data is an improvement compared to the plastid tree in Saarela et al. (2010).

In the next sections, we review the taxonomy and phylogenetic data for genera of Agrostidinae based on the current taxon sampling.

#### *Agrostis*, *Chaetopogon*, *Lachnagrostis* and *Polypogon*

*Agrostis* (conserved type *A. canina* L.) includes ca. 220 species distributed globally in temperate regions and on tropical mountains (Clayton and Renvoize 1986). The genus is cytologically diverse, ranging from diploid (2*n*=14) to decaploid (2*n*=70) (Björkman 1960). Previous classifications of *Agrostis* have been mostly regional in nature, and we are not aware of any worldwide synthetic classification of the genus. Subdivision of *Agrostis* is reviewed in Björkman (1960) and Romero Garcia et al. (1988). Because we compare morphology-based classifications of *Agrostis* to the molecular phylogenies we generated, which has not been done in previous studies, we present a brief review of the classification history of the genus.

The main morphological character informing classification in *Agrostis* is the length of the palea relative to the length of the lemma. Species with short paleas or paleas lacking have been placed in Agrostis sect. Agrostis (=Agrostis sect. Trichodium (Michx.) Trin.), and those with long paleas in Agrostis sect. Vilfa (Adans.) Roem. & Schult (lectotype *Vilfa stolonifera* (L.) P. Beauv. =*A. stolonifera*) (Widén 1971). Björkman (1960) considered variation in lemma epidermal morphology to also be important in the subdivision of *Agrostis*, although he did not propose an explicit classification of the 118 species he studied. Björkman (1960) used the term “Trichodium net”, based on unpublished observations of the Swedish scientist T. Vestergren, to describe lemma epidermises in *Agrostis* bearing a fine-meshed network when observed under high magnification. This morphology was referred to as a “Trichodium net” because of its presence primarily in species of Agrostis sect. Trichodium (=Agrostis sect. Agrostis).

Björkman (1960) recognized four groups in *Agrostis* differing in lemma epidermal morphology: (1) lemmatal network (Trichodium net) present on lemma (in 91 species); (2) lemmatal network fragmentarily developed (in nine species); (3) tendency towards lemmatal network [in five species]; (4) lemmatal network wanting (in 13 species). All but one (78 of 79) species with short paleas (ca. ≤ 1/3 the lemma length) had a Trichodium net (group 1), while 15 species with long paleas (ca. >1/3 the lemma length) also had a Trichodium net (group 1). Of the latter, 11 were species from the mountains of tropical Africa. Group 2 included *A. stolonifera*, a species with variable lemma epidermal morphology, and Group 3 included *A. gigantea*. Group 4 included some Australian taxa commonly recognized in *Lachnagrostis* (e.g., Widén 1971, Jacobs 2001), two Mediterranean annuals, including *A. truncatula* Parl., recently recognized as *Neoschischkinia truncatula* (Parl.) Valdés & H. Scholz (Valdés and Scholz 2006), and a few other species. Widén (1971) refined this classification and recognized seven types of epidermal surface structure, but maintained the traditional classification of the genus, placing species in *Agrostis* sects. *Agrostis* and *Vilfa*. Most species placed by Widén (1971) in Agrostis sect. Vilfa, with longer paleas, had fragmentary Trichodium nets or lacked them entirely. More recently, Finot et al. (2011b) studied the micromorphology of lemmas in species of *Agrostis* and *Polypogon* in Chile and came to similar conclusions.

Taxonomy in *Agrostis* is complicated by hybridization among species of *Agrostis* sects. *Agrostis* and *Vilfa*, and some hybrids are fertile (Widén 1971, Belanger et al. 2003a, Watrud et al. 2004). Examples of intersectional hybrids include *A. stolonifera* × *A. mertensii* (Widén 1971) and *A. canina* × *A. stolonifera* (Belanger et al. 2003a), the latter being a cross between the type species of the two main sections of *Agrostis*.

Cytological and molecular research in *Agrostis* has focused on the biology, evolutionary history and breeding of five commercially important species of *Agrostis* used for turf, pasture and erosion control: *A. stolonifera* (creeping bentgrass, 2*n*=4*x*=28, genome constitution A_2_A_2_A_3_A_3_), *A. capillaris* (colonial bentgrass, 2*n*=4*x*=28, A_1_A_1_A_2_A_2_), *A. canina* L. (velvet bentgrass, 2*n*=2*x*=14, A_1_A_1_ or A_2_A_2_), *A. castellana* Boiss. & Reut. (dryland bentgrass, 2*n*=6*x*=42, A_1_A_1_A_2_A_2_) and *A. gigantea* (redtop bentgrass, 2*n*=6*x*=42, A_1_A_1_A_2_A_2_A_3_A_3_) (Jones 1956a, c, b, Rotter et al. 2007, 2010, Honig et al. 2015). Characterizing relationships among these species has been challenging because most are allopolyploids. A genus-wide phylogeny would be useful to turfgrass researchers because it would provide a broad evolutionary context for the genus, which is currently lacking, and may contribute to identifying the diploids that hybridized and formed the commercially important polyploid species of *Agrostis* (Rotter et al. 2010). Aside from *A. canina* and *A. transcaspica*, none of the few known diploid species of *Agrostis* (e.g., *A. alpina* Scop., *A. atlantica* Maire & Trab., *A. curtisii* Kerguélen, *A. delicatula* Pourr. ex Lapeyr., *A. pourretii* Willd., *A. reuteri* Boiss., *A. rosei*, *A. tenerrima* Trin.) (Rice et al. 2015) have been sampled in molecular studies. Of these, only *A. rosei* is newly sampled here.

One or a few species of *Agrostis* have been included in broader phylogenetic studies of grasses, but there have been few studies with broad sampling of the genus overall. Most phylogenetic studies of *Agrostis* have been focused on better understanding the commercial species, but none discussed their results in the context of subgeneric classification of the genus. Some of these studies demonstrated a close relationship between *Agrostis* and *Polypogon*. Reichman et al. (2006) generated ITS and *matK* phylogenies for *Agrostis*, in a study focused on identifying transgenic individuals of *A. stolonifera*. Saarela et al. (2010) included a few species of *Agrostis*, but did not comment in detail on their placement in phylogenetic trees. Rotter et al. (2010) constructed ITS and plastid phylogenies for creeping, colonial and velvet bentgrasses and a few other species of *Agrostis* and *Polypogon*. Their ITS tree identified a strongly supported clade comprising *A. exarata*, *A. truncatula* Trin., *Polypogon viridis* and *P. monspeliensis*, and a moderately supported clade including two subclades, one of *A. capillaris*, *A. gigantea* and *A. castellana*, and the other of 14 species (*A. canina*, *A. idahoensis* Nash, *A. imbecilla* Zotov, *A. magellanica* Lam., *A. mertensii* Trin., *A. muelleriana* Vickery, *A. muscosa* Kirk, *A. pallens* Trin., *A. pallescens* Cheeseman, *A. personata* Edgar, *A. petriei* Hack., *A. scabra* Willd., *A. stolonifera* and *A. vinealis* Schreb.). Their plastid tree had a different topology with two main clades, one including *A. canina*, *A. exarata*, *A. idahoensis*, *A. mertensii*, *A. pallens*, *A. stolonifera* and *A. vinealis*, and the other including *A. capillaris*, *A. castellana*, *A. gigantea*, *Polypogon monspeliensis* and *P. viridis*. They concluded the diploid *A. canina* is the maternal parent of the allopolyploid *A. stolonifera* and suggested changing its genome formula from A_1_A_1_ to A_2_A_2_, in contradiction to the cytological work of K. Jones, who did not hypothesize a shared genome between *A. canina* (A_1_A_1_) and *A. stolonifera* (A_2_A_2_A_3_A_3_). In a phenetic study based on nuclear SSR data (Honig et al. 2015), *A. canina* was more closely related to *A. capillaris* than to *A. stolonifera*, contrary to the ITS tree in Rotter et al. (2010), whereas based on plastid SSR data, *A. canina* was more closely related to *A. stolonifera* than to *A. capillaris*, similar to the plastid tree in Rotter et al. (2010). Honig et al. (2015) suggested the close plastid relationship between *A. canina* and *A. stolonifera* may be due to introgression of the *A. canina* plastome into *A. stolonifera*, rather than *A. canina* being one of the parent species (the maternal one) of *A. stolonifera*. Their hypothesis of plastid introgression does not, however, explain the close relationship of *A. canina* and *A. stolonifera* in the ITS tree in Rotter et al. (2010) contradicting their nuclear SSR data. On the other hand, phenetic cluster analyses of inter-population pairwise genetic distances of SSR data may not necessarily reflect nuclear gene trees.

Amundsen and Warnke (2012) also produced a plastid phylogeny focused on the commercial species of *Agrostis*, and they included six other taxa in their analyses. They identified three main plastid lineages in a *trnL–trnF* tree and four lineages in an *atpI–atpH* tree, with several species (*A. gigantea*, *A. stolonifera*, *A. trinii* Turcz.) present in more than one lineage. Some of their samples may be misidentified because most were grown from seed obtained from the National Plant Germplasm System; this could explain the infraspecific variation. It is unclear if the plants were grown to an identifiable stage, and if voucher specimens were prepared (Amundsen and Warnke 2012). Indeed, Honig et al. (2015) found identification errors in material of *Agrostis* obtained from this seed bank. On the other hand, the infraspecific variation may be real. Amundsen and Warnke (2012) and Honig et al. (2015) both sampled the same two accessions of *A. stolonifera* from the National Plant Germplasm System (PI 302902 and PI 318934) and plastid DNA in these samples did not group with other accessions of *A. stolonifera*. We are confident in the accuracy of these plastid data because they were generated independently from the same samples, although the possibility remains the samples are misidentified. In Amundsen and Warnke (2012), the two accessions of *A. stolonifera* were part of a clade with *A. capillaris*, accessions of *A. gigantea*, *Polypogon* and a few other taxa of *Agrostis*, and in Honig et al. (2015) they were part of a clade with *A. capillaris* and *A. gigantea*. By contrast, based on nuclear data in Honig et al. (2015) these samples grouped with other accessions of *A. stolonifera*. These incongruent plastid and nuclear data provide evidence for putative chloroplast capture in the two accessions of *A. stolonifera*.

Although the current taxon sampling in *Agrostis* is relatively limited in the context of overall species diversity in the genus, our analyses add new knowledge to our understanding of *Agrostis* phylogeny and confirm a close relationships between *Agrostis*, *Chaetopogon*, *Polypogon* and *Lachnagrostis* (Quintanar et al. 2007, Saarela et al. 2010). The ITS+ETS tree identifies two strongly supported clades: one includes *Polypogon elongatus* and all sampled species of *Agrostis* except *A. exarata* and *A. rosei*, the other includes the other sampled species of *Polypogon*, one species of *Lachnagrostis* and *A. exarata*. Although sampling in the ITS+ETS tree is poorer than in the ITS tree, combining ITS and ETS data substantially increases resolution and support in the *Agrostis* clade compared to the ITS tree. The first main clade in ITS+ETS tree includes three main subclades comprising the following species (ploidy for species is indicated if known and not already stated): (1) *A. gigantea* and *A. capillaris*; (2) *A. mertensii* (2*n*=42) and *A. stolonifera*; and (3) *A. breviculmis* Hitchc., *A. gelida* Trin., *A. hallii* Vasey (2*n*=42), *A. imberbis* Phil., *A. meyenii* Trin., *A. tolucensis* Willd. ex Steud. (2*n*=28) and *P. elongatus*. The ITS tree includes subclades corresponding to those in the ITS+ETS tree, with increased taxon sampling. One subclade includes multiple accessions of *A. gigantea*, *A. capillaris* and *A. clavata* Trin., plus one accession each of *A. castellana*, *A. hyemalis* (Walter) Britton, Sterns & Poggenb., *A. stolonifera* and *Chaetopogon fasciculatus*. A second subclade includes multiple accessions of *A. canina*, *A. mertensii* and *A. stolonifera* plus one accession each of *A. personata*, *A. imbecillata*, *A. pallescens*, *A. petriei*, *A. muscosa*, *A. muelleriana* and *A. magellanica*, and three taxa of *Lachnagrostis*. A third subclade includes multiple accessions of *A. breviculmis*, *A. scabra* (2*n*=42), *A. tolucensis* and *A. vinealis*, and single accessions of *A. hallii*, *A. idahoensis*, *A. magellanica*, *A. meyenii* Trin. and *A. pallens*.

*Chaetopogon* Janchen is a monotypic genus characterized by having an annual habit, panicles moderately dense, spikelets lacking rachilla extension and falling entire, and lower glumes becoming a long slender awn (Clayton and Renvoize 1986). Some workers included *Chaetopogon* in *Polypogon* (*P. fasciculatus* (Link) Pers.) with which it shares some morphological features (awned glume, spikelet falling entire). Kellogg (2015) treated *Chaetopogon* as a synonym of *Agrostis* based on the ITS phylogeny in Saarela et al. (2010), which is consistent with the current ITS phylogeny. In the *trnL-trnF* tree (Suppl. material 11), however, *C. fasciculatus* is excluded from a strongly supported *Agrostis* p.p. clade, and allied with *Polypogon*, *A. capillaris* p.p., *A. gigantea* p.p. and a few other taxa, congruent with the plastid tree in Quintanar et al. (2007). Support for placement of *C. fasciculatus* in the *trnL-trnF* tree is weak, but the overall topology of that tree is consistent with the better-resolved combined plastid tree here. In other words, there is incongruence between nrDNA and plastid data of *C. fasciculatus*, and the genus likely has a hybrid origin.

Our plastid tree identifies a strongly supported *Agrostis* + *Polypogon* clade, with two major subclades. One subclade includes *A. breviculmis*, *A. capillaris*, *A. gigantea*, *A. hallii*, *A. imberbis*, *A. stolonifera*, *A. scabra* and *A. tolucensis*, encompassing most taxa in the second and third ITS subclades described above. The other subclade includes *A. capillaris*, *A. gigantea* and all species of *Polypogon*, corresponding in part to the first ITS subclade described above. Placement of *A. gigantea* and *A. capillaris* in a subclade with all species of *Polypogon* in the plastid tree represents strong incongruence with the nrDNA trees, in which all *Agrostis* species except *A. exarata* are closely related to one another. The *matK* tree (Suppl. material 7) identifies the same two main plastid lineages with greater sampling of individuals and taxa compared to the combined plastid tree. Along with multiple samples of *Polypogon*, *A. capillaris* and *A. gigantea*, a subclade in the *matK* tree includes five additional species of *Agrostis* (*A. curtisii* Kerguélen, *A. elliotii* Hack., *A. producta* Pilg., *A. tenerrima* Trin., *A. transcaspica* Litv.) and two species of *Lachnagrostis*. *Lachnagrostis* is a genus of ca. 20 species of annuals and perennials with inflorescences often shedding and dispersing as a whole, spikelets one- to (sometimes) two-flowered with a rachilla extension, glumes longer than the florets, callus with hairs up to ca. 2/3 the length of the lemma, and lemma with minute teeth at the apex, unawned or with an abaxial awn, the awn straight or geniculate (Kellogg 2015). There is no overlap in the species of *Lachnagrostis* in the ITS and *matK* trees, so we are unable to make conclusions about the affinities of *Lachnagrostis* and possible incongruence between plastid and nrDNA regions with the current sampling. However, with a much broader sampling of *Lachnagrostis*, Brown (2013) found the genus not to be monophyletic based on nuclear and plastid data, and also found some incongruence between these genomes. We did not sample the two additional plastid lineages of *Agrostis* identified in the trees in Amundsen and Warnke (2012). One lineage was represented by *A. lyalii* Hook. f. and *A. limprichtii* Pilg., and the other by *A. mongolica* Roshev., *A. trinii* Turcz. and *A. castellana*. The sequences reported in Amundsen and Warnke (2012) are not available in GenBank. Future work should aim to confirm and better characterize these lineages.

Different placements of *Agrostis exarata* in the nrDNA and plastid trees suggest this species has an allopolyploid origin. The same discordance is present in the ITS and *matK* trees in Reichman et al. (2006), but they did not comment on it, and in the sample reported in Quintanar et al. (2007) and Saarela et al. (2010). *Agrostis exarata* is a morphologically variable polyploid species (2*n*=28, 42, 56) distributed throughout western North America (Björkman 1960, Harvey 2007a). *Agrostis exarata* is unusual in North America, being one of few species of *Agrostis* with long-awned (1 mm or more) glumes in some individuals (in others glumes are acute, not awned); long-awned glumes is one character used to circumscribe *Polypogon*. Awned and unawned individuals of *A. exarata* have been variously treated as different taxa (Hitchcock 1905, 1915, 1934, Beetle 1945). Long-awned glumes in some individuals of *A. exarata* may reflect hybrid parentage involving a species of *Polypogon*. The samples of *A. exarata* studied here, however, lack awns. Molecular sampling of morphologically variable individuals now included in *A. exarata*, and other *Agrostis* taxa with awned glumes, are needed to clarify whether or not awned and unawned individuals have separate origins and to determine their relationships to each other and to *Polypogon*.

Several other instances of incongruence between nrDNA and plastid trees are present in individuals and species of *Agrostis*: (1) individuals of *A. capillaris* (*Saarela 748*) and *A. mertensii* (*Peterson 20884*) fall in different subclades in plastid and nrDNA trees; (2) accessions of *A. gigantea* are placed in each major plastid subclade; and (3) accessions of *A. mertensii* are part of different subclades in the ITS+ETS tree. The observed variation among the *A. capillaris*, *A. gigantea* and *A. mertensii* samples we sequenced does not seem to be attributable to misidentification, as we carefully reviewed the voucher specimens to ensure the material was correctly identified. Multiple accessions of the hexaploid *A. gigantea*, in addition to ones we sequenced, are also present in both major clades in the *matK* tree (Suppl. material 7), confirming the presence of deep infraspecific plastid variation in the species. Deep genetic variation in *A. gigantea* was also found in an AFLP-based study of *Agrostis* (Vergara and Bughrara 2003). A close relationship between some individuals of *A. gigantea* (A_1_A_1_A_2_A_2_A_3_A_3_) and *A. capillaris* (A_1_A_1_A_2_A_2_), and some individuals of *A. gigantea* and *A. stolonifera* (A_2_A_2_A_3_A_3_), representing divergent lines in the genus, may be a reflection of the shared portions of their genomes, or may be due to hybridization, introgression and/or multiple origins of the taxa. Whatever the origin, breeders working with *Agrostis* should be aware that deep genetic variation exists in *A. gigantea* and *A. stolonifera*, and possibly other species.

*Agrostis stolonifera* is now understood to have arisen from hybridization between two diploids, possibly *A. canina* (type species of Agrostis sect. Agrostis) representing the A_2_ genome and *A. transcaspica* (=A. stolonifera subsp. transcaspica (Litv.) Tzvelev), representing the A_3_ genome (Rotter et al. 2010, Reichman et al. 2011). *Agrostis transcaspica* was determined as diploid by Vergara and Bughrara (2003). *Agrostis canina* has a Trichodium net on the lemma, whereas *A. transcaspica* has a tendency towards a Trichodium net on the lemma (groups 1 and 3 in Björkman 1960). *Agrostis stolonifera*, on the other hand, has a lemmatal network only fragmentarily developed (group 2 in Björkman 1960). This morphology, intermediate between the lemma morphology of *A. canina* and *A. transcaspica*, is consistent with a hybrid origin of *A. stolonifera* involving the two putative parental taxa. The hypothesized parentage of *A. stolonifera* is also consistent with the current phylogenetic evidence. In the ITS phylogeny, all accessions of *A. canina* and all but one accession of *A. stolonifera* are part of the same clade, consistent with the tree in Rotter et al. (2010) and with the hypothesis of *A. canina* being one parent of *A. stolonifera*. ITS has not been sequenced for *A. transcaspica*, and neither of the putative parental taxa have had their ETS regions sequenced, but in the *matK* tree (Suppl. material 7), a sequence for *A. transcaspica* from the same accession (PI 283174) sampled by Vergara and Bughrara (2003) is part of the subclade including *A. capillaris* and *A. castellana*, as in Reichman et al. (2011). This is consistent with the hypothesis of *A. transcaspica* being the paternal parent of *A. stolonifera* (Reichman et al. 2011); if *A. transcaspica* were the maternal parent, it and *A. stolonifera* should be in the same plastid clade. Despite the affinities of the plastomes of *A. capillaris* and *A. transcaspica*, the latter species has not been implicated in the evolution of *A. capillaris* or *A. castellana*. It might also, however, represent the origin of the A_3_ genome in *A. gigantea* (Reichman et al. 2011), a hypothesis consistent with the plastid tree (although *A. gigantea* individuals are present in both major clades and the origin(s) of this variation is unknown) and also with lemma epidermal morphology because both *A. gigantea* and *A. transcaspica* have a lemmatal network wanting (Björkman 1960).

Most species of *Agrostis* newly sampled here have short paleas relative to the length of the lemmas. On the basis of this character, these taxa would be classified in Agrostis sect. Agrostis as traditionally defined. Species sampled with long paleas relative to the lemmas include *A. capillaris* (palea 0.5–0.7× lemma length), *A. castellana* (0.5×), *A. gelida* (0.4–0.5×), *A. gigantea* (0.5–0.7×) and *A. stolonifera* (0.6–0.8×). These would be classified in Agrostis sect. Vilfa. In the trees, species of both sections are intermixed, indicating neither section is monophyletic. Application of the sectional name *Vilfa* in the context of phylogenetic information is problematic from an evolutionary perspective because its type species, *A. stolonifera*, is an allopolyploid, and one of its putative parental taxa, *A. canina*, is the type species of *Agrostis* and of Agrostis sect. Agrostis. In other words, putative ancestor (*A. canina*) and descendant species (*A. stolonifera*) are type species of different subdivisions of the genus. Because *A. canina* and *A. stolonifera* are part of the same clade in plastid and nrDNA trees, the sectional name *Vilfa* is a synonym of Agrostis sect. Agrostis. Subdivisional classification of *Agrostis* should be revisited in the context of a comprehensive molecular phylogeny of the genus and its close relatives.

*Polypogon* and *Agrostis* are closely related and neither is monophyletic in plastid and nrDNA trees. *Polypogon* Desfontaines (1798-1799) (type *P. monspeliensis*) is a genus of 26 diploid to polyploid (2*n*=14, 28, 35, 42, 56) species distributed in temperate areas of both hemispheres. *Polypogon* differs from *Agrostis* by having spikelets disarticulating below the glumes (vs. above the glumes), a broader and more truncate lemma, awned glumes (vs. unawned), photosynthetic tissue of the lemma covering most of the lemma (vs. continuous in the lower part of the lemma and extending along the nerves distally), paleas with a bundle of small elongated cells in each tip if two-tipped (vs. palea tips single-pointed if two-tipped, or rarely ca. aristate) and caryopses broadest above the middle (vs. broadest at or below the middle) (Björkman 1960, Kellogg 2015). *Polypogon* was divided into two sections by Ascherson and Graebner (1899): Polypogon sect. Eupolypogon, nom. inval. (=Polypogon sect. Polypogon) and Polypogon sect. Polypogonagrostis. Björkman (1960) noted similarities between Polypogon sect. Polypogonagrostis and *Agrostis*, including relatively small paleas, elongated caryopses, sparse photosynthetic tissue on the lemma and glossy lemma surfaces. He also noted similarities between Polypogon sect. Polypogonagrostis and Polypogon sect. Polypogon, including scabrous glumes, awned glumes, spikelets disarticulating below the glumes, difficulty separating the floret from the rachilla and subterminal insertion of the lemma awn. Some authors have recognized species of Polypogon sect. Polypogonagrostis in the genus *Chaetotropis* Kunth (Kunth 1829–1835, Björkman 1960, Nicora 1970, 1978, Nicora and Rúgolo de Agrasar 1987, Rúgolo de Agrasar 2012), whereas others have considered *Chaetotropis* a synonym of *Polypogon* (Hitchcock 1951, Clayton and Renvoize 1986, Tovar 1993, Barkworth 2007b, Finot et al. 2011b, 2013). In a classification of *Polypogon* in South America, Müller (1985) treated six species in Polypogon sect. Polygon (*P. australis*, *P. interruptus*, *P. linearis* Trin., *P. maritimus* Willd., type *P. monspeliensis*, *P. viridis*) and four in Polypogon sect. Polypogonagrostis (*P. chilensis* (Kunth) Pilg., type *P. elongatus*, *P. rioplatensis* Herter and *P. imberbis* (Phil.) Johow). *Polypogon* was recently revised in Colombia (Giraldo-Cañas 2004) and Chile (Finot et al. 2004). Finot et al. (2011b) studied lemma epidermis morphology of species of *Polypogon*, *Agrostis* and the hybrid ×*Agropogon* P. Fourn. in Chile. In their analyses, nine species of *Polypogon* clustered into groups corresponding to *Polypogon* sects. *Polypogon* and *Polypogonagrostis*. Molecular phylogenetics of *Polypogon* have not previously been studied in detail.

We newly sampled four species of Polypogon sect. Polypogon (*P. australis*, *P. interruptus*, *P. monspeliensis* and *P. viridis*) and one of Polypogon sect. Polypogonagrostis (*P. elongatus)*. The ITS tree also includes previously published accessions of *P. fugax* Nees ex Steud. and *P. maritimus*. Although the current sampling is the most comprehensive to date for *Polypogon*, over half of its species-level diversity remains to be sampled. Nevertheless, our analyses provide new insights into the evolutionary history of the genus.

Affinities of some species of *Polypogon* differ in plastid and nrDNA trees. In the plastid tree, all species of *Polypogon* (i.e. both sections) plus *Agrostis capillaris* and *A. gigantea* form a strongly supported clade. The *matK* tree (Suppl. material 7) includes additional species of *Agrostis* in this clade. In the ITS and ITS+ETS trees, however, *P. elongatus* is nested in the *Agrostis* clade among species with a Trichodium net on their lemma epidermises. All species of Polypogon sect. Polypogonagrostis have this lemma epidermal pattern (Björkman 1960). This discordance between nrDNA and plastid data is strong evidence for a hybrid origin of *P. elongatus* involving species of *Agrostis* and Polypogon sect. Polypogon. *Polypogon elongatus* is polyploid (2*n*=28, 56) (Bowden and Senn 1962, Pohl and Davidse 1971), consistent with this hypothesis. Inclusion of the other three species of Polypogon sect. Polypogonagrostis in future molecular work is needed to determine if they also have hybrid origins.

In the ITS+ETS tree, the four sampled species of Polypogon sect. Polypogon form a strongly supported clade with *Agrostis exarata* and a species of *Lachnagrostis*, but the affinities of this clade with the main *Agrostis* clade and other taxa of Agrostidinae are unresolved. This clade is also present in the better-sampled ITS tree, including five species of Polypogon sect. Polypogon, *A. exarata* and cloned sequences from an *A. stolonifera* × *P. monspeliensis* hybrid (Zapiola and Mallory-Smith 2012). Cloned sequences from the same hybrid individual are also present in the *Agrostis* clade. Zapiola and Mallory-Smith (2012) identified *A. stolonifera* as the maternal parent in the cross. *Agrostis stolonifera* is an outcrossing species that hybridizes with at least 12 other species of *Agrostis* and *Polypogon* (Belanger et al. 2003a, Belanger et al. 2003b, Reichman et al. 2006). Hybrids between *A. stolonifera* and *P. monspeliensis* are sometimes recognized as ×*Agropogon lutosus* (Poir.) P. Fourn. (Soreng and Greene 2003) or ×*Agropogon littoralis* (Sm.) C.E. Hubb, an illegitimate name (Zapiola and Mallory-Smith 2012).

The Polypogon sect. Polypogon + *Agrostis exarata* + *Lachnagrostis* clade in the ITS+ETS tree is divided into two strongly supported subclades. One subclade includes *P. viridis*, *P. monspeliensis* and *A. exarata*. The other subclade includes *P. australis*, *P. interruptus* and *L. adamsonii*. This clade is also resolved in the ITS tree with the same general topology; clades of *P. australis* and *P. interruptus* and of four species of *Lachnagrostis* are sister groups. The placement of *Lachnagrostis* in the current trees is consistent with the findings of Brown (2013), who included much broader sampling of *Lachnagrostis* and found the genus to polyphyletic. In his ITS tree, a strongly supported clade includes the following four successively diverging lineages: (1) *L. punicea* (A.J. Br. & N.G. Walsh) S.W.L. Jacobs; (2) a clade comprising four species of Polypogon sect. Polypogon (*P. maritimus*, *P. fugax*, *P. viridis* and *P. monspeliensis*); (3) the Australian species *P. tenellus* R. Br.; and (4) a clade comprising most species of *Lachnagrostis*, including the type, *L. filiformis* (G. Forst.) Trin. Some New Zealand species of *Lachnagrostis*, however, formed a clade with most species of *Agrostis* in Brown (2013). The plastid trees in Brown (2013) have a similar topology, except the South African and New Zealand species are part of the main *Lachnagrostis + Polypogon* clade, and there is evidence of reticulation in the origins of some species.

We sampled three accessions of *Polypogon viridis* (=*P. semiverticillatus* (Forssk.) Hyl.), a species whose generic placement has varied. Many authors have treated the taxon in *Agrostis* (*A. semiverticillata* (Forssk.) C. Chr.) given its lack of awns on the glumes (Hitchcock 1951, Bor 1960, Gould and Moran 1981). Beetle (1950) placed *A. semiverticillatus* in its own section, Agrostis sect. Vilfoidea (Rouy) Beetle. However, spikelets of *A. semiverticillatus* disarticulate below the glumes, as in *Polypogon*, and on this basis it has been placed in *Polypogon* (Hylander 1945, Tzvelev 1983, Edgar 1991, Zuloaga et al. 1994, Edgar and Connor 2000, Giraldo-Cañas 2004, Barkworth 2007b, Finot et al. 2013, Brown 2015). With the exception of awned glumes, the species has all spikelet characteristics defining *Polypogon* (Björkman 1960). Brown (2015) showed *P. viridis* taxon lacks a Trichodium net on the lemma epidermis, consistent with a placement in Polypogon sect. Polypogon, whose species lack a Trichodium net, in contrast to most species of *Agrostis* (see earlier). Finot et al. (2011b) also considered the taxon to be best placed in *Polypogon* based on the absence of a Trichodium net and other micromorphological characters. *Polypogon viridis* is part of the Polypogon sect. Polypogon clade in the nrDNA and plastid trees, confirming its generic placement for the first time with molecular data.

#### *Podagrostis*

*Podagrostis* (Griseb.) Scribn. & Merr. has been variously recognized as a distinct genus or included in *Agrostis*. Agrostis sect. Podagrostis was defined by Grisebach (1853) to include the western North American species *A. aequivalvis* Trin. (the type), characterized by having short glumes and a well-developed palea. Hitchcock (1905) later included the western North American species *A. thurberiana* Hitchc. in Agrostis sect. Podagrostis. The section was subsequently elevated to genus rank (Scribner and Merrill 1910). Björkman (1960) transferred the western North American species *A. humilis* Vasey to *Podagrostis* (*P. humilis* (Vasey) Björkman) and differentiated *Podagrostis* from *Agrostis*, in part, by the lack of a Trichodium net on the lemma epidermis. A fourth species of *Agrostis*, *A. sesquiflora* E. Desv., from Patagonia, was transferred to *Podagrostis* (*P. sesquiflora* (E. Desv.) Parodi ex Nicora) by Nicora (1978), a classification first suggested by Björkman (1960). These four species are now recognized in *Podagrostis* (Soreng 2003, Harvey 2007b), although some authors continue to treat them in *Agrostis* (Clayton et al. 2006 onwards). *Podagrostis humilis*, *P. thurberiana* and *P. aequivalvis* are diploids (2*n*=14) (Harvey 2007b). *Podagrostis thurberiana* was included in a phylogenetic study based on morphology and three plastid regions, and the taxon was weakly supported as the sister group of a strongly supported *Agrostis + Polypogon* clade (Soreng et al. 2007). A limitation of that analysis, however, is only a single species each of the three genera was included. No molecular study has included more than one species of *Podagrostis*, thus the monophyly of the putative lineage (regardless of its generic classification) has not been tested.

We sampled *Podagrostis aequivalvis*, a species not previously included in a molecular study. In the nrDNA trees its affinity to other taxa of Agrostidinae is unresolved, although it is not part of the *Agrostis* + *Polypogon* clade. By contrast, *Podagrostis aequivalvis* and the endemic California species *Calamagrostis bolanderi* are strongly supported sister taxa in the plastid tree; in the ITS+ETS tree, affinities of *C. bolanderi* and other taxa of Agrostidinae are unresolved. No association between *P. aequivalvis* and *C. bolanderi* has been suggested previously. However, Nygren (1954) considered *C. bolanderi* to “have an isolated position in the genus” in North America, consistent with the plastid data indicating *C. bolanderi* is not closely related to most other species of *Calamagrostis*, but provided no rationale for this statement. In the plastid tree, the *P. aequivalvis* + *C. bolanderi* clade is sister to a weakly supported clade including Chinese species of *Deyeuxia*, the Mexican species *Agrostis rosei*, and the *Agrostis* + *Polypogon* clade. This placement of *A. rosei*, a diploid with a palea greater than half the lemma length (Herrera Arrieta et al. 2010), apart from the main *Agrostis* lineages reported here is unexpected and requires confirmation. Placement of *P. aequivalvis* in the plastid tree is congruent with placement of *P. thurberiana* sister to *Agrostis* + *Polypogon* in Soreng et al. (2007), despite their poorer taxon sampling. Although the precise affinities of *P. aequivalvis* remain unclear, at least in nrDNA trees, *P. aequivalvis* is not part of the *Agrostis* lineage. *Podagrostis* should not be treated as a synyonym of *Agrostis*, unless, perhaps, if *Agrostis* were to be circumscribed more widely.

#### *Calamagrostis*/*Deyeuxia* and *Ammophila*

The taxonomic history of *Calamagrostis* and *Deyeuxia* is complex (Vickery 1940, Rúgolo de Agrasar 1978, Escalona 1988b). *Calamagrostis* was described by Adanson (1763). No species were cited with the description, but the genus is thought to have been based on *Arundo epigeios* L. and *A. calamagrostis* L. (Vickery 1940). *Arundo calamagrostis* is accepted as the type species of *Calamagrostis* (Hitchcock 1937). The accepted name for *A. calamagrostis* (=*C. lanceolata* Roth) is *C. canescens*, a Eurasian species. *Deyeuxia* Clarion in Beauvois (1812) was named after the chemist M. Deyeux, for species similar to *Calamagrostis* with extended rachillas. A lectotype, *D. montana* P. Beauv. (=*C. arundinacea*), based on *Arundo montana* Gaud., an illegitimate synonym of *Arundo varia* Schrad., was designated for the genus by Niles (1925).

Since 1812, authors have variously recognized *Calamagrostis* and *Deyeuxia* as distinct genera (Bentham 1882 [1881], Bentham and Hooker 1883, Jansen 1952, Lu 1987, Phillips and Chen 2003, Lu et al. 2006, Clayton et al. 2006 onwards) or a single genus (Dumortier 1823, Nees von Esenbeck 1829, Koch 1837, Steudel 1840-1841, 1854, Weddell 1875, Ascherson and Graebner 1899, Hitchcock 1927b, Stebbins 1930, Tzvelev 1976, Koyama 1987, Soreng and Greene 2003, Ma et al. 2006, Marr et al. 2007, Peterson and Saarela 2012), based on the presence or absence of a rachilla extension and differences in glume length, callus vestiture and rachilla vestiture. In global lists of grasses, Clayton et al. (2006 onwards) accepted 225 species of *Deyeuxia* and 46 of *Calamagrostis* s.str., whereas Simon (2014) accepted 217 species of *Calamagrostis* s.str. and 76 of *Deyeuxia*. Kellogg (2015), acknowledging the problematic circumscription of these genera, recognized 98 species of “*Calamagrostis*”, and 207 of *Deyeuxia*.

In the Eastern Hemisphere, species morphologically similar to *Calamagrostis* and *Deyeuxia* have also been placed in *Agrostis* (Vickery 1940, Zotov 1965, Edgar 1995). A series of recent papers has clarified numerous lower-level taxonomic issues among multiple Eurasian taxa of *Agrostis*, *Calamagrostis* and *Deyeuxia* (many from the far East) and described new species, most not yet included in molecular studies (Paszko and Nobis 2010, Paszko 2011, 2012a, b, 2013, 2014a, b, c, d, e, 2015, 2016, Paszko and Ma 2011, Paszko and Chen 2013, Paszko and Pendry 2013a, b, Paszko and Soreng 2013, Paszko et al. 2013, 2015, Nobis et al. 2014). In Mexico and Central America, species are treated in *Calamagrostis* (Beetle 1987, Pohl and Davidse 1994, Peterson et al. 2004). Many of these were initially described as species of *Deyeuxia* by Kunth in Humboldt et al. (1815 [1816]). In South America, the species have mostly been recognized in either *Calamagrostis* (Swallen 1948, Parodi 1949b, Tovar 1960, 1984, 1985, 1993, Escalona 1988ba, 1988b, Laegaard 1998, Giraldo-Cañas 2012) or *Deyeuxia* (Rúgolo de Agrasar 1975, 1978, 1985, 1986a, b, Escalona 1991, Rúgolo de Agrasar and Villavicencio L. 1995, Villavicencio 1995, Finot et al. 2011a). Most South American species of *Calamagrostis*/*Deyeuxia* are part of Koeleriinae clade B.

We have considerably expanded sampling of north temperate species and individuals of *Calamagrostis*/*Deyeuxia* compared to an earlier study, in which their phylogenetic relationships were poorly resolved and supported (Saarela et al. 2010). Relationships among taxa of *Calamagrostis*/*Deyeuxia* within Agrostidinae are mostly unresolved with respect to each other and to the various clades of *Agrostis* and *Polypogon* in the plastid and nrDNA trees. Despite the general lack of backbone structure, a few moderately to strongly supported multi-species clades are present in the trees. The type species of *Calamagrostis* (*C. canescens)* and *Deyeuxia* (*C. arundinacea*) are among these. Accordingly, the phylogenetic data do not support recognition of *Calamagrostis* and *Deyeuxia* as separate genera under any circumstance: *Deyeuxia* is a synonym of *Calamagrostis*. Additionally, a sample identified as *Neoschischkinia truncatula* (=*Agrostis truncatula*) is allied with *Calamagrostis*/*Deyeuxia* in the ITS tree. Authors have recognized *Neoschischkinia* Tzvelev as a genus differing from *Agrostis* by having an annual or short-lived perennial habit, and lax panicles often with more or less divaricate branches usually clavate at the apex (Valdés and Scholz 2006), and five species have been recognized (Tzvelev 1968, Valdés and Scholz 2006). Sampling of the plastid and nrDNA data from the five species is needed to confirm their affinities.

Multispecies lineages of *Calamagrostis*/*Deyeuxia* supported in the trees include two to numerous species, and several species are not monophyletic. Two of the three samples of *C. anthoxanthoides* and *C. holciformis*, both western Eurasian taxa, form a clade in the ITS+ETS tree, but in the plastid tree their affinities are unresolved. The Californian endemics *C. foliosa* and *C. bolanderi* form a clade in the ITS+ETS tree, but not in the plastid tree (affinities of *C. bolanderi* with *Podagrostis* in the plastid tree are discussed above). The morphologically similar European species *C. canescens* (type of *Calamagrostis*) and *C. villosa* (Paszko 2011) form a strongly supported clade in the ITS+ETS tree. These species are also closely related in the plastid tree, and they are part of a clade including two of the three samples of the endemic German species *C. rivalis* (=*C. pseudopurpurea* Gerstl. ex. O.R. Heine) (Raus and Scholz 2002). The other sample of *C. rivalis* is part of a separate clade in the plastid tree, allied with *C. pseudophragmites*. In the ITS+ETS tree, this sample has the same affinity, as does the other *C. rivalis* sample included in the tree. Different placements of *C. rivalis* in plastid and nrDNA trees are potentially consistent with multiple hybrid origins of the taxon involving parental species from each clade (Schiebold et al. 2009). In the plastid tree, this three-taxon clade (*C. canescens*, *C. rivalis*, *C. villosa*) is part of a broader clade including *C. stricta* p.p. (samples from Eurasia and the Americas), *C. lapponica* p.p. and a few other species. Calamagrostis × gracilescens is part of a clade including all accessions of *C. stricta*, C. stricta subsp. stricta and C. stricta subsp. inexpansa in the ITS+ETS tree, consistent with *C. stricta* being one of the putative parents of this hybrid (Paszko 2011). However, the Arctic taxon C. stricta subsp. groenlandica is not part of this clade; it groups with the northern species *C. purpurascens*, *C. deschampsioides* and *C. lapponica* p.p. in the ITS+ETS tree. Some specimens identified here as C. stricta subsp. groenlandica have also been determined as *C. holmii* Lange, a species recognized by some authors (Tzvelev 1976, Elven et al. 2011) that we treat as a synonym of C. stricta subsp. groenlandica (J.M. Saarela, unpublished data). Resolution in the plastid tree is poorer and all samples of *C. stricta* s.l. are part of a broader and poorly resolved clade including several other species.

Although most sampled species of *Calamagrostis*/*Deyeuxia* from Mexico to South American are part of Koeleriinae clade B or the *Deschampsia* clade and unrelated to *Calamagrostis*/*Deyeuxia* s.str., two species from northern South America and one from Central America resolve among other species of *Calamagrostis*/*Deyeuxia* s.str. The Ecuadorian endemics *C. carchiensis* and *C. llanganatensis* (Laegaard 1998), of which we sampled paratypes, are sister taxa in plastid and nrDNA trees, and form a polytomy with numerous species and multi-species lineages of *Calamagrostis*/*Deyeuxia*. As such, we are unable to make any inferences about their putative geographical origins. Nevertheless, they represent the known southern limit of *Calamagrostis*/*Deyeuxia* s.str. in the New World. Laegaard (1998) noted the small purplish spikelets lacking a rachilla extension of *C. llanganatensis* to be superficially similar to spikelets of *Agrostis*. The molecular data confirm the taxon is a species of *Calamagrostis*/*Deyeuxia* s.str. Other species of *Calamagrostis*/*Deyeuxia* from Ecuador described by Laegaard (1998) have not yet been sampled molecularly. One of these, *C. teretifolia* Laegaard, is a stipitate species and may be allied to the species of Deyeuxia sect. Stylagrostis that are part of the *Deschampsia* clade. The Central American species *C. guatemalensis* (Hitchcock 1927b, Pohl and Davidse 1994) is part of a moderately supported clade in the plastid tree including several North American species of *Calamagrostis* (*C. cainii*, *C. foliosa*, *C. howellii*, *C. koelerioides*, *C. pickeringii*, *C. purpurascens*, *C. rubescens*, *C. scopulorum*, *C. sesquiflora*) and *Ammophila breviligulata*. In the ITS+ETS tree, *C. guatemalensis*, *C. cainii* and C.koelerioides form an unsupported clade. Given the apparent close relationship between *C. guatemalensis* and species from the United States and Canada, it is surprising that no species of *Calamagrostis*/*Deyeuxia* s.str. are known from Mexico. Affinities of the few unsampled Central American species of *Calamagrostis* (*C. nuda* Pilg., *C. pinetorum* Swallen, *C. pittieri* Hack.) are unknown.

Several Eurasian species (*C. brachytricha*, *C. distantiflora*, *C. arundinacea* p.p., *D. diffusa* [=*C. diffusa* (Keng) P.C. Kuo & S.L. Lu ex J.L. Yang], *D. pulchella* [=*C. lahulensis* G. Sing] and *D. scabrescens* [=*C. scabrescens* Griseb.]) plus the western North American species *C. nutkaensis* form a clade in the ITS+ETS tree. This supports the supposition of Nygren (1954) that *C. nutkaensis* may be related to eastern Asian species. In the plastid tree, however, *C. nutkaensis* is part of a broader clade comprising a different set of species. Accordingly, *Calamagrostis nutkaensis* may have an allopolyploid origin. A clade of Chinese species, including *D. diffusa* (=*C. flaccida* (Keng) Keng f.), *D. tripilifera* (=*C. tripilifera* Hook. f.), *D. mazzettii* (=*C. stenophylla* Hand.-Mazz.) and *D. nivicola* (=*C. nivicola* (Hook. f.) Hand.-Mazz.), is recovered in the ITS+ETS and plastid trees. In the plastid tree, this clade also includes *Agrostis rosei* and is weakly supported as sister to the large *Agrostis* + *Polypogon* clade. In the ITS+ETS tree, deep affinities of the clade are unresolved.

One strongly supported clade in the ITS+ETS tree corresponds, in part, to Calamagrostis sect. Deyeuxia as recognized by Paszko (2011). The clade includes several species native to Eurasia: *C. arundinacea* p.p. (type of *Deyeuxia*), *C. varia*, *C. emodensis*, *C. epigeios* p.p., *C. pseudophragmites*, *C. rivalis*, *D. nyingchiensis* (=C.nyingchiensis (P.C. Kuo & S.L. Lu) Paszko) and *D. sichuanensis* (=*C. sichuanensis* J.L. Yang), plus *Ammophila arenaria* and ×*Calammophila baltica*, an intergeneric hybrid of *A. arenaria* and *C. epigeios*. A similar clade is recovered in the plastid tree, excluding *A. arenaria* and the two Chinese species, whose affinities in the plastid tree are unresolved. The clade does not, however, correspond to Calamagrostis sect. Deyeuxia as circumscribed by Tzvelev (1976), who included in this section *C. purpurascens*, *C. sesquiflora* (Trin.) Tzvelev, *C. nutkaensis*, *C. deschampsioides*, *C. chalybaea*, *C. holciformis*, *C. anthoxanthoides* and *C. stricta*. Moreover, the clade includes *C. epigeios* p.p. and *C. pseudophragmites*, species Tzvelev (1976) treated in C. sect. Pseudophragmites Tzvelev. Chinese samples identified as *C. arundinacea*, however, are part of a clade with other Asian species. One of these is *C. distantiflora*, a taxon sometimes treated as C. arundinacea subsp. distantiflora (Luchnik) Tzvelev. Clarification of the taxonomy of the morphologically and molecularly variable *C. arundinacea* is needed.

Our phylogenetic trees do not support monophyly of two species complexes identified in a recent study of Chinese taxa. Paszko and Ma (2011) considered *C. epigeios*, *C. kengii* T.F. Wang (not sampled) and *C. macrolepis* to be part of the *C. epigeios* complex, and *C. pseudophragmites*, *C. emodensis* and *C. hedinii* Pilg. to be part of the widespread Eurasian *C. pseudophragmites* complex (see also Paszko 2013). Although all these taxa are part of the same broader clade here, neither complex is resolved as a monophyletic group. Some individuals of *C. epigeios* and *C. pseudophragmites* are more closely related to each other than to other members of their respective complexes, and the sample of *C. epigeios* from China is not part of the same subclade as the samples of *C. epigeios* from Europe. The morphological similarities that define the complexes may be symplesiomorphies. Similarly, Paszko (2016) noted *D. nyingchiensis* to be morphologically similar to *D. scabrescens*, but the two taxa are not closely related in the analyses. The sample of *D. nyingchiensis* (*Soreng 5578*, cited in Paszko 2016) and the two samples of *D. scabrescens* are part of separate clades of Eurasian taxa.

The relationship between *Ammophila* and *Calamagrostis* has been questionable. *Ammophila* is a small genus of two rhizomatous perennial species (*A. arenaria* and *A. breviligulata*) characterized by having rigid inrolled leaves, spiciform panicles, one-flowered spikelets with rachilla extensions, strongly keeled lemmas and calluses bearded (Clayton and Renvoize 1986). *Ammophila arenaria* is native to Eurasia and *A. breviligulata* to North America. The two species differ primarily by ligule length and shape (Barkworth 2007a). A third species, *A. champlainensis* F. Seym., from Lake Champlain in the northeastern United States (Seymour 1966), has been recognized at species or subspecies ranks (A. breviligulata subsp. champlainensis (F. Seym.) P.J. Walker, C.A. Paris & Barrington ex Barkworth), but a morphological study of variation in *Ammophila* in northeastern North America concluded that plants recognized as *A. champlainensis* are best treated as the single species *A. breviligulata* (Delisle-Oldham et al. 2008). The taxon was recently recognized as a subspecies (Haines et al. 2011). Multiple ploidy levels (2*n*=14, 28, 56) have been reported in *Ammophila* (Watson and Dallwitz 1992 onwards). *Ammophila* is ecologically distinct among taxa of Agrostidinae, being strongly adapted to coastal dunes. The species of *Ammophila* hybridize with species of *Calamagrostis*. ×*Calammophila baltica* is a hybrid between tetraploid (2*n*=28) and octoploid (2*n*=56) individuals of *C. epigeios* and *A. arenaria* (Westergaard 1943); multiple varieties of the hybrid have been recognized (Westergaard 1943, Klimko et al. 2007). ×*Calammophila don-hensonii* Reznicek & Judz., described from Michigan, is a hybrid between *A. breviligulata* and *C. canadensis* (Reznicek and Judziewicz 1996). Although most authors have recognized *Ammophila* as a distinct genus, it has been treated as synonymous with *Calamagrostis* and classified in Calamagrostis subg. Ammophila (Host) A. Gray (e.g., Gray and Sullivant 1848).

Our phylogenetic analyses clarify the relationship between *Ammophila* and *Calamagrostis*. We sampled both species of *Ammophila* and one of the hybrids. *Ammophila* is not monophyletic in any of the trees here. *Ammophila arenaria* is part of a clade with Eurasian taxa of *Calamagrostis*/*Deyeuxia* in the ITS+ETS tree, consistent with the Old World distribution of all taxa in this clade, whereas affinities of *A. arenaria* are unresolved in the plastid tree. This may be indicative of a hybrid origin for the species. *Ammophila breviligulata* and *C. porteri* form a clade in the ITS+ETS tree, a topology consistent with their New World distributions; both species are native to northeastern North America. In the case of *A. breviligulata*, the plastid and nrDNA trees are congruent, although resolution in the plastid tree is poorer. In the plastid tree, the two species are part of a broader clade including a subset of North American species plus the Central American species *C. guatemalensis*. The two sampled individuals of ×*Calammophila baltica* are genetically distinct in the ITS+ETS tree; one is closely related to *A. arenaria* and the other is unresolved along the backbone of the clade including both samples. Given the observed nrDNA variation, the two samples may only share a single parent in common, although we are not aware of reports of hybrids involving *A. arenaria* and other taxa of *Calamagrostis* in Eurasia. Alternatively, multiple nrDNA gene copies may be present. In the plastid tree, both samples of ×*Calammophila baltica* are part of a clade with *C. epigeios*, *C. pseudophragmites*, *C. rivalis*, *C. varia* and C. × acutiflora. This topology is consistent with *C. epigeios* being the maternal parent of the hybrid individuals. Given the phylogenetic results, we propose to treat *Ammophila* as a synonym of *Calamagrostis*. A name in *Calamagrostis* is available only for *A. arenaria*, viz. *C. arenaria* (L.) Roth., therefore the needed combinations for *A. breviligulata*, A. breviligulata subsp. champlainensis, ×*Calammophila baltica* and ×*Calammophila don-hensonii* are made here (see Taxonomy).

The contracted panicles and large spikelets of the two unrelated species of *Ammophila* that we now recognize in *Calamagrostis* may be due to selection related to their habitat. Other examples of selection for contracted panicles and large spikelet in pooid grasses that grow in sand dunes include *Poa douglasii* Nees and *P. macrantha* Vasey (Poa sect. Madropoa Soreng) in North America, *P. cumingii* Trin. (sect. Dioicopoa E. Desv.) in South America, and *P. billardierei* St. Yves (sect. Austrofestuca (Tzvelev) Soreng & L.J. Gillespie) in Australia. The Eastern Asian steppe sand dune genus *Psammochloa* Hitchc. (Stipeae) also has a contracted panicle with large spikelets, and looks superficially like *Ammophila*, but it has very different lodicules (three in number that are flabellate and vascularized), a short cauducous awn from between two lobes, and nerves in glumes and lemma with some cross-veins. This pattern of convergent evolution in morphology related to a unique ecological niche warrants further study.

Difficulties in delimiting *Calamagrostis*/*Deyeuxia* and *Agrostis* from one another based on morphology in a global context have been noted (Clayton and Renvoize 1986). The same general difficulties are evident in the molecular trees. The current ITS+ETS analyses do not resolve relationships among the strongly supported *Agrostis* + *Polypogon elongatus* and *Polypogon* + *Lachnagrostis* + *A. exarata* clades and the rest of Agrostidinae. Resolution in the plastid tree is better, given the recovery of a strongly supported clade including *Agrostis*, *Polypogon*, *Calamagrostis bolanderi*, *Podagrostis aequivalvis*, *Agrostis rosei* and five species of *Calamagrostis*/*Deyeuxia* from Asia. However, the relationship of this clade to the rest of the subtribe is unresolved. Furthermore, despite sampling five plastid regions and two nrDNA regions, deep phylogenetic structure for the majority of species of *Calamagrostis*/*Deyeuxia* within Agrostidinae is lacking. Whole plastome phylogenetic analyses, including multiple species and genera of Agrostidinae, should be conducted, and may result in better resolved trees compared to few-gene plastid analyses. As no diploids are known in *Calamagrostis*/*Deyeuxia*, a possible explanation for the poor deep resolution in the current trees is that all species arose from one or more ancient hybridization events, involving species of *Agrostis* and allies or one or more extinct species ancestral to *Agrostis* and *Calamagrostis*/*Deyeuxia*. *Calamagrostis*/*Deyeuxia* may have multiple allopolyploid origins (Kellogg 2015). Low copy nuclear genes could be used to address this hypothesis, as in Wölk and Röser (2014), who identified two copy types of *topo6* in *Trisetopsis*, corresponding to two putative parental lineages of the genus. Another approach could be to produce a phylogeny with large amounts of data representing multiple independent nuclear loci generated with next-generation sequencing methods. Nicholls et al. (2015) showed how such an approach substantially improved phylogenetic resolution and support in the tropical tree genus *Inga*, for which ITS and plastid phylogenies are largely unresolved. Whatever the origin of *Calamagrostis*/*Deyeuxia*, the lack of resolution (short branch lengths) in the phylogenetic trees suggests diversification of species of *Calamagrostis*/*Deyeuxia* occurred very rapidly.

#### *Gastridium* and *Triplachne*

The genera *Gastridium* and *Triplachne* have been traditionally classified in Aveneae, Agrostidinae or Alopecurinae (Quintanar et al. 2007), and current classifications include them in Agrostidinae (Kellogg 2015, Soreng et al. 2015b). *Gastridium* comprises two species from Europe, North Africa and the middle East, characterized by having an annual habit, spikelets with or without a rachilla extension, glumes inflated around the fruit and narrowed above, then flaring distally, hardened and enlarged proximally, and awned and unawned lemmas in the same inflorescence (Kellogg 2015). *Triplachne* is monotypic; *T. nitens* (Guss.) Link is an annual species distributed in the Mediterranean characterized by having lemmas with lateral awns on either side of a central, abaxial awn (Kellogg 2015). It was described as *Agrostis nitens* Guss. and has been included in *Gastridium* (*G. nitens* (Guss.) Coss. & Durieu). Species of *Gastridium* and *Triplachne* are both diploid (2*n*=14) (Kellogg 2015). Clayton and Renvoize (1986) considered *Gastridium* and *Triplachne* to be closely related, and molecular data corroborate this hypothesis. In previous ITS trees, *Gastridium* and *Triplachne* were resolved as sister taxa (Quintanar et al. 2007, Saarela et al. 2010), as they are in the ITS tree here. These genera are allied with *Calamagrostis*/*Deyeuxia* in the ITS tree, but their precise affinities are unresolved. In plastid trees, the two genera are also sister taxa and their affinities are better resolved. In Quintanar et al. (2007), the two-taxon clade is sister to an *Agrostis* + *Chaetotropis* + *Polypogon* clade, and in Soreng et al. (2007), the clade is sister to an *Agrostis* + *Podagrostis* + *Polypogon* clade. In the *matK* tree (Suppl. material 7), *Gastridium* and *Triplachne* are a strongly supported clade and part of a broader (but poorly supported) clade including *Agrostis rosei*, *Calamagrostis bolanderi*, *Podagrostis*, some Asian species of *Deyeuxia* and an *Agrostis* + *Polypogon* clade. This better-sampled plastid topology is consistent with those recovered previously, but, as for most genera of Agrostidinae, the precise affinities of *Gastridium* and *Triplachne* remain unclear.

#### Other genera

The genera *Hypseochloa*, *Pentapogon*, *Bromidium* and *Ancistragrostis*, all classified in Agrostidinae, are not included in the current analyses. *Hypseochloa* consists of two species of annuals from Mount Cameroon and Tanzania, characterized by having spikelets with a rachilla extension and lemmas with involute margins. *Hypseochloa* is distinguished from *Agrostis* by having five-nerved glumes (one-nerved in *Agrostis*) (Clayton and Renvoize 1986, Kellogg 2015). No molecular data have been generated for *Hypseochloa*. *Pentapogon* is monotypic; *P. quadrifidus* (Labill.) Baill. is a perennial species from south eastern Australia characterized by having spikelets without a rachilla extension, glumes one-nerved and lemmas with margins convolute and covering the palea, with four apical aristae and a fifth abaxial one. Brown (2013) found multiple individuals of *P. quadrifidus* to be allied with Australasian species of *Deyeuxia* and *Dichelachne*, consistent with the current subtribal classification.

*Bromidium* includes five South American species of annuals or perennials, with lemma apices with four teeth or awns and a central awn (Rúgolo de Agrasar 1982, Kellogg 2015). The genus has been treated as Agrostis sect. Bromidium (Nees et May) Desv. No species of *Bromidium* have been included in a phylogenetic study. However, BLAST comparisons of unpublished sequences of *Bromidium tandilense* on the Barcode of Life Database (BOLD sample ID: CCDB-24954-E10) indicate the following: ITS2, 98–99% similarity with multiple *Agrostis* species, and *matK*, 100% similarity to sequences of *A. producta* (LN906638.1) and *A. elliotii* (LN906637.1), which are part of a clade with a subset of species of *Agrostis*, *Lachnagrostis* and *Polypogon* in the *matK* tree (Suppl. material 7). Further sampling of *Bromidium* is needed, particularly of *B. hygrometricum* (Nees) Nees & Meyen, the type species, which has a hairy lemma similar to species of *Lachnagrostis*, to determine its affinities and appropriate generic classification.

*Ancistragrostis* is a poorly known monotypic genus from New Guinea and Australia (Kellogg 2015). *Ancistragrostis uncinioides* S.T. Blake is a small perennial species with spikelets with a rachilla extension with long hairs, firm glumes and lemmas, glumes shorter than the floret, and lemmas awned, with the awns hooked (Blake 1946). It has been included in *Calamagrostis* (*C. uncinioides* (S.T. Blake) Reeder) (Reeder 1950) and *Deyeuxia* (*D. uncinioides* (S.T. Blake) P. Royen & Veldkamp). The hooked awn is unique in the subtribe. *Ancistragrostis* has not been studied with molecular data.

#### Torreyochloinae

Torreyochloinae includes two genera, *Amphibromus* and *Torreyochloa*, a circumscription based on plastid and nrDNA phylogenies in which they are sister taxa (Soreng and Davis 2000, Davis and Soreng 2007, Soreng et al. 2007, Saarela et al. 2010). *Amphibromus* is a genus of 12 species. One species, *A. scabrivalvis*, is native to South America and introduced in North America (Soreng and Davis 2000), and the remainder are native to Australia and New Zealand (Weiller et al. 2009). *Amphibromus* is characterized by having terminal paniculate inflorescences, spikelets laterally compressed with 2–10(–12) fertile florets, glumes rounded to slightly keeled, unawned and shorter than or subequal to the lowest lemma, lemmas two to four-toothed with teeth extending into short bristles, lemmas dorsally awned from about the middle, and calluses hairy (Watson and Dallwitz 1992 onwards, Weiller et al. 2009). Previous phylogenetic studies sampled either *A. scabrivalvis* (Davis and Soreng 2007, Soreng et al. 2007, Essi et al. 2008, Saarela et al. 2010) or *A. neesii* Steud. (Wölk and Röser 2014); none have sampled more than one species of *Amphibromus*. As such, the monophyly of *Amphibromus* has not previously been tested with molecular data. *Torreyochloa* includes three to four species native to North America and northeastern Asia (Koyama and Kawano 1964, Davis 2007), characterized by having terminal paniculate inflorescences, spikelets laterally compressed to terete with two to eight florets, glumes rounded to slightly keeled, unawned and shorter than the lowest lemma, lemmas five to seven-nerved (these prominent and scaberulous) and unawned, and calluses glabrous (Watson and Dallwitz 1992 onwards, Davis 2007). Only *T. pallida* has been included in DNA sequence-based studies; Soreng and Davis (2000) included the other North American species, *T. erecta* (Hitchc.) G.L. Church, in their combined RFLP and morphology phylogenetic analysis.

We newly sequenced seven species of *Amphibromus*, and the results are ambiguous regarding the monophyly of *Amphibromus*. In the ITS tree, which contains data for all seven species, *A. scabrivalvis* is the sister group of a weakly supported clade comprising *Torreyochloa pauciflora* and a strongly supported subclade comprising the remaining species of *Amphibromus*, including *A. neesii*, the type of the genus. We obtained ETS data for all but one (*A. scabrivalvis*) of the seven sampled species of *Amphibromus*. In the ETS tree, *Torreyochloa* is the sister group of a strongly supported clade comprising the six species of *Amphibromus*, a topology consistent with the ITS tree. In the ITS+ETS tree (*A. scabrivalvis* not sampled), *T. pallida* is sister to a robust clade of the remaining *Amphibromus* species. The plastid data support a slightly different topology, with two subclades identified: one comprises *A. scabrivalvis*, *A. recurvatus* and *T. pallida* and is weakly supported, and the other comprises the rest of the sampled species of *Amphibromus* and is moderately supported. Inclusion in phylogenetic analyses of the three unsampled species of *Torreyochloa* is needed before any taxonomic conclusions can be made about the generic circumscriptions of *Amphibromus* and *Torreyochloa*. Should it be desirable to treat all species in a single genus, the name *Amphibromus* (validly published in 1843) would have priority over *Torreyochloa* (1949).

#### Sesleriinae

Sesleriinae comprises four genera. *Sesleria* (28 perennial species), *Oreochloa* (four perennial species) and *Echinaria* (one annual species) are distributed in Europe and the Mediterranean, and are morphologically similar having condensed inflorescences with multi-flowered spikelets (Clayton et al. 2006 onwards). *Mibora* is a small genus of two annual species from Europe, north Africa and Australia with single-flowered spikelets (Clayton et al. 2006 onwards). *Sesleria*, *Oreochloa* and *Echinaria* have long been considered closely related to each other, but *Mibora* has not been considered closely related to these genera until recently (see review of classification history in Quintanar et al. 2007). Soreng et al. (2015b) included *Mibora* in Sesleriinae based on the molecular tree in Quintanar et al. (2007) in which the four genera are closely related to each other.

Inclusion of existing sequences of *Echinaria*, *Mibora*, *Oreochloa* and *Sesleria* in our analyses provides some new insight into their affinities. Even though the ITS sequences we included have all been published elsewhere (Quintanar et al. 2007, Schneider et al. 2009, Schneider et al. 2011, Minaya et al. 2013), our ITS analysis is the first one to include all four genera. Consistent with previous analyses (Quintanar et al. 2007), we find different placements for Sesleriinae in nrDNA versus plastid trees. In the ITS tree, *Mibora* and *Oreochloa* form a moderately supported clade, *Sesleria* and *Echinaria* form a strongly supported clade, and these clades form a weakly supported clade corresponding to Sesleriinae. This Sesleriinae clade is resolved as sister to *Avena*, but support for this topology is weak (<50%). In the combined ITS+ETS tree, *Sesleria* (represented by one species) is strongly supported as the sister group of Aveninae s.str., and the *Sesleria* + Aveninae s.str. clade is strongly supported as sister to Koeleriinae. Combined ITS and ETS data are needed for *Oreochloa*, *Echinaria* and *Mibora* to confirm their relationships based on nrDNA.

Considering plastid data, Sesleriinae is sampled only in the *matK* and *trnL–trnF* trees (Suppl. materials 7, 11). In these, *Echinaria*, *Mibora*, *Oreochloa* and *Sesleria* are part of Poeae chloroplast group 2. The *matK* tree includes all four genera and represents the most extensive sampling of the subtribe to date in a single phylogenetic analysis: the seven species of *Sesleria* form a clade, the two samples each of *Echinaria* and *Oreochloa* form clades, and three of the four sequences of *M. minima* form a clade. The *Mibora* sequence (KJ529357) that is not part of this clade differs from the others by several basepairs and is more similar to sequences of *Sesleria*; the reasons for this variation are unclear. Sesleriinae is not recovered as a monophyletic group in the *matK* tree. *Mibora* and *Oreochloa* form a moderately supported clade, a topology consistent with the ITS tree, and *Sesleria* is resolved as sister to *Mibora* + *Oreochloa* with low support. However, *Echinaria* falls on a relatively long branch, and is not part of the clade including the other three genera. The *trnL–trnF* tree includes all genera except *Echinaria*. A moderately supported clade of *Sesleria*, *Mibora* and *Oreochloa* is sister to Holcinae p.p. (*Holcus*, *Vahlodea*), a clade not recovered in the *matK* tree. Increased plastid sampling of each genus is needed.

Relationships among the genera of Sesleriinae based on other nuclear genes conflict, in part, with relationships based on plastid, ITS and ETS data. In a study of the phylogenetics of Pooideae based on combined nuclear regions (*Topo6*, *PhyB*, *Acc1*), Sesleriinae is not monophyletic (Hochbach et al. 2015). Instead, *Mibora* is sister to Aveninae s.l. and part of a broader clade including subtribe Parapholiinae (Poeae chloroplast group 2), whereas *Echinaria* and *Sesleria* form a clade that is sister to *Briza media* (Brizinae, Poeae chloroplast group 1), a topology that is incongruent with ITS and plastid trees. Hochbach et al. (2015) sampled only five subtribes of Poeae (Aveninae, Brizinae, Coleanthinae, Poeae, Sesleriinae), thus clarification of relationship in the tribe based on the nuclear genes they studied awaits further taxon sampling. In phylogenies of the tribe Poeae based on the nuclear gene *beta amylase*, including multiple exemplars of Aveninae s.str., Koeleriinae and Agrostidinae, *Echinaria* is part of a clade including taxa of Holcinae, Airinae and Poinae (Minaya et al. 2013, Minaya et al. 2015). This topology for *Echinaria* is discordant with the combined nuclear tree in Hochbach et al. (2015). In the combined ITS and plastid tree in Minaya et al. (2015), *Echinaria* is part of a clade including *Corynephorus*, *Deschampsia* and *Holcus. Echinaria* and all other taxa of Sesleriinae have similar affinities in the *matK* tree (Suppl. material 7), but placement of *Echinaria* in the ITS tree conflicts strongly with the combined ITS and plastid tree in Minaya et al. (2015). The ITS sequence of *Echinaria* we included was published in Minaya et al. (2015); they did not show independent ITS and plastid trees. In their combined tree, the plastid signal may have “swamped” the incongruent ITS signal. The reasons for the various gene tree conflicts in *Mibora*, *Echinaria* and *Sesleria* are unknown, but may be due to incomplete lineage sorting of one or more regions (Degnan and Rosenberg 2009). It is unclear if any of the gene trees reflect the species tree, and exploration of additional nuclear gene will likely identify more strongly supported discordant trees. Hybrid origins for *Mibora minima* (2*n*=14) and *Echinaria capitata* are not likely explanations for conflicting gene trees in these species because both are diploid (Ortiz et al. 1999, Rice et al. 2015). There is, however, considerable polyploidy in the more diverse *Sesleria*, some of which has been attributed to autopolyploidy (Kuzmanović et al. 2013).

#### Holcinae p.p. (*Deschampsia*, Deyeuxia sect. Stylagrostis and *Scribneria*)

An unexpected result reported here is the placement of several South American species of *Calamagrostis*/*Deyeuxia* in a clade with species of *Deschampsia* (Holcinae), a polyploid (2*n*=26, 56) genus of 30–40 species distributed in temperate regions of the northern and southern hemispheres (Clayton et al. 2006 onwards, Chiapella and Zuloaga 2010, Kellogg 2015). A few species traditionally recognized in *Deschampsia* are now treated in *Avenella* and *Vahlodea* based on molecular data (Chiapella 2007). In the ITS+ETS tree, *Deyeuxia chrysantha* J. Presl, *D. eminens* J. Presl, *D. ovata* J. Presl, *D. hackelii* (Lillo) Parodi, *D. aurea* Munro ex Wedd., *D. podophora* (Pilg.) Sodiro and *D. ligulata* Kunth are part of a strongly supported clade including all sampled species of *Deschampsia*: *D. cespitosa* (L.) P. Beauv., *D. elongata* (Hook.) Munro and *D. brevifolia* R. Br. These same species of *Deyeuxia* are similarly allied with the multiple species of *Deschampsia* in the better-sampled ITS tree. In the ITS tree, *D. chrysantha*, *D. eminens* and *D. ovata* are part of weakly supported clade including one newly sequenced sample of *D. cespitosa* from South America, whereas the affinities of *D. hackelii*, *D. aurea*, *D. podophora*, *D. ligulata* and most other *Deschampsia* species are unresolved. Similarly, all sampled species of *Deschampsia* and the seven species of *Deyeuxia* species form a strongly supported clade in the plastid tree. There is little plastid variation among these species, and thus little phylogenetic structure in the clade. The lack of plastid variation observed here is consistent with earlier work that sampled plastid data for most species of *Deschampsia* (Chiapella 2007, Chiapella et al. 2011).

The genus *Stylagrostis* Mez (Mez 1922) was proposed to accommodate a group of 14 South American species with stipitate florets (i.e., the rachilla below the single floret is slightly elongated, raising it above the glumes) (Escalona 1988a), including the species of *Calamagrostis*/*Deyeuxia* that are part of the *Deschampsia* clade. These species are distributed in the paramo and puna in South America, and were originally described in *Agrostis*, *Calamagrostis* and *Deyeuxia*. We are not aware of chromosome counts for any of them. Subsequent authors did not recognize *Stylagrostis* at genus rank. Escalona (1988a) included the species of *Stylagrostis* in Calamagrostis sect. Deyeuxia subsect. Stylagrostis (Mez) Escalona. Rúgolo de Agrasar and Villavicencio L. (1995) included them in Deyeuxia sect. Stylagrostis, the name we use in the trees here. In a revision of Calamagrostis subsect. Stylagrostis, Escalona (1988b) recognized 14 species: *C. amoena* (Pilg.) Pilg. [=*D. amoena* Pilg.], *C. ampliflora* Tovar, *C. aurea* (Munro ex Wedd) Hack. [=*D. aurea* Munro ex Wedd.], *C. chaseae* Luces, *C. chrysantha* [=*D. chrysantha* J. Presl], *C. cleefii* Escalona, *C. curta* (Wedd.) Hitchc. [=*D. curta* Wedd.], *C. guamenensis* Escalona, *C. eminens* (J. Presl) Steud. [=*D. eminens* J. Presl], *C. ligulata* (Kunth) Hitchc. [=*D. ligulata* Kunth], *C. mollis* Pilg., *C. ovata* (J. Presl) Steud. [=*D. ovata* J. Presl], *C. ramonae* Escalona and *C. pisinna*. There are a few taxonomic differences between the treatment of Escalona (1988b) and ones used to identify material we sequenced. Escalona (1988b) treated *C. podophora* as a synonym of *C. ligulata*, whereas we sampled material identified as both species. Our results indicate these taxa are closely related. Escalona (1988b) included *D. hackelii*, from Argentina, in her cluster analyses, but did not include it in her taxonomic treatment, although she noted *C. hackelii* resembles *C. ovata*.

In a cladistic analysis of morphological variation in Calamagrostis subsect. Stylagrostis, Escalona (1988b) identified two main clades, one comprising *C. ovata*, *C. chrysantha*, *C. aurea* and *C. eminens* and the other comprising the remaining nine species. The first clade was defined by having ligules membranous and elongate (6–15 mm long), tapering or bifurcate, fertile lemma surfaces glabrous and lemma awns twisted. The second clade was defined by having ligules membranous and truncate (1–3 mm long), fertile lemma surfaces scabrous and lemma awns straight. Escalona (1988b) noted species of the first group share large glumes, sometimes two florets (e.g., *C. aurea*, *C. eminens*) and golden ovate, oblong or open shining inflorescences, while species of the second group share open or ovate purple inflorescences, twisted awns, soft or liquid endosperm and flat or involute leaves.

Our sampling mostly includes taxa from the first clade identified by Escalona (1988b), including *C. ovata*, *C. chrysantha*, *C. aurea*, *C. eminens*, *C. hackelii* and *C. ligulata*; all of these are part of the *Deschampsia* clade. Of the second clade identified by Escalona (1988b), we obtained plastid data for only *C. pisinna*, which is moderately supported as the sister group of Aveninae s.l. The taxon is not closely related to the other sampled species of Deyeuxia sect. Stylagrostis or other taxa of Agrostidinae in the plastid tree, and represents a previously unknown lineage. Placement outside the *Deschampsia* clade points towards multiple origins of the stipitate floret used to define Deyeuxia sect. Stylagrostis. Sampling is needed of the outstanding species of Deyeuxia sect. Stylagrostis.

Similarity between some species of Deyeuxia sect. Stylagrostis and *Deschampsia* has been noted previously. Parodi (1949a) observed that *Deyeuxia eminens* and related species common in the high Andes from Mendoza to Ecuador have lax panicles, denticulate lemmas and chestnut or golden spikelets similar to species of *Deschampsia*, differing only by having one-flowered spikelets (vs. two-flowered, rarely one- or three-flowered in *Deschampsia*; Chiapella and Zuloaga (2010)) and with the rachilla prolonged and hairy, usually with a rudiment of a second floret. The molecular data confirm the insights of Parodi (1949a) from more than sixty years ago. Aside from comment in Rúgolo de Agrasar (1978) acknowledging the ideas in Parodi (1949a), we are not aware of other indication in the literature of a possible close relationship between Deyeuxia sect. Stylagrostis and *Deschampsia*. However, two of us (PMP and RJS) noted similarities between *Deyeuxia eminens* and *Deschampsia* while in the field in Chile, and could not determine to which genus the specimens belonged. The collections had mixes of one- and some two-flowered spikelets, but otherwise looked like *Deschampsia cespitosa* s.l.; these collections were confirmed as *D. eminens* by O. Matthei.

The only detailed morphological description of Deyeuxia sect. Stylagrostis (Escalona 1988a, b) includes characteristics of multiple species not yet sampled and whose generic affinities are unknown. Comparing that description with current ones of *Deschampsia* to characterize similarities and differences beyond those noted by Parodi (1949a) is therefore not possible. Nevertheless, a few similarities and differences between the closely related species of Deyeuxia sect. Stylagrostis and *Deschampsia* are apparent. Chiapella and Zuloaga (2010) recognized 15 South American species of *Deschampsia*, and described the genus (excluding *Avenella* and *Vahlodea*) as having ligules acute, 5–10(–12) mm long, and rachillas pubescent and prolonged beyond the upper floret. The species of Deyeuxia sect. Stylagrostis that are part of the *Deschampsia* clade similarly have long ligules and extended rachillas. These species differ from *Deschampsia* s.str. by having stipitate florets (vs. sessile, as recorded for *Deschampsia* by Clayton and Renvoize (1986)) and one (rarely two) florets per spikelet. A distinctly elongated rachilla internode above the glumes is also present in *Holcus* (Watson and Dallwitz 1992 onwards), which is not closely related to *Deschampsia*, even though they are classified in the same subtribe, Holcinae.

Because the plastid and nrDNA trees indicate that several species of Deyeuxia sect. Stylagrostis and *Deschampsia* arose from the same common ancestor, and there is no evidence that the species of Deyeuxia sect. Stylagrostis are more closely related to one another than to all or a subset of the species of *Deschampsia*, continued recognition of *Deschampsia* in its current sense (e.g., Chiapella and Zuloaga 2010) would render the genus paraphyletic. It is therefore proposed that the seven species of Deyeuxia subsect. Stylagrostis we demonstrate to be part of the *Deschampsia* clade be transferred to *Deschampsia*, and the needed combinations are made here (see Taxonomy). Further study is needed to better characterize *Deschampsia* in light of the additional morphological diversity we are including in the genus, and to identify non-molecular synapomorphies for the genus. The lineage may also include other as-yet unsampled taxa that have been treated in Deyeuxia sect. Stylagrostis. At present there is insufficient data to support a subdivisional classification of *Deschampsia*.

The monotypic genus *Scribneria* (*S. bolanderi* (Thurb.) Hack.) was recently found to be closely related to *Deschampsia*, but was variously placed in earlier classifications. Clayton and Renvoize (1986) classified *Scribneria* with the genera *Agropyropsis* (Batt. & Trab.) A. Camus, *Hainardia*, *Narduroides* Rouy, *Parapholis* and *Pholiurus* Trin. in the subtribe Hainardieae, based on inflorescence characteristics (Soreng et al. 2007). Molecular data do not support Hainardieae as a natural group, and of the six genera traditionally recognized in the subtribe, only *Parapholis* and *Hainardia* are closely related (Schneider et al. 2009). *Scribneria* was later placed among Aveneae taxa in a study based on plastid restriction site data (Soreng and Davis 2000). It was not allied with *Deschampsia* there, however, because the *Deschampsia* sample was a misidentified species of *Agrostis* (Davis and Soreng 2007). Based on the restriction site data, *Scribneria* was treated in its own subtribe Scribneriinae Soreng & J.I. Davis (Soreng et al. 2003, Soreng et al. 2007). *Scribneria bolanderi* was only recently included in a sequence-based (ITS, *matK*, 3’-*trnK*) molecular study, and was resolved as sister to the one sampled species of *Deschampsia* (Schneider et al. 2012). Based on these results, Schneider et al. (2012) argued placement of *Scribneria* in its own subtribe is not necessary and the genus could be accommodated in a subtribe with *Deschampsia*. This was followed by Soreng et al. (2015b), in which *Deschampsia* (including *Scribneria*), *Holcus* and *Vahlodea* were classified in subtribe Holcinae. In a parallel classification of grasses, Kellogg (2015) placed *Scribneria* in Airinae, and treated Holcinae as a synonym of Airinae.

We included the previously published ITS and *matK* (Suppl. material 7) sequences of *Scribneria bolanderi* in our analyses, and find that *Scribneria* is nested within *Deschampsia*. Soreng et al. (2015b) treated *Scribneria* as a synonym of *Deschampsia*, based on results of preliminary analyses of the current data set. As the needed combination has not yet been made, *Scribneria bolanderi* is here transferred to *Deschampsia* (see Taxonomy). *Scribneria bolanderi* is similar to *Deschampsia* s.str. in having an awned lemma, a hairy callus, punctiform hilums and a ploidy level of 2*n*=26. It differs from *Deschampsia* s.str. by several characteristics, including one-flowered spikelets (vs. (1)2(3)-flowered), spikelets distichous (vs. not distichous), usually spicate to racemose inflorescences (sometimes reduced panicles) (vs. paniculate), tough inflorescence rachises (vs. fragile), and fertile spikelets sessile (vs. pedicellate) (Clayton et al. 2006 onwards, Schneider et al. 2009, Chiapella and Zuloaga 2010, Worley 2016).

Holcinae in its current circumscription, including *Holcus*, *Vahlodea* and *Deschampsia* (Soreng et al. 2015b), is not monophyletic because *Holcus* and *Vahlodea* do not form a clade with *Deschampsia* here or in other plastid and nrDNA trees (Quintanar et al. 2007, Saarela et al. 2010, Grass Phylogeny Working Group II 2012, Persson and Rydin 2016). Given this, *Deschampsia* (newly including *Scribneria bolanderi* and multiple species of Deyeuxia sect. Stylagrostis) would be better treated in its own monotypic subtribe, *Aristaveninae* F. Albers & Butzin (type *Aristavena setacea* (Huds.) F. Albers & Butzin, a synonym of *Deschampsia setacea* (Huds.) Hack.), and Holcinae restricted to *Holcus* and *Vahlodea*. Some recent authors, such as Valdés and Scholz (2006) in the Euro+Med Checklist, recognize the monotypic genus *Aristavena* F. Albers & Butzin as distinct from *Deschampsia*. Such a classification is inconsistent with molecular data because *A. setacea* is part of the *Deschampsia* clade in the ITS tree. Recognizing *Aristavena* as a distinct genus renders *Deschampsia* paraphyletic.

### Taxonomy

#### Calamagrostis × calammophila

Taxon classificationPlantaePoalesPoaceae

Saarela nom. nov.

urn:lsid:ipni.org:names:77165976-1

##### Blocking name.

*Calamagrostis baltica* Trin. **Basionym**: *Arundo baltica* Flüggé ex Schrad., Fl. Germ. 223, t. 5, f. 3. 1806. ×*Ammocalamagrostis baltica* (Flüggé ex Schrad.) P. Fourn., Monde Pl., Rev. Mens. Bot. 35: 28. 1934. ×*Calammophila baltica* (Flüggé ex Schrad.) Brand, Syn. Deut. Schweiz. Fl. (ed. 3) 3: 2715. 1907. *Ammophila baltica* (Flüggé ex Schrad.) Link, Hort. Berol. 1: 105. 1827. Type: Germany: *litoribus maris baltici prope Svienemunde*, *Fleugge* s.n. (syntypes: B [B -W 02259 -01 0, B -W 02259 -02 0, B -W 02259 -03 0]). The new name reflects the origin of the hybrid taxon, involving a species of *Calamagrostis* (*C. epigeios*) and a species formerly recognized in the genus *Ammophila* (*A. arenaria*).

#### Calamagrostis breviligulata

Taxon classificationPlantaePoalesPoaceae

(Fernald) Saarela comb. nov.

urn:lsid:ipni.org:names:77165977-1

##### Basionym.

*Ammophila breviligulata* Fernald, Rhodora 22(256): 71. 1920. Type: USA: Connecticut, Milford, 27 Aug 1902, *C.H. Bissell* s.n. (holotype: GH! [GH00023024]; isotype: US! [US-863726 barcode 00478957).

#### Calamagrostis breviligulata subsp. champlainensis

Taxon classificationPlantaePoalesPoaceae

(F. Seym.) Saarela comb. nov.

urn:lsid:ipni.org:names:77165978-1

##### Basionym.

*Ammophila champlainensis* F. Seym., Sida 2(5): 349–350, f. 3–4. 1966. Type: USA: New York, on Lake Champlain, Au Sable Point, in sand, 3 July 1902, *N.F. Flynn* s.n. (lectotype: VT! [UVMVT015687], designated by Delisle-Oldham et al. (2008: 139), corrected from “holotype”; isotypes VT! [UVMVT015688], DUKE! [DUKE10000234]). Of the two sheets of *Flynn* s.n. at VT, Delisle-Oldham et al. (2008: 139) “considered [the specimen with the word Type written on the sheet] to be the holotype.” We consider this equivalent to the phrase “designated here”, as required by the Code for designation of a lectotype after 1 January 2001 (Art. 7.10), and correct “holotype” to “lectotype.”

#### Calamagrostis × don-hensonii

Taxon classificationPlantaePoalesPoaceae

(Reznicek & Judz.) Saarela comb. nov.

urn:lsid:ipni.org:names:77165979-1

##### Basionym.

×*Calammophila don-hensonii* Reznicek & Judz., Michigan Bot. 35: 36. 1996. Type: USA: Michigan, Alger Co., Grand Island, Williams Landing, along shore in section 22, T47N, R19W, south shore of Island ca. 5 1/4 km NW of Munising, 9 Jul 1991, *Reznicek*, *Henson*, *Henson & D. Tiller 8827* (holotype MICH! [1108624], isotype US! [US-3537125 barcode 00955513]).

#### Deschampsia aurea

Taxon classificationPlantaePoalesPoaceae

(Munro ex Wedd.) Saarela comb. nov.

urn:lsid:ipni.org:names:77165980-1

##### Basionym.

*Deyeuxia aurea* Munro ex Wedd., Bulletin de la Société Botanique de France 22: 176 (err. typ. 156), 179. 1875. *Calamagrostis aurea* (Munro ex Wedd.) Hack. ex Sodiro, Anales Univ. Centr. Ecuador, 3(25): 481. 1889. Type: Ecuador: Andes de Quito, 1859, *Jameson* s.n. (syntypes: BM! [BM000938555], C! [C10016868], S! [S-R-1454], K! [K000308462, K000308463], GOET [GOET006117], LE! [LE00009397], NY! [00380534], P! [P00729794], US! [US-844970 barcode 00406340, US-844971 barcode 00406339, US-844972 barcode 00149267], W! [W18860008092, W18890241742, W18890028043, W18860008093]). The protologue of the basionym states only “Equateur (Jameson)”, and as such there is no holotype or isotypes, despite the interpretations of some authors in the literature (Escalona 1988b, Soreng and Greene 2003, Vega and de Agrasar 2013) and annotations on herbarium specimens. The name has probably been inadvertently lectotypified, but we have not tracked this.

#### Deschampsia hackelii

Taxon classificationPlantaePoalesPoaceae

(Lillo) Saarela comb. nov.

urn:lsid:ipni.org:names:77165981-1

##### Basionym.

*Calamagrostis hackelii* Lillo, Anales Mus. Nac. Buenos Aires 21: 100, t. 4, f. A. 1–5. 1911. *Deyeuxia hackelii* (Lillo) Parodi, Revista Argentina de Agronomía 20(1): 14. 1953. Type: Argentina: Tucumán, Tafí, Cumbres Calchaquíes, 4400 m, 2 Feb 1907, *M. Lillo 5602* (syntypes US! [US-3099597 barcode 00406323], W! [W19160037761], BAA! [BAA00000078], CORD! [CORD00001544]. The protologue cites a gathering but not a specimen, thus there is no holotype or isotypes, despite the interpretations of some authors.

#### Deschampsia ovata

Taxon classificationPlantaePoalesPoaceae

(J. Presl) Saarela comb. nov.

urn:lsid:ipni.org:names:77165982-1

##### Basionym.

*Deyeuxia ovata* J. Presl, Reliquiae Haenkeanae 1(4–5): 246. 1830. *Calamagrostis ovata* (J. Presl) Steud., Nomencl. Bot. (ed. 2) 1: 251. 1840. *Stylagrostis ovata* (J. Presl) Mez, Bot. Arch. 1(1): 20. 1922. Type: Peru: in montanis Peruviae huanoccensibus, *Haenke* s.n. (syntypes: BR! [0000006865689], HAL! [HAL0107127], PR, PRC, US! [US-3099580 barcode 00406354 (fragm.)], W! [W18890241741, W-0009755). The protologue cites a gathering but not a specimen, thus there is no holotype or isotypes, despite the interpretations of some authors.

#### Deschampsia ovata var. nivalis

Taxon classificationPlantaePoalesPoaceae

(Wedd.) Saarela comb. nov.

urn:lsid:ipni.org:names:77165983-1

##### Basionym.

*Deyeuxia nivalis* Wedd., Bull. Soc. Bot. France 22: 176 (err. type. 156),180. 1875. *Calamagrostis nivalis* (Wedd.) Hack. ex Buchtien, Contr. Fl. Bolivia 1: 75. 1910. *Stylagrostis nivalis* (Wedd.) Mez, Bot. Arch. 1(1): 20. 1922. Deyeuxia ovata var. nivalis (Wedd.) Villav., Rev. *Deyeuxia* Bolivien 75, f. 18D, 20. 1995. Calamagrostis ovata var. nivalis (Wedd.) Soreng, Contr. U.S. Natl. Herb. 48: 213. 2003. Type: Bolivia: *d’Orbigny 110* (lectotype P! [P00729773], designated by Rúgolo in Villavicencio (1995), isolectotypes S! [S-10-26721, S-R-7643!], BAA! [BAA00001854], W! [W18890120024]).

#### Deschampsia chrysantha

Taxon classificationPlantaePoalesPoaceae

(J. Presl) Saarela comb. nov.

urn:lsid:ipni.org:names:77165984-1

##### Basionym.

*Deyeuxia chrysantha* J. Presl, Reliquiae Haenkeanae 1(4–5): 247. 1830. *Calamagrostis chrysantha* (J. Presl) Steud., Nomencl. Bot. (ed. 2) 1: 250. 1840. *Stylagrostis chrysantha* (J. Presl) Mez, Bot. Arch. 1(1): 20. 1922. Type: *Peruviae montanis huanoccensibus*, *Haenke* s.n. (lectotype PR!, designated by Villavicencio (1995), isolectotypes PR!, PRC! [PRC450186], US! fragm.).

#### Deschampsia chrysantha var. phalaroides

Taxon classificationPlantaePoalesPoaceae

(Wedd.) Saarela comb. nov.

urn:lsid:ipni.org:names:77165985-1

##### Basionym.

*Deyeuxia phalaroides* Wedd., Bull. Soc. Bot. France 22: 177, 180. 1975. Deyeuxia chrysantha var. phalaroides (Wedd.), Rev. *Deyeuxia* Bolivien 68, f. 14D–C, 16. 1995. Calamagrostis chrysantha var. phalaroides (Wedd.) Soreng, Contr. U.S. Natl. Herb. 48: 198. 2003. *Stylagrostis phalaroides* (Wedd.) Mez, Bot. Arch. 1(1): 20. 1922. Type: Bolivia: *Viciniis* La Paz, *via ad Coroico, in locis frigidis*, Reg. Alp. 5000 m, April 1857, *G. Mandon 1319* (lectotype: P! [P00740413], [first-step] lectotype designated by Rúgolo de Agrasar (2006: 163), [second-step] lectotype **designated here**; isolectotypes: BM! [BM000938556], GH! [00023430], GOET! [GOET006110], NY! [NY00380546], P! [P00740414, P00740374, P00729809], S! [S-R-829], US! [US-863443 barcode 00170221 (fragm.), US-3099579 barcode 00170222, US-1126796 barcode 00479095], W[W18890028049, W0009753]). Rúgolo de Agrasar (2006) designated a specimen at P as lectotype, but she did not indicate which of the three sheets is the lectotype. We thus designate a second-step lectotype.

#### Deschampsia eminens

Taxon classificationPlantaePoalesPoaceae

(J. Presl) Saarela comb. nov.

urn:lsid:ipni.org:names:77165986-1

##### Basionym.

*Deyeuxia eminens* J. Presl, Reliquiae Haenkeanae 1(4–5): 250. 1830. *Calamagrostis eminens* (J. Presl) Steud., Nomencl. Bot. (ed. 2) 1: 250. 1840. *Agrostis eminens* (J. Presl) Griseb., Abh. Königl. Ges. Wiss. Göttingen 19: 254. 1874. *Stylagrostis eminens* (J. Presl) Mez, Bot. Arch. 1(1): 20. 1922. Type: Peru: Huánuco, *hab. in Peruviae montanis huanoccensibus*, *T. Haenke* s.n. (syntypes: HAL! [HAL-107170 barcode HAL0107170], W! [W0009759], US! [US-81862 barcode 00149262 (fragm.)], PRC! [PRC-629 barcode PRC450192]. No specimen is indicated in the protologue, thus there is no holotype, despite the interpretations of some authors (e.g., Rúgolo de Agrasar 2006).

#### Deschampsia eminens var. fulva

Taxon classificationPlantaePoalesPoaceae

(Griseb.) Saarela comb. nov.

urn:lsid:ipni.org:names:77165987-1

##### Basionym.

*Agrostis fulva* Griseb., Abh. Königl. Ges. Wiss. Göttingen 24: 294. 1879. *Calamagrostis fulva* (Griseb.) Kuntze, Revis. Gen. Pl. 3(3): 344. 1898. *Deyeuxia fulva* (Griseb.) Parodi, Revista Argent. Agron. 20(1): 14. 1953. Deyeuxia eminens var. fulva (Griseb.) Rúgolo, Boletin de la Sociedad Argentina de Botanica 30(1–2): 112. 1994. Calamagrostis eminens var. fulva (Griseb.) Soreng, Contr. U.S. Natl. Herb. 48: 201. 2003. *Stylagrostis fulva* (Griseb.) Mez, Bot. Arch. 1(1): 20. 1922. Type: Argentina. Salta, Nevado del Castillo, 19-23 Mar 1873, *G. Hieronymus & P.G. Lorentz 77* (syntypes: BAA! [BAA00001340, BAA00001341, BAA00001342], CORD! [CORD00004691, CORD00004690, CORD00004692], GOET! [GOET006218, GOET006219], K [K000308483, K000308482], S! [S-R-816], US! [US-732872 barcode 00406313, US-1126837 (ex W) barcode 00406314, US-76271! barcode 00156429 (fragm. ex B)], W! [W19160037768, W19160037767]. No specimen is indicated in the protologue, thus there is no holotype.

#### Deschampsia eminens var. inclusa

Taxon classificationPlantaePoalesPoaceae

(Rúgolo) Saarela comb. nov.

urn:lsid:ipni.org:names:77165988-1

##### Basionym.

Deyeuxia eminens var. inclusa Rúgolo, Darwiniana 44(1): 195, f. 24. 2006. Type: Argentina. San Juan: Dpto. Iglesia, Qda. del Agua Negra, 3750 m., 21 Feb 1979, *Cabrera 30062* (holotype: SI).

#### Deschampsia parodiana

Taxon classificationPlantaePoalesPoaceae

(Kunth) Saarela nom. nov.

urn:lsid:ipni.org:names:77165989-1

##### Basionym.

*Deyeuxia ligulata* Kunth, Nova Genera et Species Plantarum (quarto ed.) 1: 145. 1815[1816]. *Arundo ligulata* (Kunth) Poir., Encycl. 4: 706. 1816. *Calamagrostis ligulata* (Kunth) Hitchc., Contr. U.S. Natl. Herb. 24(8): 372. 1927, non Deschampsia ligulata (Stapf) Henrard, Blumea 1(2): 309. 1935. Type: Ecuador: Pichincha: Montis Javeral, 2750 m, Jan, *Humboldt & Bonpland 60* (syntypes: P! [P026295, P00129584], US! [US-3049486 barcode 00479089 (fragm.)]). No specimen is indicated in the protologue, thus there is no holotype. The epithet commemorates Lorenzo Raimundo Parodi (1895-1966), who recognized similarities among some species of Deyeuxia sect. Stylagrostis and Deschampsia.

#### Deschampsia podophora

Taxon classificationPlantaePoalesPoaceae

(Pilg.) Saarela comb. nov.

urn:lsid:ipni.org:names:77165990-1

##### Basionym.

*Calamagrostis podophora* Pilg., Bot. Jahrb. Syst. 42 (1): 66. 1908. *Deyeuxia podophora* (Pilg.) Sodiro, Revista del Colégio Nacional Vicente Rocafuerte 12: 79. 1930. Type: Peru. Junín, Berge weslich von Huacapistana, 3500 m, 18 Jan 1903, *A. Weberbauer 2231* (lectotype: BAA! [BAA-4647 barcode BAA00000767 (fragm. ex B), designated by Vega and de Agrasar (2013); isolectotype US! [US-2947284 barcode 00149282].

#### Deschampsia bolanderi

Taxon classificationPlantaePoalesPoaceae

(Thurb.) Saarela comb. nov.

urn:lsid:ipni.org:names:77165991-1

##### Basionym.

*Lepturus bolanderi* Thurb., Proc. Amer. Acad. Arts 7: 401. 1868. *Scribneria bolanderi* (Thurb.) Hack., Bot. Gaz. 11(5): 105. 1886. Type: USA. California: dry gravelly soil, Russian River Valley, 1866, *Bolander 4669* (syntypes: UC! [UC-39830], MO! [MO-1837546 barcode MO-2151592, MO-1837547 barcode MO-2151593] NDG! [NDG-36442 barcode NDG08312], GH! [GH00361145], NY! [NY00381289, NY00381288], YU! [YU244787], W! [W18890217339]). The protologue of the basionym cites a gathering but not a specimen, thus there is no holotype, despite the interpretations of some authors.

#### Subtribe Lagurinae

Taxon classificationPlantaePoalesPoaceae

Saarela subtrib. nov.

urn:lsid:ipni.org:names:77165992-1

##### Type genus.

*Lagurus* L., Sp. Pl. 1: 81. 1753. Differs from Aveninae s.str. and Koeleriinae in having glumes covered with woolly hairs, and their apices acuminate, awned and the awns covered with hairs. Includes only *Lagurus ovatus* L.

## Supplementary Material

XML Treatment for Calamagrostis × calammophila

XML Treatment for Calamagrostis breviligulata

XML Treatment for Calamagrostis breviligulata subsp. champlainensis

XML Treatment for Calamagrostis × don-hensonii

XML Treatment for Deschampsia aurea

XML Treatment for Deschampsia hackelii

XML Treatment for Deschampsia ovata

XML Treatment for Deschampsia ovata var. nivalis

XML Treatment for Deschampsia chrysantha

XML Treatment for Deschampsia chrysantha var. phalaroides

XML Treatment for Deschampsia eminens

XML Treatment for Deschampsia eminens var. fulva

XML Treatment for Deschampsia eminens var. inclusa

XML Treatment for Deschampsia parodiana

XML Treatment for Deschampsia podophora

XML Treatment for Deschampsia bolanderi

XML Treatment for Lagurinae

## Appendix 1

**Voucher information and GenBank accession numbers for new DNA sequence data and a subset of previously published sequences.** Information is presented in the following order: **taxon, provenance, voucher, *trnL–trnF*, *psbK–psbI*, *psbA–rps19–trnH*, *atpF-atpH*, *matK*, *ITS*, *ETS***. Additional voucher information is given in Suppl. material 1. Accession numbers for newly generated sequences begin with KX and MF; others are previously published. A dash indicates no sequence was obtained. The majority of the previously published sequences of ITS, *matK* and *trnL–trnF* are reported in Suppl. material 2. The superscripted numbers refer to inversions and indels in a subset of the regions, as defined at the end of the appendix.

***Agrostis breviculmis*** Hitchc., PERU, Ancash, *Peterson & Soreng 21841* (US), –, KX873575^6^, KX873968, –, –, –, –; Lima, *Peterson et al. 20294* (US), KX872590, KX873555^6^, KX873947, KX871908, KX873188, KX872897, KX872263^9,14^; Ancash, *Peterson & Refulio 17933* (US-3481510), –, KX873556^6^, KX873948, KX871909, KX873189, KX872898, KX872264^9,14^. ***Agrostis capillaris*** L., USA: California, *Peterson et al. 19798* (CAN-597515), KX872591, KX873579^6^, KX873972, KX871932, KX873190, KX872899, KX872282^14^ ; Oregon, *Saarela & Roe 249* (CAN-590286), KX872592, KX873582^6^, KX873975, KX871935, KX873191, FJ377621, KX872285^14^; CANADA: British Columbia, *Saarela 748* (CAN-591518), KX872593, KX873557^6^, KX873949, KX871910, KX873192, KX872900, KX872265^14^. ***Agrostis exarata*** Trin., USA: California, *Peterson et al. 19730* (CAN-593975), KX872594, KX873558^6^, KX873950, KX871912, KX873193, KX872901, KX872266^14^; CANADA: British Columbia, *Saarela 755* (CAN-591521), KX872595, KX873559^6^, KX873951, KX871911, KX873194, KX872902, KX872267^14^. ***Agrostis foliata*** Hook. f., PERU: Ancash, *Peterson et al. 21732* (US), –, –, –, –, –, KX872903, –; ***Agrostis gelida*** Trin., ECUADOR: Azuay, *Peterson et al. 8862* (US-3237108), KX872596, KX873560^6^, KX873952, KX871913, KX873195, KX872904, KX872268^9,14^. ***Agrostis gigantea*** Roth, USA: California, *Peterson et al. 19724* (CAN-593954), KX872597, KX873561^6^, KX873953, KX871914, KX873196, KX872905, KX872269^14^; KYRGYZ REPUBLIC: Chu, *Soreng et al. 7550* (US), –, KX873562^2,6^, KX873954, KX871915, KX873197, –, –; CANADA: Yukon, *Peterson et al.* 18662 (CAN-590999), –, KX873564^6^, KX873956, KX871917, –, –, –. ***Agrostis hallii*** Vasey, USA: California, *Peterson et al. 19699* (CAN-593958), KX872598, KX873566^6^, KX873958, KX871919, KX873199, KX872907, KX872272^9,14^. ***Agrostis imberbis*** Phil., CHILE: Region II (Antofagasta), *Soreng & Soreng 7218* (US), GQ266674, KX873567^6^, KX873959, KX871920, KX873200, FJ377618, KX872273^9,14^. ***Agrostis mertensii*** Trin., USA: New Hampshire, *Peterson & Saarela 20895* (CAN-602603), KX872599, KX873568^6^, KX873960, KX871921, KX873201, FJ377620, KX872274^9,14^; New Hampshire, *Peterson & Saarela 20884* (CAN-602606), KX872600, KX873569^6^, KX873961, KX871922, KX873202, KX872908, KX872275^9,14^. ***Agrostis meyenii*** Trin., CHILE: Region IX (Araucania), *Soreng & Soreng 7209* (US), FJ394555, KX873570^6^, KX873962, KX871923, KX873203, FJ377619, KX872276^9,14^. ***Agrostis rosei*** Scribn. & Merr., MEXICO: Durango, *Peterson & Saarela 21269* (US), KX872601, –, KX873963, KX871924, KX873204, MF348276, –. ***Agrostis scabra*** Willd., CANADA: Yukon, *Peterson et al. 18491* (CAN-591053), KX872604, KX873577^6^, KX873970, KX871930, KX873207, KX872909, KX872281^9,14^; Alberta, *Saarela et al. 272* (CAN), –, KX873578^6^, KX873971, KX871931, KX873208, –, –. ***Agrostis stolonifera*** L., KYRGYZ REPUBLIC: Issyk-Kul, *Soreng et al. 7582* (US-3500019), KX872605, KX873580^6^, KX873973, KX871933, KX873209, KX872910, KX872283^9,14^; CANADA: Alberta, *Peterson et al. 18382* (CAN-591041), KX872606, KX873581^6^, KX873974, KX871934, –, FJ377622, KX872284^9,14^; Alberta, *Saarela 751* (CAN-591522), KX872607, KX873563^6^, KX873955, KX871916, KX873210, KX872911, KX872270^9,14^. ***Agrostis tolucensis*** Kunth, MEXICO: Mexico, *Peterson et al. 21338* (CAN-602178), KX872608, KX873583^6^, KX873976, KX871936, KX873211, MF348277, KX872286^9,14^; PERU: Ancash, *Peterson et al. 21487* (US), KX872609, KX873573^6^, KX873966, KX871927, –, KX872912, KX872279^9,14^; Ancash, *Peterson et al. 21523* (US), –, KX873574^6^, KX873967, KX871928, –, –, –; Ancash, *Peterson et al. 21670* (US), KX872610, KX873576^6^, KX873969, KX871929, –, KX872913, KX872280^9,14^; Lima, *Peterson et al. 20271* (US), KX872611, KX873565^6^, KX873957, KX871918, KX873198, KX872906, KX872271^9,14^.

***Alopecurus aequalis*** Sobol. var. ***aequalis***, USA: California, *Peterson et al. 19710* (US-3539851), –, KX873585, KX873978, KX871938^8^, KX873213, –, –. ***Alopecurus magellanicus*** Melgar, PERU: Puno, *Peterson et al. 20626* (US), KX872612, KX873584, KX873977, KX871937^8^, KX873212, KX872914, KX872287^15^.

***Ammophila arenaria*** (L.) Link, POLAND: Pomorskie Voivodeship, *Paszko* s.n., sample *S-7* (KRAM-558959), KX872613, KX873586, KX873979, KX871939, KX873214, KX872915, KX872288^14^; Pomorskie Voivodeship, *Paszko* s.n., sample J6 (KRAM-558958), –, KX873587, KX873980, KX871940, KX873215, –, –; USA: California, *Peterson et al. 19705* (CAN-593907), KX872614, KX873588, KX873981, KX871941, KX873216, KX872916, KX872289^14^. ***Ammophila breviligulata*** Fernald, USA: New York, *Peterson & Saarela 20867* (CAN-602599), KX872615, KX873589, KX873982, KX871942, KX873217, FJ377623, KX872290^14^. CANADA: British Columbia, *Page* s.n. (UBC), KX872616, KX873590, KX873983, KX871943, KX873218, KX872917, KX872291^14^.

***Amphibromus fluitans*** Kirk, AUSTRALIA: Victoria, *Beauglehole 82449* (MELU-224198), –, –, –, –, –, MF348278, KX872292^15^. Victoria, *Ashton* s.n. (MELU-2063896), KX872617, KX873591, KX873984, KX871944, KX873219, KX872918, –. ***Amphibromus macrorhinus*** S.W.L. Jacobs & Lapinpuro, AUSTRALIA: Victoria, *Clarke 2625* (MELU-2029318), KX872618, KX873592, KX873985, KX871945, KX873220, KX872919, KX872293^15^. AUSTRALIA: Victoria, *Thomas 586* (MELU-2025702), KX872619, KX873593, KX873986, –, KX873221, KX872920, KX872294^15^. ***Amphibromus neesii*** Steud., AUSTRALIA: Victoria, *Stajsic & Eichler 4243* (MELU-2302051), KX872620, KX873594, KX873987, –, KX873222, KX872921, KX872295^15^. ***Amphibromus pithogastrus*** S.W.L. Jacobs & Lapinpuro, AUSTRALIA: Victoria, *Clarke 2535* (MELU-2028360), KX872621, KX873595, KX873988, –, KX873223, KX872922, KX872296^15^. ***Amphibromus recurvatus*** Swallen, AUSTRALIA: Victoria, *Walker* s.n. (MELU-2100806), KX872622, KX873596, KX873989, –, KX873224, KX872923, KX872297^15^. ***Amphibromus scabrivalvis*** (Trin.) Swallen, CHILE: Region VIII (Bio-Bio), *Soreng & Soreng 7013* (US), KX872623, KX873597, KX873990, KX871946, KX873225, KX872924, –. ***Amphibromus sinuatus*** S.W.L. Jacobs & Lapinpuro, AUSTRALIA: Victoria, *Paget 2257* (MELU-2048802), KX872624, KX873598, KX873991, –, KX873226, KX872925, KX872298^15^.

***Anthoxanthum alpinum*** Á. Löve & D. Löve, SWEDEN: Norrbotten, *Paszko* s.n., sample *GPS 210* (KRAM-559880), KX872625, KX873599, KX873992, KX871947, KX873227, KX872926, KX872299^13^; Norrbotten, *Paszko* s.n., sample *GPS 225* (KRAM-559879), KX872626, KX873600, KX873993, KX871948, KX873228, KX872927, KX872300^13^. ***Anthoxanthum nitens*** (Weber) Y. Schouten & Veldkamp, CANADA: Saskatchewan, *Saarela & Saarela 164* (CAN), KX872627, KX873601, KX873994, KX871950, KX873229, KX872928, –. ***Anthoxanthum odoratum*** L., CANADA: British Columbia, *Saarela 459* (CAN-590334), KX872628, KX873602^6^, KX873995, KX871949, KX873230, KX872929, KX872301^13^; CANADA: British Columbia, *Saarela 500* (CAN-591412), KX872629, –, –, –, –, KX872930, KX872302^13^.

***Apera interrupta*** (L.) P. Beauv., CANADA: British Columbia, *Saarela et al. 647* (CAN-591470), KX872630, KX873603^3^, KX873996, KX871951^8^, KX873231, KX872931, KX872303^15^.

***Arctagrostis latifolia*** (R. Br.) Griseb., CANADA: British Columbia, *Peterson et al. 18459* (CAN-590919), –, KX873604, KX873997, KX871952^8^, KX873232, –, –.

***Arrhenatherum elatius*** (L.) Beauv. ex J. & K. Presl, CANADA: British Columbia, *Saarela et al. 903* (CAN-590503), KX872631, KX873605^1,6^, KX873998, KX871953, KX873233, KX872932, KX872304^10,15^.

***Avellinia michelii*** (Savi) Parl., AUSTRALIA: Western Australia, *Peterson et al. 14246* (US-3422348), KX872632, KX873606^4^, KX873999, KX871954, KX873234, KX872933, KX872305^10,15^.

***Avena fatua*** L., CANADA: British Columbia, *Saarela & Percy 779* (CAN-591524), MF327248, KX873607^6^, KX874000^7^, KX871955, KX873235, –, –. ***Avena sativa*** L., CANADA: British Columbia, *Saarela & Percy 775* (CAN-590451), KX872633, KX873608^6^, KX874001^7^, KX871956, KX873236, KX872934, KX872306^10,15^.

***Beckmannia syzigachne*** (Steud.) Fernald, CANADA: British Columbia, *Saarela et al. 712* (CAN-590403), KX872634, KX873609, KX874002, KX871957^8^, KX873237, –, –.

***Briza minor*** L., PERU: Ancash, *Peterson et al. 21636* (US), KX872635, KX873610^6^, KX874003, KX871958, KX873238, KX872935, KX872307^15^.

***Bromus vulgaris*** (Hook.) Shear, USA: California, *Peterson et al. 19695* (CAN-593921), KM974737, KM974737^1, 3^, KM974737, KM974737, KM974737, KX872936, KX872308^15^.

×***Calammophila baltica*** (Flüggé ex Schrad.) Brand, POLAND: Pomorskie Voivodeship, *Paszko* s.n., sample S-9 (KRAM-558960), KX872895, KX873944, KX874325^7^, KX872260, KX873552, KX873186, KX872588^14^; Pomorskie Voivodeship, *Paszko* s.n., sample J-7 (KRAM-558957), KX872896, KX873945, KX874326, KX872261, KX873553, KX873187, KX872589^14^.

***Calamagrostis × acutiflora*** (Schrad.) DC., USA: Maryland, *Soreng 7411* (US), FJ394557, KX873755, KX874143^7^, KX872101, KX873383, FJ377663, –; SWEDEN: Stockholm, *Paszko* s.n., sample D23.03 GPS 264 (KRAM-558955), KX872737, KX873754, KX874142^7^, KX872100, KX873382, KX873023, KX872420^14^; CANADA: British Columbia, *Peterson et al. 18748* (CAN-590775), KX872738, KX873756, KX874144^7^, KX872102, KX873384, KX873024, KX872421^14^. ***Calamagrostis × gracilescens*** Blytt, POLAND: Mazowieckie Voivodeship, *Paszko* s.n., sample C. gr. XZ (KRAM-558947), KX872739, KX873757, KX874145, KX872103, KX873385, KX873025, KX872422^14^. ***Calamagrostis ampliflora*** Tovar [no name in *Deyeuxia*], PERU: La Libertad, *Peterson et al. 21964* (US), –, KX873845, –, –, –, –, –. ***Calamagrostis angustifolia*** Komarov, RUSSIA: Yakut, *S. Tumanova* s.n. (CAN-408441), KX872637, KX873612, –, KX871960, KX873240, FJ377625, KX872310^14^. ***Calamagrostis*** ***angustifolia*** Komarov subsp. ***angustifolia***, RUSSIA: Chukotka, *Petrovsky & Koroleva* s.n. (CAN-408378), KX872636, KX873611, KX874004, KX871959, KX873239, KX872937, KX872309^14^. ***Calamagrostis anthoxanthoides*** Regel subsp. ***laguroides***, TAJIK SSR: *Gusev & Ikonnikov* s.n. (CAN-327539), KX872640, KX873615, KX874007, KX871963, KX873243, KX872939, KX872313^14^; UZBEK SSR: *Vassilczenko & Vassiljeva 4771* (CAN-327540), KX872638, KX873613, KX874005, KX871961^8^, KX873241, FJ377626, KX872311^14^; TAJIK SSR: *Tolmacheva 7087* (CAN-261634), KX872639, KX873614, KX874006, KX871962, KX873242, KX872938, KX872312^14^. ***Calamagrostis arundinacea*** (L.) Roth, CANADA: British Columbia [cultivated], *Peterson et al. 18747* (CAN-593976), KX872641, KX873616, KX874008, KX871964, KX873249, GQ266675, KX872314^14^; POLAND: Malopolskie Voivodeship, *Paszko* s.n., A-BG3 (KRAM-558950), KX872642, KX873617, KX874009, KX871965, KX873244, KX872940, KX872315^14^; Slaskie Voivodeship, *Paszko* s.n., sample Szyn1 (KRAM-559892), –, –, KX874010, KX871966, KX873245, –, –; CZECH REPUBLIC: *Paszko* s.n., sample Svetnov2a (KRAM-559891), KX872643, KX873618, KX874011, KX871967, KX873246, KX872941, KX872316^14^; SWEDEN: Stockholm, *Paszko* s.n., sample D20.12 4.1 (KRAM-559889), –, KX873619, KX874012, KX871968, KX873247, –, –; CHINA: Bejing, *Soreng et al. 5186* (US-3469013), KX872644, KX873620, KX874013, KX871969, KX873248, KX872942, KX872317^14^; Xizang (Tibet), *Soreng et al. 5599* (US-3491165), KX872766, KX873790, KX874179, KX872136, KX873417, KX873054, KX872453^10,15,16^. ***Calamagrostis bolanderi*** Thurb., USA: California, *Peterson et al. 19694-4* (CAN-593923), KX872645, KX873621^6^, KX874014, KX871970, KX873250, KX872943, KX872318^14^; California, *Peterson et al. 19694-2* (CAN-593923), KX872646, KX873622^6^, KX874015, KX871971, KX873251, KX872944, KX872319^14^. ***Calamagrostis brachytricha*** Steud., RUSSIA: Amur Oblast, *Yakubov* (US-3174890), KX872647, KX873623, KX874016, KX871972, KX873252, KX872945, KX872320^11,14^. ***Calamagrostis cainii*** Hitchc., USA: Tennessee, *Peterson & Saarela 20796* (CAN-602569), KX872648, KX873624, KX874017, KX871973, KX873253, KX872946, KX872321^14^; Tennessee, *Peterson & Saarela 20795* (CAN-602567), KX872649, KX873625, KX874018, KX871974, KX873254, FJ377627, KX872322^14^; California, *Peterson et al. 19822* (CAN-593931), KX872650, KX873626, KX874019, KX871975, KX873255, KX872947, –. ***Calamagrostis canadensis*** subsp. ***langsdorffii*** (Link) Hultén, USA: Washington, *Peterson et al. 18766* (CAN-590819), KX872652, KX873628, KX874021, KX871977, KX873257, KX872949, KX872324^14^; Washington, *Peterson et al. 18761* (CAN-590822), KX872651, KX873627, KX874020, KX871976, KX873256, KX872948, KX872323^14^.Washington, *Peterson et al. 18765* (CAN-590820), KX872655, KX873631, KX874024, KX871980, KX873260, FJ377631, KX872327^14^; CANADA: British Columbia, *Peterson et al. 18455* (CAN-590827), KX872653, KX873629, KX874022, KX871978, KX873258, FJ377629, KX872325^14^; British Columbia, *Peterson et al. 18743* (CAN-590764), KX872654, KX873630, KX874023, KX871979, KX873259, FJ377630, KX872326^14^; Yukon, *Peterson et al. 18498* (CAN-590814), –, KX873632, KX874025, KX871981, KX873261, –, –; Yukon, *Peterson et al. 18615* (CAN-590931), –, KX873633, KX874026, KX871982, KX873262, –, –; British Columbia, *Peterson et al. 18711* (CAN-590946), –, KX873634, KX874027, KX871983, KX873263, –, –; British Columbia, *Peterson et al. 18724* (CAN-590950), –, KX873635, –, KX871984, KX873264, –, –. ***Calamagrostis canescens*** (Weber) Roth, SWEDEN: Västerbotten, *Paszko* s.n., sample D17.07 GPS 247 (KRAM-559875), KX872656, KX873636, KX874028, KX871985, KX873265, KX872950, KX872328^14^; POLAND: Mazowieckie Voivodeship, *Paszko* s.n., sample C-Zbijów 3 (KRAM-558946), KX872657, KX873637, KX874029, KX871986, KX873266, KX872951, KX872329^14^; CZECH REPUBLIC: *Paszko* s.n., sample Svetnov1c, GPS 107 (KRAM-558967), KX872658, KX873638, KX874030, KX871987, KX873267, KX872952, KX872330^14^. ***Calamagrostis carchiensis*** Laegaard, ECUADOR: Carchi, *S. Laegaard 101730* (US-3352675), KX872659, KX873639, KX874031, KX871988, KX873268, KX872953, KX872331^14^. ***Calamagrostis*** cf. ***purpurascens*** R. Br., RUSSIA: Chukotka, *Nechaev et al. 560888* (CAN-560888), KX872730, KX873744, KX874132, KX872090, KX873372, KX873015, –. ***Calamagrostis chalybaea*** Fr., SWEDEN: Jämtland, *Paszko* s.n., sample D17.31 GPS 256 (KRAM-559888), KX872660, KX873640, KX874032, KX871989, KX873269, KX872954, KX872332^14^; Västerbotten, *Paszko* s.n., sample D16.03 GPS 243 (KRAM-559887), KX872661, KX873641, KX874033, KX871990, KX873270, KX872955, KX872333^14^. ***Calamagrostis coahuilensis*** Peterson, Soreng & Valdes-Reyna, MEXICO: Coahuila, *Peterson et al. 8399* (US), FJ394556, –, –, –, –, FJ377633, –. ***Calamagrostis coarctata*** Eaton, USA: Tennessee, *Peterson & Saarela 20818* (CAN-603445), KX872662, KX873642, KX874034, KX871991, KX873271, KX872956, KX872334^14^. ***Calamagrostis deschampsioides*** Trin., CANADA: Yukon, *Bennett et al. 06-381* (US), KX872663, KX873643, KX874035, KX871992, KX873272, KX872957, KX872335^14^; Yukon, *Bennett et al. 06-123* (US), KX872664, KX873644, KX874036, KX871993, KX873273, KX872958, KX872336^14^. ***Calamagrostis distantiflora*** Luczn., RUSSIA: Primorsky Krai (Primorje), *Probatova 5806* (CAN-448522), KX872665, KX873645, KX874037, KX871994, KX873274, KX872959, KX872337^14^. ***Calamagrostis divaricata*** P.M. Peterson & Soreng, MEXICO: Durango, *Peterson & Saarela 21267* (CAN-602202), KX872666, KX873646^4^, KX874038, KX871995, KX873275, KX872960, KX872338^10,15^; Durango, *Peterson et al. 17774* (US-3492608), FJ394559, KX873647^4^, KX874039, KX871996, KX873276, KX872961, KX872339^10,15^. ***Calamagrostis emodensis*** Griseb., CANADA: British Columbia [cultivated], *Peterson et al. 18749* (CAN-959055), FJ377634, KX873648, KX874040, KX871997, KX873277, FJ377634, KX872340^14^; CHINA: Sichuan, *Soreng et al. 5350* (US-3491306), KX872667, KX873649, KX874041, KX871998, KX873278, KX872962, KX872341^14^. ***Calamagrostis epigeios*** (L.) Roth, CANADA: Ontario, *Saarela 1368* (CAN-593981), –, KX873658, KX874049, KX872006, KX873287, –, –; Ontario, *Aiken 86-005* (CAN-516571), KX872668, KX873651, KX874043, KX872000, KX873280, KX872963, KX872342^14^; KYRGYZ REPUBLIC: Naryn, *Soreng et al. 7674* (US-3500194), KX872671, KX873656, KX874047, KX872004, KX873285, KX872967, KX872346^14^; Naryn, *Soreng et al. 7637* (US-3500203), KX872672, KX873657^5^, KX874048, KX872005, KX873286, KX872968, KX872347^14^. POLAND: Pomorskie Voivodeship, *Paszko* s.n., sample J11 (KRAM-559890), KX872669, KX873652, KX874044, KX872001, KX873281, KX872964, KX872343^14;^ POLAND: Pomorskie Voivodeship, *Paszko* s.n., sample S13 (KRAM-558956), –, KX873653, –, –, KX873282, –, –; GERMANY: Saxony, *Paszko* s.n., sample Germany1 (KRAM-558961), KX872670, KX873654, KX874045, KX872002, KX873283, KX872965, KX872344^14^; CHINA: Xilingol, *Soreng et al. 5161* (US-3468996), –, KX873655, KX874046, KX872003, KX873284, KX872966, KX872345^14^; KYRGYZ REPUBLIC: Issyk-Kul: *Soreng et al. 7573b* (US-3500204), –, KX873650^5^, KX874042, KX871999, KX873279, –, –. ***Calamagrostis erectifolia*** Hitchc., MEXICO: Jalisco, *Peterson & Sánchez Alvarado 19105* (US-3496197), FJ394562, KX873659^4^, KX874050, KX872007, KX873288, FJ377635, KX872348^10,15^; Jalisco, *Peterson & Sánchez Alvarado 19106* (US-3496196), FJ394561, KX873660^4^, KX874051, KX872008, KX873289, KX872969, KX872349^10,15^; Jalisco, *Peterson & Sánchez Alvarado 19097* (US-3496203), FJ394560, KX873661^4^, KX874052, KX872009, KX873290, FJ377636, KX872350^10,15^. ***Calamagrostis eriantha*** (Kunth) Steud., MEXICO: Mexico, *Peterson et al. 21344* (CAN-602282), –, KX873662^4^, KX874053, KX872010, KX873291, –, –; Distrito Federal, *Iltis et al. 952* (US-2380074), FJ394564, KX873663^4^, KX874054, KX872011, KX873292, FJ377624, –; Veracruz, *Nee et al. 33190* (US-3338286), FJ394563, KX873664^4^, KX874055, KX872012, KX873293, FJ377653, KX872351^10,15^. ***Calamagrostis foliosa*** Kearney, USA: California, *Peterson et al. 19697* (CAN- 593875), KX872674, KX873667, KX874057, KX872014, KX873295, KX872970, KX872353^14^. ***Calamagrostis guatemalensis*** Hitchc., GUATEMALA: Quetzaltenango, *de Koninck 143* (US-2151654), KX872675, KX873668, KX874058, KX872015, KX873296, KX872971, KX872354^14^. ***Calamagrostis holciformis*** Jaub. & Spach, KYRGYZ REPUBLIC: Naryn, *Soreng et al. 7697* (US-3500184), KX872676, KX873669^1^, KX874059, KX872016, KX873297, KX872972, KX872355^14^. ***Calamagrostis howellii*** Vasey, USA: Washington, *Spellenberg et al. 1189* (CAN-302143), KX872677, KX873670, KX874060, KX872017, KX873298, FJ377638, KX872356^14^. ***Calamagrostis koelerioides*** Vasey, USA: California, *Peterson et al. 19795* (CAN-593872), KX872678, KX873671, KX874061, KX872018, KX873299, KX872974, KX872357^14^; California, *Peterson et al.* 19786 (CAN-593910), KX872679, KX873672, KX874062, KX872019, KX873300, KX872975, KX872358^14^. ***Calamagrostis lapponica*** (Wahlenb.) Hartm., SWEDEN: Norrbotten, *Paszko* s.n., sample D12.05 GPS 211 (KRAM-559882), KX872680, KX873673, KX874063, KX872020, KX873301, KX872976, KX872359^14^, Jämtland, *Paszko* s.n., sample D17.15 GPS 249 (KRAM-559884), –, KX873674, KX874064, KX872021, KX873302, –, –; Norrbotten, *Paszko* s.n., sample D2.09 GPS 134 (KRAM-559878), –, KX873675, KX874065, KX872022, KX873303, –, –; CANADA: Northwest Territories, *Peterson et al. 18585* (CAN-590924), KX872683, KX873680, KX874070, KX872026, KX873308, FJ377639, KX872362^14^; Northwest Territories, *Peterson et al. 18559* (CAN-590801), KX872681, KX873677, KX874067, KX872023, KX873305, KX872977, KX872360^14^; USA: Alaska, *Soreng & Soreng 6163* (US), –, KX873676, KX874066, KX872075, KX873304, –, –; Northwest Territories, *Peterson et al. 18587* (CAN-590926), –, KX873678, KX874068, KX872024, KX873306, –, –; Northwest Territories, *Peterson et al. 18561* (CAN-590802), KX872682, KX873679, KX874069, KX872025, KX873307, KX872978, KX872361^14^. ***Calamagrostis llanganatensis*** Laegaard, ECUADOR: Pastaza, *Laegaard 55455* (US-3352674), KX872685, KX873682, KX874071, KX872027, KX873309, KX872979, KX872363^14^. ***Calamagrostis macrolepis*** Litv., CHINA: Inner Mongolia, *Soreng et al. 5120* (US-3469034), KX872686, KX873683^5^, KX874072, KX872028, KX873310, KX872980, KX872364^14^. ***Calamagrostis montanensis*** (Scribn.) Vasey, CANADA: Alberta, *Peterson et al. 18398* (CAN-591042), –, KX873684, KX874073, KX872029, KX873311, –, –; British Columbia, *Marr 6804* (V), KX872687, –, –, –, –, –, –. ***Calamagrostis muriana*** B.L. Wilson & Sami Gray, USA: California, *Peterson 20930* (US-3526375), KX872688, KX873685, KX874074, KX872030, KX873312, KX872981, KX872365^14^. ***Calamagrostis nutkaensis*** (J. Presl) J. Presl ex Steud., USA: California, *Peterson et al. 19706* (CAN-593866), KX872689, KX873686^3^, KX874075, KX872031, KX873313, KX872982, KX872366^14^; California, *Peterson et al. 19718* (CAN-593864), KX872690, KX873687, KX874076, KX872032, KX873314, KX872983, –. ***Calamagrostis perplexa*** Scribn., USA: New York, *Peterson et al. 20925* (CAN-603496), KX872691, KX873688^5^, KX874077, KX872033, KX873315, FJ377642, KX872367^14^; USA: New York, *Howard 465* (US), KX872692, KX873689^5^, KX874078, KX872034, KX873316, KX872984, KX872368^14^. ***Calamagrostis phragmitoides*** Hartman, CZECH REPUBLIC: Vysocina Region, *Paszko* s.n., sample *Kalište*4 (KRAM-558966), KX872693, KX873690, KX874079, KX872035, KX873317, KX872985, KX872369^14^; SWEDEN: Norrbotten, *Paszko* s.n., sample D16.01 GPS 241 (KRAM-558954), KX872709, KX873711, KX874099, KX872055, KX873338, KX872995, KX872386^14^; Norrbotten, *Paszko* s.n., D13.10 GPS 226 (KRAM-559881), –, KX873712, KX874100, KX872056, KX873339, –, –; Norrbotten, *Paszko* s.n., sample D2.08 GPS 134 (KRAM-559877), –, KX873713, KX874101, KX872057, KX873340, –, –; SWEDEN: Västerbotten, *Paszko* s.n., sample D17.01 GPS 246 (KRAM-559876), KX872710, KX873714, KX874102, KX872058, KX873341, KX872996, KX872387^14^. ***Calamagrostis pickeringii*** A. Gray, USA: New Hampshire, *Peterson & Saarela 20899* (CAN-603466), KX872694, KX873691, KX874080, KX872036, KX873318, KX872986, KX872370^14^; USA: New York, *Peterson & Saarela 20857* (CAN-603450), KX872695, KX873692, KX874081, KX872037, KX873319, FJ377643, KX872371^14^. ***Calamagrostis pisinna*** Swallen, COLOMBIA: Boyacá, *Cleef* (US-2797545), –, KX873693^4^, –, –, KX873320, –, –. ***Calamagrostis porteri*** A. Gray subsp. ***porteri***, USA: Virginia, *Peterson & Saarela 20830* (CAN-603446), KX872696, KX873694, KX874082, KX872038, KX873321, KX872988, KX872372^14^; West Virginia, *Peterson & Saarela 20835* (CAN-603449), KX872697, KX873695, KX874083, KX872039, KX873322, KX872989, KX872373^14^. ***Calamagrostis pringlei*** Scribn. ex Beal, MEXICO: Durango, *Peterson & Saarela* 21257 (CAN-602208), KX872698, KX873696^4^, KX874084, KX872040, KX873323, KX872990, KX872374^10,15^; Chihuahua, *Spellenberg et al. 8878* (NMC-055708), FJ394567, KX873697^4^, KX874085, KX872041, KX873324, FJ377645, KX872375^10,15^. ***Calamagrostis pseudophragmites*** (Haller f.) Koeler, POLAND: Malopolskie Voivodeship, *Paszko* s.n., sample Oblaz-pseudo2 (KRAM-559893), KX872699, KX873698, KX874086, KX872042, KX873325, FJ377646, KX872376^14^; CHINA: Hebei, *Soreng et al. 5107* (US-3469048), KX872700, KX873699, KX874087, KX872043, KX873326, KX872991, KX872377^14^; Qinghai, *Soreng et al. 5455* (US-3491820), KX872701, KX873700, KX874088, KX872044, KX873327, KX872992, KX872378^14^. ***Calamagrostis pseudophragmites*** subsp. ***tartarica*** (Hook. f.) Tzvelev, KYRGYZ REPUBLIC: Chu, *Soreng et al. 7534* (US-3500199), KX872702, KX873701, KX874089, KX872045, KX873328, KX872993, KX872379^14^; Chu, *Soreng et al. 7556* (US-3500200), KX872703, KX873702, KX874090, KX872046, KX873329, KX872994, KX872380^14^. ***Calamagrostis purpurascens*** R. Br., CANADA: Northwest Territories, *Peterson et al. 18569* (CAN-590803), KX872704, KX873703, KX874091, KX872047, KX873330, FJ377648, KX872381^14^; Alberta, *Peterson et al. 18415* (CAN-590798), KX872706, KX873705, KX874093, KX872049, KX873332, FJ377650, KX872383^14^; Yukon, *Peterson et al. 18609* (CAN-590796), –, KX873706, KX874094, KX872050, KX873333, –, –; Yukon, *Peterson et al. 18492* (CAN-590784), KX872707, KX873707, KX874095, KX872051, KX873334, FJ377659, KX872384^14^; Yukon, *Peterson et al. 18474* (CAN-590829), KX872708, KX873708, KX874096, KX872052, KX873335, FJ377647, KX872385^14^; Yukon, *Peterson et al. 18545* (CAN-590804), –, KX873709, KX874097, KX872053, KX873336, –, –; Yukon, *Peterson et al. 18652* (CAN-590797), –, KX873710, KX874098, KX872054, KX873337, –, –; USA: Alaska, *Peterson et al. 18500* (CAN-590813), KX872705, KX873704, KX874092, KX872048, KX873331, FJ377649, KX872382^14^. ***Calamagrostis rivalis*** H. Scholz, Germany: Saxony, *Paszko* s.n., sample Wald5 GPS 67 (KRAM-558963), KX872711, KX873715, KX874103, KX872059, KX873342, KX872997, KX872388^14^; GERMANY: Saxony, *Paszko* s.n., sample Kloster4 (KRAM-558964),, KX873716, KX874104, KX872060, KX873343, –, –; Saxony, *Paszko* s.n.,. sample Welch 3 (KRAM-558962), KX872712, KX873717, KX874105, KX872061, KX873344, KX872998, KX872389^14^. ***Calamagrostis rubescens*** Buckley, USA: Oregon, *Peterson et al. 18769* (CAN-590816), KX872714, KX873720, KX874108, KX872064, KX873347, KX872999, KX872391^14^; Washington, *Peterson & Saarela 18767* (CAN-590818), –, KX873719, KX874107, KX872063, KX873346, –, –; Washington, *Peterson & Saarela 18768* (CAN-590817), –, KX873721, KX874109, KX872065, KX873348, –, –; CANADA: British Columbia, *Peterson et al. 18741* (CAN-590765), KX872713, KX873718, KX874106, KX872062, KX873345, FJ377652, KX872390^14^. ***Calamagrostis scopulorum*** M.E. Jones, USA: Utah, *Franklin 5569* (CAN-531332), KX872715, KX873722, KX874110, KX872066, KX873349, FJ377655, KX872392^14^; Colorado, *Soreng & Soreng 7423* (US), FJ394571, KX873723, KX874111, KX872067, KX873350, FJ377654, KX872393^14^. ***Calamagrostis sesquiflora*** (Trin.) Kawano, CANADA: British Columbia, *Cheney* s.n. (no voucher), KX872716, KX873724, KX874112, KX872068, KX873351, KX873000, KX872394^14^; British Columbia, *Saarela & Percy 1223* (CAN-590636), KX872717, KX873725, KX874113, KX872069, KX873352, KX873001, KX872395^14^; British Columbia, *Saarela & Percy 1229* (CAN-590639), KX872718, KX873726, KX874114, KX872070, KX873353, KX873002, KX872396^14^. ***Calamagrostis stricta*** (Timm) Koeler, SWEDEN: Norrbotten, *Paszko* s.n., sample D12.01 GPS 206 (KRAM-559883), KX872723, KX873735, KX874123, KX872081, KX873363, FJ377658, KX872405^14^; NORWAY: Svalbard, *Paszko* s.n., sample Svalbard 2 (KRAM-559886), KX872724, KX873736, KX874124, KX872082, KX873364, KX873009, KX872406^14^; ICELAND: *Paszko* s.n., sample Iceland 2.5 (KRAM-558948), KX872725, KX873737, KX874125, KX872083, KX873365, KX873010, KX872407^14^; ARGENTINA: Santa Cruz, *Peterson et al. 17076* (US), –, KX873738, KX874126, KX872084, KX873366, –, –; Santa Cruz, *Peterson et al. 17119* (US-3452760), KX872726, KX873740, KX874128, KX872086, KX873368, KX873012, KX872409^14^; Santa Cruz, *Peterson et al. 17076* (US-3450440), KX872727, KX873741, KX874129, KX872087, KX873369, KX873013, KX872410^14^. ***Calamagrostis stricta*** subsp. ***stricta***, KYRGYZSTAN: Chu, *Soreng et al. 7722* (US0-3500195), KX872728, KX873742, KX874130, KX872088, KX873370, KX873014, KX872411^14^; CANADA: Yukon, *Peterson et al. 18604* (CAN-590928), KX872729, KX873743^5^, KX874131, KX872089, KX873371, FJ377659, KX872412^14^; Yukon, *Peterson et al. 18616* (CAN-590932), KX872684, KX873739, KX874127, KX872085, KX873367, FJ377656, KX872408^14^. ***Calamagrostis stricta*** subsp. ***groenlandica*** (Schrank) Á. Löve, CANADA: Yukon, *Bennett et al. 06-132* (US), KX872719, KX873728, KX874115, KX872071, KX873355, KX873004, KX872398^14^; Yukon, *Bennett et al. 06-208* (US), –, KX873729, KX874116, KX872072, KX873356, –, –; Yukon, *Bennett et al. 06-246* (US), KX872720, KX873730, KX874117, KX872073, KX873357, KX873005, KX872399^14^; Yukon, *Bennett et al. 06-177* (US), –, –, –, KX872074, –, –, –; USA: Alaska, *Soreng 6204* (US), KX872721, KX873731, KX874118, KX872076, KX873358, FJ377637, KX872400^14^. ***Calamagrostis stricta*** subsp. ***inexpansa*** (A. Gray) C.W. Greene, USA: Alaska, *Talbot ADA052-20* (US-3543902), –, KX873732, KX874119, KX872077, KX873359, KX873007, KX872401^14^; Alaska, *Talbot AIK010-06* (US-3543901), FJ394564, KX873733, KX874120, KX872078, KX873360, –, KX872402^14^; Alaska, *Talbot ADA030-25* (US-3543900), –, KX873734, KX874121, KX872079, KX873361, KX873008, KX872403^14^; CANADA: Yukon, *Peterson et al. 18618* (CAN-590933), KX872722, KX873681, KX874122, KX872080, KX873362, FJ377640, KX872404^14^. ***Calamagrostis tolucensis*** (Kunth) Trin., MEXICO: Morelos, *Peterson et al. 21395* (CAN-602283), –, KX873745^4^, KX874133, KX872091, KX873373, –, –; Mexico, *Peterson et al. 21343* (CAN), –, KX873746^4^, KX874134, KX872092, KX873374, –, –; Mexico, *Peterson & Herrera-Arrieta 16152* (US-3481473), FJ394573, –,–, –, –, FJ377660, –; Oaxaca, *Gereau et al. 19697* (US-3251914), FJ394574, –,–, –, –, FJ377661, –. ***Calamagrostis valida*** Sohns, MEXICO: Jalisco, *Villa C. et al. 921* (ANSM-54393), FJ394575, –,–, –, –, KX873016, –. ***Calamagrostis varia*** (Schrad.) Host, POLAND: Malopolskie Voivodeship, *Paszko* s.n., sample va-Bocz (KRAM-558949), KX872731, KX873747, KX874135, KX872093, KX873375, KX873017, KX872413^14^; SWEDEN: Gotland, *Paszko* s.n., sample D22.11 GPS 263 (KRAM-559885), KX872732, KX873748, KX874136, KX872094, KX873376, KX873018, KX872414^14^; AUSTRIA: Salzburg Land, *Paszko* s.n., sample Schmittenhöhe var 4 GPS 288 (KRAM-558953), KX872733, KX873749, KX874137, KX872095, KX873377, KX873019, KX872415^14^. ***Calamagrostis villosa*** (Chaix) J.F. Gmelin, AUSTRIA: Salzburg Land, *Paszko* s.n., sample Hundstein 1 villosa GPS 278 (KRAM-558952), KX872734, KX873750, KX874138, KX872096, KX873378, KX873020, KX872416^14^; POLAND: Malopolskie Voivodeship, *Paszko* s.n., sample vi3-BG (KRAM-558951), KX872735, KX873751, KX874139, KX872097, KX873379, KX873021, KX872417^14^; CZECH REPUBLIC: *Paszko* s.n., sample Cikháj1, GPS 109 (KRAM-558965), KX872736, KX873752, KX874140, KX872098, KX873380, KX873022, KX872418^14^. ***Calamagrostis vulcanica*** Swallen, GUATEMALA: San Marcos, *Gallardo et al. 9092* (US), FJ394576, KX873753^4^, KX874141, KX872099, KX873381, FJ377662, KX872419^10,15^.

***Chascolytrum brizoides*** (Lam.) Desv., CHILE: Region VIII (Bio-Bio), *Soreng & Soreng 7014* (US), KX872740, KX873758, KX874146, KX872104, KX873386, KX873026, –. ***Chascolytrum monandrum*** (Hack.) Matthei, PERU: Ancash, *Peterson et al. 21704* (US), KX872741, KX873759, KX874147, KX872105, KX873387, KX873027, KX872423^14^; Cajamarca, *Peterson & Soreng 21861* (US), KX872742, KX873760, KX874148, KX872106, KX873388, KX873028, KX872424^14^; CHILE: Region VIII (Bio-Bio), *Soreng & Soreng 7020* (US), FJ394578, KX873761, KX874149, KX872107, KX873389, FJ377665, KX872425^14^. ***Chascolytrum subaristatum*** (Lam.) Desv., CHILE: Region VIII (Bio-Bio), *Soreng & Soreng 7005* (US), FJ394577, KX873762, KX874150, KX872108, KX873390, FJ377664, KX872426^15^.

***Cinna latifolia*** (Trevir. ex Göpp.) Griseb., CANADA: British Columbia, *Saarela & Percy 1073* (CAN-590607), –, KX873763, KX874151, KX872109^8^, KX873391, –, KX872427^15^.

***Deschampsia cespitosa*** (L.) P. Beauv., USA: California, *Peterson et al. 19754* (CAN-593939), –, KX873764, KX874152, KX872110, KX873392, KX873029, KX872428^15^; CANADA: Alberta, *Peterson & Saarela 18410* (CAN-590916), KX872743, KX873765, KX874153, KX872111, –, KX873030, KX872429^15^; Saskatchewan, *Peterson et al. 18367* (CAN-590855), KX872744, KX873766, KX874154, KX872112, –, KX873031, KX872430^15^; CHILE: Region II (Antofagasta), *Peterson & Soreng 15587* (US-3446825), FJ394554, KX873767, KX874155, KX872113, KX873393, KX873032, KX872431^15^. ***Deschampsia elongata*** (Hook.) Munro, USA: California, *Peterson et al. 19760* (CAN-593944), –, KX873768, KX874156, KX872114, KX873394, –, –; California, *Peterson et al. 19801* (CAN-593917), KX872745, KX873769, KX874157, KX872115, KX873395, KX873033, KX872432^15^; California, *Peterson et al. 19657* (CAN-593882), KX872746, KX873770, KX874158, KX872116, KX873396, KX873034, KX872433^15^.

***Deyeuxia aurea*** Munro ex Wedd., ECUADOR: Pichincha, *Peterson et al. 9081* (US-3237443), KX872747, KX873771, KX874159, KX872117, KX873397, KX873035, KX872434^15^. ***Deyeuxia breviaristata*** Wedd., CHILE: Region I (Tarapacá), *Peterson & Soreng 15720* (US-3444533), KX872748, KX873772^4^, KX874160, KX872118, KX873398, KX873037, KX872436^10,15^; BOLIVIA: Potosi, *Peterson et al. 12825* (US-3277090), KX872749, KX873773^4^, KX874161, KX872119, KX873399, KX873038, KX872437^10,15^; Potosi, *Peterson et al. 12842* (US-3275987), KX872750, KX873774^4^, KX874162, KX872120, KX873400, KX873036, KX872435^10,15^. ***Deyeuxia brevifolia*** J. Presl, CHILE: Region III (Atacama), *Peterson et al. 15459* (US-3445692), –, –, KX874163, KX872121, KX873401, MF348279, –; Region III (Atacama), *Peterson et al. 15459* (US-3445692), KX872751, KX873775^4^, KX874164, KX872122, KX873402, –, KX872438^10,15^; BOLIVIA: Poopo, *Peterson et al. 12708* (US-3275592), KX872752, KX873776^4^, KX874165, KX872123, KX873403, KX873039, KX872439^10,15^. ***Deyeuxia cabrerae*** (Parodi) Parodi, CHILE: Region II (Antofagasta), *Peterson et al. 15574* (US-3445690), KX872753, KX873777^4^, KX874166, KX872124, KX873404, KX873040, KX872440^10,15^. ***Deyeuxia*** cf. ***macrophylla*** Pilg., PERU: Cajamarca, *Peterson & Soreng 21867* (US), –, KX873835, –, –, –, –, –. ***Deyeuxia chrysantha*** J. Presl, PERU: Ancash, *Peterson et al. 21763* (US), –, KX873844, –, –, –, –, –. ***Deyeuxia chrysantha*** var. ***chrysantha***, PERU: Tarata, *Peterson et al. 14822* (US-3449420), KX872754, KX873778, KX874167, KX872125, KX873405, KX873041, KX872441^15^. ***Deyeuxia chrysantha*** var. ***phalaroides*** (Wedd.) Villav., BOLIVIA: Potosi, *Peterson et al. 13064* (US-3277157), KX872755, KX873779, KX874168, KX872126, KX873406, KX873042, KX872442^15^. ***Deyeuxia chrysophylla*** Phil., BOLIVIA: Potosi, *Peterson et al. 13027* (US-3276621), KX872756, KX873780^4^, KX874169, KX872127, KX873407, KX873043, KX872443^10,15^. ***Deyeuxia coarctata*** Kunth, PERU: Junín, *Peterson & Tovar 14064* (US-3421420), KX872673, KX873666^4^, KX874056, KX872013, KX873294, KX873044, KX872352^10,15^. ***Deyeuxia crispa*** Rúgolo & Villav., BOLIVIA: Potosi, *Peterson et al. 12875* (US-3281767), KX872757, KX873781^4^, KX874170, KX872128, KX873408, KX873045, KX872444^10,15^; Sud Chichas, *Peterson et al. 12942* (US-3276499), KX872758, KX873782^4^, KX874171, KX872129, KX873409, KX873046, KX872445^10,15^. ***Deyeuxia curvula*** Wedd., BOLIVIA: La Paz, *Peterson et al. 13194* (US-3264866), KX872759, KX873783^4^, KX874172, KX872130, KX873410, KX873047, KX872446^10,15^; PERU: Tacna, *Peterson et al. 14733* (US-3449441), KX872760, KX873784^4^, KX874173, KX872131, KX873411, –, KX872447^10,15^. ***Deyeuxia densiflora*** Presl, PERU: Ancash, *Peterson & Soreng 17943* (US-3491347), KX872761, KX873785^4^, KX874174, –, KX873412, KX873048, KX872448^10,15^; Lima or Junín, *Cook & Cook 22* (US-3098626), KX872762, KX873786^4^, KX874175, KX872132, KX873413, KX873049, KX872449^10,15^. ***Deyeuxia deserticola*** var. ***breviaristata*** Rúgolo & Villav., CHILE: Region I (Tarapacá), *Peterson & Soreng 15726* (US-3444539), KX872763, KX873787^4^, KX874176, KX872133, KX873414, KX873050, KX872450^10,15^; BOLIVIA: Potosi, *Peterson et al. 13007A* (US-3479005), KX872764, KX873788^4^, KX874177, KX872134, KX873415, KX873051, KX872451^10,15^. ***Deyeuxia diffusa*** Keng, CHINA: Yunnan, *Soreng et al. 5272* (US-3475135), KX872771, KX873795^6^, KX874183, KX872140, KX873422, KX873059, KX872458^10,15^; Yunnan, *Soreng et al. 5233b* (US), KX872772, KX873796^6^, KX874184, KX872141, KX873423, KX873060, KX872459^10,15^. ***Deyeuxia effusa*** Kunth, COLOMBIA: Cundinamarca, *Gomez 21* (US-3534977), KX872765, KX873789, KX874178, KX872135, KX873416, KX873052, KX872452^14^; VENEZUELA: Paramo de Guirigay, *Stergios 19875* (US-3452651), KX872796, KX873822, –, –, MF346087, KX873053, KX872483^14^. ***Deyeuxia eminens*** var. ***inclusa*** Rúgolo, ARGENTINA: Agua Negra, *Peterson et al. 19307* (US-3532258), KX872767, KX873791, KX874180, KX872137, KX873418, KX873055, KX872454^15^. ***Deyeuxia fiebrigii*** (Pilg.) Rúgolo, BOLIVIA: Potosi, *Peterson et al. 12966* (US-3277020), KX872768, KX873792^4^, KX874181, KX872138, KX873419, KX873056, KX872455^10,15^. ***Deyeuxia filifolia*** Wedd., BOLIVIA: La Paz, *Peterson et al. 13196* (US-3264875), KX872769, KX873793^4^, KX874182, KX872139, KX873420, KX873057, KX872456^10,15^; Potosi, *Peterson et al. 13099* (US-3277192), KX872770, KX873794^4^, –, –, KX873421, KX873058, KX872457^10,15^. ***Deyeuxia glacialis*** Wedd., BOLIVIA: La Paz, *Peterson et al. 12608* (US-3279308), KX872775, KX873799^4^, KX874187, –, KX873426, KX873063, KX872462^10,15^. ***Deyeuxia hackelii*** (Lillo) Parodi, BOLIVIA: Potosi, *Peterson et al. 13032* (US-3276633), KX872776, KX873800, KX874188, –, KX873427, KX873064, KX872463^15^. ***Deyeuxia heterophylla*** (Wedd.) Pilg., PERU: Junín, *Peterson & Tovar 14129* (US-3421325), KX872777, KX873801^4^, KX874189, KX872144, KX873428, KX873065, KX872464^10,15^; Ancash, *Peterson et al. 21674* (US), –, KX873839^4^, KX874221, –, –, –, –; Ancash, *Peterson et al. 21483* (US), –, KX873841^4^, KX874223, –, –, –, –; Ancash, *Peterson et al. 21561* (US), KX872778, KX873802^4^, KX874190, KX872145, KX873429, KX873066, KX872465^10,15^; CHILE: Region I (Tarapacá), *Peterson & Soreng 15735* (US-3444547), KX872779, KX873803^4^, KX874191, KX872146, KX873430, –, –. ***Deyeuxia intermedia*** J. Presl, PERU: Ancash, *Peterson et al. 21599* (US), –, KX873836^4^, KX874218, –, –, –, –; ECUADOR: Cotopaxi, *Sklenar & Kosteckova 86-19* (US-3338690), KX872780, KX873804^4^, KX874192, KX872147, KX873431, KX873067, KX872466^10,15^; COSTA RICA: Limon-Puntarenas border, *Davidse & Herrera 29411* (US-3041607), –, KX873805^4^, KX874193, –, –, –, –. ***Deyeuxia jamesonii*** (Steud.) Munro ex Wedd., ECUADOR: Pichincha, *Holm-Nielsen 24232* (US-3450184), KX872781, KX873806^4^, KX874194, –, KX873432, –, KX872467^10,15^; BOLIVIA: La Paz, *Peterson et al. 13195* (US-3264873), KX872782, KX873807^4^, KX874195, KX872148, KX873433, KX873068, KX872468^10,15^. ***Deyeuxia lagurus*** Wedd., PERU: Moquegua, *Peterson & Refulio-Rodriguez 18311* (US-3491413), KX872783, KX873808^4^, KX874196, KX872149, KX873434, KX873069, KX872469^10,15^; BOLIVIA: Potosi, *Peterson et al. 13031* (US-3264873), KX872784, KX873809^4^, KX874197, KX872150, KX873435, KX873070, KX872470^10,15^. ***Deyeuxia ligulata*** Kunth, ECUADOR: Carchi, *Sklenar & Kosteckova 46-1* (US-3338693), KX872785, KX873810, –, –, KX873436, KX873071, KX872471^15^. ***Deyeuxia macrophylla*** Pilg., PERU: Ancash, *Peterson et al. 21642* (US), –, KX873840^4^, KX874222, –, –, –, –. ***Deyeuxia malamalensis*** (Hack.) Parodi, BOLIVIA: La Paz, *Solomon 15046* (US-3198776), KX872786, KX873811^4^, –, –, KX873437, KX873072, KX872472^10,15^. ***Deyeuxia mandoniana*** Wedd., BOLIVIA: La Paz, *Renvoize 4784* (US-3480776), KX872787, KX873812^4^, KX874198, –, KX873438, KX873073, KX872473^10,15^; La Paz, *Peterson et al. 13197* (US-3264877), KX872788, KX873813^4^, KX874199, KX872151, KX873439, KX873074, KX872474^10,15^. ***Deyeuxia mazzettii*** Veldkamp, CHINA: Yunnan, *Soreng et al. 5314* (US-3491314), KX872789, KX873814^6^, KX874200, KX872152, KX873440, KX873075, KX872475^10,15^. ***Deyeuxia nitidula*** (Pilg.) Rúgolo, PERU: Huancavelica, *Peterson & Tovar 14204* (US-3421366), –, KX873815^4^, KX874201, –, –, –, KX872476^10,15^; BOLIVIA: Murillo, *Solomon 13638* (US-3071954), KX872790, KX873816^4^, KX874202, KX872153, KX873441, KX873076, KX872477^10,15^; Huancavelica, *Peterson & Refulio 18120* (US-3491352), KX872791, KX873817^4^, KX874203, KX872154, KX873442, KX873077, KX872478^10,15^. ***Deyeuxia nivicola*** Hook. f., CHINA: Xizang (Tibet), *Soreng et al. 5648* (US-3491150), KX872792, KX873818^6^, KX874204, KX872155, KX873443, KX873078, KX872479^10,15^. ***Deyeuxia nyingchiensis*** P.C. Kuo & S.L. Lu, CHINA: Xizang (Tibet), *Soreng et al. 5578* (US-3491162), KX872793, KX873819, KX874205, KX872156, KX873444, KX873079, KX872480^10,15^. ***Deyeuxia ovata*** J. Presl, PERU: Ancash, *Peterson et al. 21675* (US), KX872795, KX873821, KX874207, KX872158, KX873446, KX873081, KX872482^15^. ***Deyeuxia ovata*** var. ***ovata***, PERU: El Collao, *Peterson et al. 14579* (US-3428514), KX872794, KX873820, KX874206, KX872157, KX873445, KX873080, KX872481^15^. ***Deyeuxia planifolia*** Kunth, ECUADOR: Carchi, *Peterson et al. 9129* (US-3237473), KX872797, KX873823^4^, –, –, KX873447, KX873082, KX872484^10,15^. ***Deyeuxia podophora*** (Pilg.) Sodiro, ECUADOR: Pichincha, *Laegaard et al. 1546* (US-3450197), KX872798, KX873824, KX874208, KX872159, KX873448, KX873083, KX872485^15^. ***Deyeuxia nana*** Rúgolo [originally det. as *D. preslii* (Kunth) Hitchc., misapplied] PERU: Junín, *Peterson & Refulio Rodriguez 18047* (US-3491408), FJ394565, KX873825^4^, KX874209, KX872160, KX873449, KX873084, KX872486^10,15^; Pasco, *Peterson & Tovar 14100* (US-3421437), KX872799, KX873826^4^, KX874210, KX872161, KX873450, FJ377641, KX872487^10,15^. ***Deyeuxia pulchella*** (Griseb.) Hook. f., CHINA: Xizang (Tibet), *Soreng et al. 5586* (US), KX872800, KX873827, KX874211, KX872162, KX873452, KX873085, KX872488^10,15^; Yunnan, *Soreng et al. 5278* (US-3475142), KX872773, KX873797^4^, KX874185, KX872142, KX873424, KX873061, KX872460^10,15^. ***Deyeuxia recta*** Kunth, PERU: Cajamarca, *Peterson & Soreng 21865* (US), –, KX873837, KX874219, –, –, –, –; COLOMBIA: Santander, *Gomez 32* (US-3534976), –, KX873828^4^, KX874212, KX872163, KX873451, KX873086, KX872489^10,15^. ***Deyeuxia rigescens*** (J. Presl) Turpe, PERU: Ancash, *Peterson et al. 21744* (US), KX872801, KX873829^4^, –, –, KX873453, KX873087, KX872490^10,15^; Ancash, *Peterson et al. 21516* (US), KX872802, KX873830^4^, –, –, KX873454, KX873088, KX872491^10,15^. ***Deyeuxia rigida*** Kunth, PERU: Ancash, *Peterson et al. 21502* (US), –, KX873842, KX874224, –, –, –, –; Ancash, *Peterson et al. 21528* (US), –, –, KX874225, –, –, –, –; Ancash, *Peterson & Soreng 21843* (US), –, –, KX874226, –, –, –, –; ECUADOR: Carchi, *Peterson et al. 9143* (US-3237464), KX872803, KX873831^4^, KX874213, KX872164, KX873455, KX873089, KX872492^10,15^; BOLIVIA: La Paz, *Peterson et al. 12678* (US-3275565), KX872804, KX873832^4^, KX874214, KX872165, KX873456, KX873090, KX872493^10,15^. ***Deyeuxia rosea*** Bor, CHINA: Xizang (Tibet), *Soreng et al. 5542* (US), –, –, KX874215, KX872166, KX873457, –, –. ***Deyeuxia rupestris*** (Trin.) Rúgolo, BRAZIL: Rio Grande do Sul, *Scur 920* (US-3432533), KX872805, KX873833^4^, KX874216, KX872167, KX873458, KX873091, KX872494^10,15^; BOLIVIA: La Paz, *Renvoize & Cope 4176* (US-3104208), KX872806, KX873834^4^, KX874217, KX872168, KX873459, KX873092, KX872495^10,15^. ***Deyeuxia scabrescens*** (Griseb.) Munro ex Duthie, CHINA: Xizang (Tibet), *Soreng et al. 5613* (US-3491158), –, KX873847, KX874227, KX872169, KX873460, KX873093, KX872496^10,15^; Qinghai, *Soreng et al. 5424* (US-3482584), KX872807, KX873848, KX874228, KX872170, KX873461, KX873094, KX872497^10,15^. ***Deyeuxia setiflora*** Wedd., CHILE: Region 1, *Peterson & Soreng 15727* (US-3444540), –, KX873849^4^, KX874229, KX872171, KX873462, KX873095, KX872498^10,15^. ***Deyeuxia sichuanensis*** (J.L. Yang) S.M. Phillips & W.L. Chen, CHINA: Xizang (Tibet), *Soreng et al. 5659* (US-3491160), KX872808, KX873850, KX874230, KX872172, KX873463, KX873096, KX872499^10,15^. ***Deyeuxia spicigera*** J. Presl, PERU: Arequipa, *Peterson & Refulio Rodriguez 18275* (US-3491385), KX872809, KX873727^4^, –, –, KX873354, KX873003, KX872397^10,15^. ***Deyeuxia spicigera*** var. ***spicigera***, PERU: Ancash, *Peterson et al. 21588* (US), –, KX873838^4^, KX874220, –, –, –, –. ***Deyeuxia tarmensis*** (Pilg.) Sodiro, PERU: Ancash, *Peterson & Refulio Rodriguez 13929* (US-3423010), KX872810, KX873851^4^, KX874231, KX872173, KX873464, KX873097, KX872500^10,15^. ***Deyeuxia trichodonta*** Wedd., BOLIVIA: Potosi, *Peterson et al. 12960* (US-3277014), KX872811, KX873852^4^, KX874232, KX872174, KX873465, KX873098, KX872501^10,15^. ***Deyeuxia tripilifera*** (Hook. f.) Keng, CHINA: Gansu, *Soreng et al. 5385* (US-3482569), KX872774, KX873798^6^, KX874186, KX872143, KX873425, KX873062, KX872461^10,15^. ***Deyeuxia velutina*** Nees & Meyen, CHILE: Region III (Atacama), *Peterson et al. 15489* (US), KX872812, KX873853^4^, KX874233, KX872175, KX873466, KX873099, KX872502^10,15^; CHILE: Region III (Atacama), *Peterson et al. 15484* (US-3445713), KX872813, KX873854^4^, KX874234, KX872176, KX873467, KX873100, KX872503^10,15^. ***Deyeuxia vicunarum*** Wedd., PERU: Ancash, *Peterson et al. 21496* (US), –, KX873843^4^, –, –, –, –, –; Ancash, *Peterson et al. 21580* (US), KX872814, KX873855^4^, KX874235, KX872177, KX873468, KX873101, KX872504^10,15;^ Ancash, *Peterson et al. 21496* (US), –, KX873846^4^, –, –, –, –, –; Ancash, *Peterson & Refulio Rodriguez 13824* (US-3423064), KX872815, KX873856^4^, KX874236, KX872178, KX873469, KX873102, KX872505^10,15^. ***Deyeuxia violacea*** Wedd., BOLIVIA: Potosi, *Peterson et al. 12965* (US-3277019), KX872817, KX873858^4^, KX874238, KX872180, KX873471, KX873104, KX872507^10,15^. ***Deyeuxia violacea*** Wedd. var. ***puberula***, BOLIVIA: La Paz, *Peterson et al. 13202* (US-3264869), KX872816, KX873857^4^, KX874237, KX872179, KX873470, KX873103, KX872506^10,15^. ***Deyeuxia viridiflavescens*** (Poir.) Kunth, PERU: Cajamarca, *Peterson & Refulio Rodriguez 15032* (US-3420282), KX872818, KX873860^4^, KX874240, KX872182, KX873473, KX873106, KX872509^10,15^; BRAZIL: Caxias do Sul, *Scur 1150* (US-3486684), KX872819, KX873861^4^, KX874241, KX872183, KX873474, KX873107, KX872510^10,15^. ***Deyeuxia viridiflavescens*** var. ***montevidensis*** (Nees) Cabrera & Rúgolo, BRAZIL: Caxias do Sul, *Barreto 17* (US-3455238), –, KX873859^4^, KX874239, KX872181, KX873472, KX873105, KX872508^10,15^. ***Deyeuxia viridis*** Phil., ARGENTINA: Neuquén, *Peterson et al. 17371* (US-3451012), KX872820, KX873862^4^, KX874242, –, KX873475, KX873108, KX872511^10,15^.

***Echinopogon caespitosus*** C.E. Hubb. var. ***caespitosus***, AUSTRALIA: New South Wales, *Soreng et al. 5900* (US), KX872821, KX873863^1^, KX874243, KX872184, KX873476, KX873109, KX872512^14^.

***Gaudinia fragilis*** (L.) P. Beauv., URUGUAY: Cerro Largo, *Seijo et al. 2557* (US-3517052), KX872822, KX873864^1,6^, KX874244, KX872185, KX873477, KX873110, KX872513^10,12,15^; AUSTRALIA: Victoria, *Stajsic & Eichler 4229* (MELU-2302034), KX872823, KX873865^1,6^, KX874245, KX872186, KX873478, KX873111, KX872514^10,12,15^; Victoria, *Paget 1558* (MELU-2042425), KX872824, KX873866^1,6^, KX874246, KX872187, KX873479, KX873112, KX872515^10,12,15^.

***Graphephorum wolfii*** Vasey, USA: Colorado, *Porsild & Weber 22911* (CAN-266457), KX872825, KX873942^4^, KX874323, KX872258, KX873480, KX873113, KX872586^10,15^.

***Helictotrichon tianschanicum*** (Roshev.) Henrard, KYRGYZ REPUBLIC: Issyk-Kul, *Soreng et al. 7580* (US-3500051), KX872826, KX873867^3^, KX874247, KX872188, KX873481, KX873114, –.

***Holcus lanatus*** L., CANADA: British Columbia, *Peterson et al. 18732* (CAN-591082), KX872827, KX873868, KX874248, KX872189, KX873482, KX873115, –.

***Koeleria capensis*** (Steud.) Nees, Transvaal, *Smook 2559* (US-3184956), KX873869^1,6^, KX874249, KX872190, KX873483, KX873116, KX872516^10,15^; SOUTH AFRICA: Eastern Cape, *Smook 10302* (US-3428003), KX872828, KX873870^1,6^, KX874250, –, KX873484, KX873117, KX872517^10,15^. ***Koeleria lobata*** (Bieb.) Roem. & Schult., GREECE: Peleponnissos, *Soreng 3813b* (US-3483169), KX872829, –, KX874251, KX872191, KX873485, KX873118, KX872518^10,15^. ***Koeleria macrantha*** (Ledeb.) Schult., USA: California, *Peterson et al. 19783* (CAN-593900), KX872830, KX873871^1,6^, KX874252^7^, KX872192, KX873486, KX873119, –. ***Koeleria permollis*** Nees ex Steud., PERU: Ancash, *Peterson et al. 21705* (US), KX872831, KX873872^1,6^, KX874253^7^, KX872193, –, KX873120, KX872519^10,15^. ***Koeleria splendens*** C. Presl, GREECE: Thessaly, *Soreng et al. 7491* (US-3555907), KX872832, KX873873^1,6^, KX874254, KX872194, KX873487, KX873121, KX872520^10,15^. ***Koeleria vallesiana*** Asch. & Graebn., France: Aude, *Soreng & Maumont 4005b* (US-3483168), KX872833, KX873874^1,6^, KX874255, KX872195, KX873488, KX873122, –.

***Lagurus ovatus*** L., MOROCCO: Oujda, *Aurich & Forther* (US-3343370), KX872834, KX873876^3^, KX874257, –, KX873490, KX873123, –; CHILE: Region VI (O’Higgins), *Lammers et al. 7904* (US-3213955), –, –, –, –, –, MF348274, –; AUSTRALIA: Western Australia, *Soreng & Rosenberg 14484* (US-3422325), KX872835, KX873875^3^, KX874256, –, KX873489, KX873124, –.

***Leptophyllochloa micrathera*** (E. Desv.) C.E. Calderon, ARGENTINA: Neuquén, *Peterson et al. 17403* (US-3452785), KX872836, KX873877^4^, KX874258, KX872196, KX873491, KX873125, KX872521^10,15^.

***Peyritschia deyeuxiodes*** (Kunth) Finot, MEXICO: Puebla, *Peterson et al. 21403* (CAN-602533), KX872837, KX873879^4^, KX874260, KX872198, KX873493, KX873126, KX872523^10,15^; Nuevo Leon, *Peterson & Saarela 21110* (CAN-602439), KX872838, KX873880^4^, KX874261, KX872199, KX873494, KX873127, KX872524^10,15^; Oaxaca, *Peterson & Campos-Villanueva 9809* (US), FJ394580, KX873878^4^, KX874259, KX872197, KX873492, FJ377668, KX872522^10,15^. PERU: Huánuco, *Peterson et al. 20367* (US), KX872839, KX873881^4^, KX874262, KX872200, KX873495, KX873128, KX872525^10,15^.

***Phalaris angusta*** Nees ex. Trin., BRAZIL: Caxias do Sul, *Scur 654* (US-3417995), KX872840, KX873882^1^, KX874263, KX872201, KX873496, KX873129, KX872526^15^. ***Phalaris aquatica*** L., USA: California, *Peterson et al. 19672* (CAN-593908), KX872842, KX873884^1^, KX874265, KX872203, KX873498, FJ377670, KX872528^15^; AUSTRALIA: Victoria, *Jobson 1942* (MELU-2023860), KX872843, KX873885^1^, KX874267, KX872204, KX873499, KX873130, KX872529^15^. ***Phalaris arundinacea*** L., CANADA: Alberta, *Peterson et al. 18383* (CAN-591065), KX872841, KX873883^1^, KX874264, KX872202, KX873497, FJ377669, KX872527^15^. ***Phalaris minor*** Retz., AUSTRALIA: Victoria, *Strudwick 689* (MELU-1580042), –, –, KX874266, –, –, KX873131, –. ***Phalaris paradoxa*** L., AUSTRALIA: Victoria, *Curtis 61* (MELU-2064584), MF327249, KX873886^1^, KX874268, KX872205, KX873500, KX873132, KX872530^15^; AUSTRALIA: Victoria, *McKenzie 95131* (MELU-2029457), MF327250, KX873887^1^, KX874269, KX872206, KX873501, KX873133, –.

***Poa gigantea*** (Tovar) Refulio, PERU: Ancash, *Peterson et al. 21680* (US), KX872844, KX873888, –, KX872207^8^, –, KX873134, KX872531^15^. ***Poa gymnantha*** Pilg., PERU: Ancash, *Peterson et al. 21733* (US), KX872845, KX873889, KX874270, KX872208^8^, –, KX873135, KX872532^15^. ***Poa pratensis*** L., USA: Utah, *Aurich & Forther* (US-3343370), KX872846, KX873890, KX874271, KX872209^8^, KX873502, FJ377677, KX872533^15^.

***Podagrostis aequivalvis*** (Trin.) Trin., CANADA: British Columbia, *Saarela & Percy 1307* (CAN-590686), KX872847, KX873554^6^, KX873946, KX871907, KX873503, KX873136, KX872262^14^.

***Polypogon australis*** Brongn., USA: California, *Peterson et al. 19678* (CAN-593905), KX872848, KX873891^6^, –, KX872210, KX873504, KX873137, KX872534^14^; Brongn., CHILE: Region V (Valparaiso), *Soreng & Soreng 7084* (US), FJ394583, KX873892^6^, KX874272, KX872211, KX873505, –, KX872535^14^; Region II (Antofagasta), *Peterson et al. 15551* (US-3445677), FJ394582, KX873893^6^, KX874273, KX872212, KX873506, FJ377671, KX872536^14^. ***Polypogon elongatus*** Kunth, MEXICO: Durango, *Peterson et al. 21182* (US), KX872603, KX873572^6^, KX873965, KX871926, KX873206, KX873138, KX872278^9,14^; Durango, *Peterson et al. 21234* (CAN-602189), KX872849, KX873894^6^, KX874274, KX872213, KX873507, KX873139, KX872537^9,14^; PERU: Ancash, *Peterson et al. 21688* (US), KX872850, KX873895^6^, KX874275, KX872214, KX873508, KX873140, KX872538^9,14^. ***Polypogon interruptus*** Kunth, PERU: Ancash, *Peterson et al. 21477* (US), KX872851, KX873896^6^, KX874276^7^, KX872215, KX873509, KX873141, KX872539^14^. ***Polypogon monspeliensis*** (L.) Desf., USA: California, *Peterson et al. 19669* (CAN-593869), –, KX873897^6^, KX874277, KX872216, KX873510, KX873142, KX872540^14^. ***Polypogon viridis*** (Gouan) Breistr., MEXICO: Coahuila, *Peterson et al. 20996* (US), KX872602, KX873571^6^, KX873964, KX871925, KX873205, KX873143, KX872277^14^; Mexico, *Peterson et al. 21309* (CAN-602364), KX872852, KX873898^6^, KX874278, KX872217, KX873511, KX873144, KX872541^14^; PERU: Ancash, *Peterson et al. 21470* (US), KX872853, KX873899^6^, KX874279, KX872218, KX873512, KX873145, KX872542^14^.

***Pseudobromus africanus*** (Hack.) Stapf., SOUTH AFRICA: Graskop, *Schweickerdt 6053* (US-2014104), KX872854, KX873900, –, –, KX873513, KX873146, KX872543^15^.

***Relchela panicoides*** Steud., ARGENTINA: Neuquén, *Peterson et al. 17364* (US-3451005), KX872855, KX873901, KX874280, KX872219, KX873514, KX873147, KX872544^14^; ARGENTINA: Rio Negro, *Peterson et al. 17334* (US-3450984), KX872856, KX873902, KX874281, KX872220, KX873515, KX873148, KX872545^14^.

***Rostraria cristata*** (L.) Tzvelev, ARGENTINA: Entre Ríos, *Renvoize 2968* (US-2962840), –, –, –, –, –, –, KX872546^10,12,15^. ***Rostraria pumila*** (Desf.) Tzvelev, KUWAIT: Shuwaikh, *Rawi 10915* (US-2970877), –, –, –, –, –, MF348280, KX872547^10,15^; AUSTRALIA: Western Australia, *Peterson & Rosenberg 14444* (US-3243163), KX872857, KX873903^4^, KX874284, KX872221, KX873516, KX873149, KX872548^10,15^; Western Australia, *Clarke 3442* (MELU-2293299), KX872858, –, KX874285, KX872222, KX873517, KX873150, KX872549^10,15^.

***Sphenopholis filiformis*** (Chapman) Scribner, USA: North Carolina, *Sorrie 11538* (US-3526553), KX872859, KX873904^4^, KX874286, –, KX873518, KX873151, KX872550^10,15^. ***Sphenopholis intermedia*** (Rydb.) Rydb., CANADA: Ontario, *Dugal & McRoy 4080* (CAN-581412), KX872860, KX873905^4^, KX874287, KX872223, KX873519, KX873152, KX872551^10,15^; Quebec, *Dugal & Camfield 4234* (CAN-581566), KX872861, KX873906^4^, KX874288, KX872224, KX873520, KX873153, KX872552^10,15^. ***Sphenopholis obtusata*** var. ***major*** (Torr.) K.S. Erdman, CANADA: Quebec, *Darbyshire & Oldham 3412* (CAN-529652), KX872862, KX873907^4^, KX874289, KX872225, KX873521, KX873154, KX872553^10,15^. ***Sphenopholis pensylvanica*** (L.) Hitchc., USA: Tennessee, *Sharp et al. 40056* (US-1981413), KX872863, KX873908^4^, KX874290, KX872226, KX873522, KX873155, KX872554^10,15^; Virginia, *Terrell 3644* (US-2465255), KX872864, KX873909^4^, KX874291, KX872227, KX873523, KX873156, KX872555^10,15^.

***Torreyochloa pallida*** var. ***pauciflora*** (J. Presl) J.I. Davis, CANADA: British Columbia, *Saarela & Percy 1187* (CAN-590622), KX872865, KX873910, KX874292, KX872228, KX873524, KX873157, KX872556^15^.

***Trisetum andinum*** Benth., ECUADOR: Pichincha, *Sklenar & Kosteckova 42438* (US-3338677), KX872866, KX873911^1,6^, KX874293^7^, KX872229, KX873525, KX873158, KX872557^10,15^. ***Trisetum cernuum*** subsp. ***canescens*** (Buckl.) Calder & R.L. Taylor, USA: California, *Peterson et al. 19686* (CAN-593914), –, KX873912*, KX874294, KX872230, KX873526, –, –; Oregon, *Peterson et al. 19720* (CAN-593845), KX872867, KX873913^4^, KX874295, KX872231, KX873527, KX873159, KX872558^10,15^; California, *Peterson et al. 19790* (CAN-593894), KX872868, KX873914^4^, KX874296, KX872232, KX873528, KX873160, KX872559^10,15^; California, *Peterson et al. 19667* (CAN-593892), –, KX873916^4^, KX874298, KX872234, KX873530, –, –. CANADA: British Columbia, *Peterson et al. 18723* (CAN-590880), KX872869, KX873915^4^, KX874297, KX872233, KX873529, KX873161, KX872560^10,15^. ***Trisetum distichophyllum*** (Vill.) Pal., SWITZERLAND: *Bührer* s.n. (CAN-207676), KX872870, KX873917^6^, KX874299, KX872235, KX873531, KX873162, KX872561^10,15^. ***Trisetum durangense*** Finot & P.M. Peterson, MEXICO: Durango, *Peterson & Saarela 21245* (CAN-602186), KX872871, KX873918^4^, KX874300, KX872236, KX873532, KX873163, KX872562^10,15^; Durango, *Peterson et al. 21217* (CAN 602508), KX872872, KX873919^4^, KX874301, KX872237, KX873533, KX873164, KX872563^10,15^. ***Trisetum flavescens*** (L.) P. Beauv., GERMANY: Hessen, *Kalheber 78-379* (CAN-433737), KX872873, KX873920^1,6^, KX874302, KX872238, KX873534, KX873165, KX872564^10,15^. ***Trisetum irazuense*** (Kuntze) Hitchc., VENEZUELA: Trujillo, *Stergios et al. 20561* (US-3449765), KX872874, KX873921^4^, KX874303, KX872239, KX873535, KX873166, KX872565^10,15^. ***Trisetum macbridei*** Hitchc., PERU: Ancash, *Peterson et al. 21679* (US), KX872875, KX873922^4^, KX874304, KX872240, –, KX873167, KX872566^10,15^. ***Trisetum montanum*** Vasey, CANADA: Alberta, *Peterson et al. 18397* (CAN-590867), KX872876, KX873923^1,6^, KX874305^7^, KX872241, KX873536, KX873168, KX872567^10,15^. ***Trisetum oreophilum*** Louise-Marie var. ***oreophilum***, ECUADOR: Chimborazo, *Peterson et al. 9239* (US-3237370), KX872878, KX873924^1,6^, KX874306^7^, KX872242, KX873537, KX873169, KX872568^10,15^. ***Trisetum oreophilum*** Louis-Marie, PERU: Lima, *Peterson et al. 20273* (US), KX872877, KX873925^1,6^, KX874307, KX872243, KX873538, KX873170, KX872569^10,15^. ***Trisetum palmeri*** Hitchc., MEXICO: Coahuila, *Hoge et al. 220* (US-3184578), KX872879, KX873926^4^, KX874308, –, KX873539, KX873171, KX872570^10,15^. ***Trisetum phleoides*** (d’Urv.) Kunth, ARGENTINA: Santa Cruz, *Peterson et al. 17256* (US-3453526), KX872880, KX873927^1,6^, KX874309, KX872244, KX873540, KX873172, KX872571^10,15^. ***Trisetum preslii*** (Kunth) E. Desv., ARGENTINA: Mendoza, *Peterson & Annable 11467* (US-3478841), KX872881, KX873928^1,6^, KX874310^7^, KX872245, KX873541, KX873173, KX872572^10,15^. ***Trisetum rosei*** Scribn. & Merr., MEXICO: Puebla, *Peterson et al. 21388* (CAN-602265), KX872882, KX873929^1,6^, KX874311^7^, KX872246, KX873542, KX873174, KX872573^10,15^. ***Trisetum spicatum*** (L.) K. Richt., PERU: Ancash, *Peterson et al. 17892* (US-03482379), KX872884, –, –, –, –, KX873176, KX872576^10,15^; Ancash, *Peterson et al. 21527* (US), KX872885, KX873933^1,6^, KX874313^7^, KX872249, –, KX873177, KX872577^10,15^; Ancash, *Peterson et al. 21542* (US), –, KX873934^1,6^, KX874314^7^, KX872250, –, –, –; Lima, *Peterson et al.* 20299 (US), KX872886, KX873935^1,6^, KX874315^7^, KX872251, KX873543, KX873178, KX872578^10,15^; AUSTRALIA: Victoria, *Chesterfield 3126* (MELU-2047036), KX872887, KX873936^1,6^, KX874316^7^, KX872252, KX873544, KX873179, KX872579^10,15^; Victoria, *Walsh et al. 6731* (MELU-2314238), KX872888, KX873937^1,6^, KX874317, –, KX873545, KX873180, KX872580^10,15^, Victoria, *H. van Rees 252* (MELU-598015) –, –, –, –, MF348275, –; USA: California, *Peterson et al. 19768* (CAN-593947), KX872889, KX873938^1,6^, KX874318, KX872253, KX873546, FJ377674, KX872581^10,15^. ***Trisetum spicatum*** (L.) K. Richt. var. ***spicatum***, PERU: Cajamarca, *Peterson et al. 21896* (US), MF327251, KX873932^1,6^, –, KX872248, –, KX873181, KX872575^10,15^. ***Trisetum spicatum*** var. ***cumingii*** (Nees ex. Steud.) Finot, PERU: La Libertad, *Peterson et al. 21934* (US), KX872883, KX873931^1,6^, KX874312^7^, KX872247, –, KX873175, KX872574^10,15^. ***Trisetum viride*** (Kunth) Kunth, MEXICO: Sinaloa, *Peterson & Saarela 21275* (CAN-602220), KX872890, KX873930^4^, KX874319, KX872254, KX873547, KX873182, KX872582^10,15^; Coahuila, *Peterson et al. 21134* (CAN-602537), KX872891, KX873939^4^, KX874320, KX872255, KX873548, KX873183, KX872583^10,15^; Coahuila, *Peterson & Valdes-Reyna 18783* (US-03496166), KX872892, KX873940^4^, KX874321, KX872256, KX873549, FJ377676, KX872584^10,15^. ***Trisetum virletii*** Fourn., MEXICO: Mexico, *Soto et al. 7926* (US-3428156), KX872893, KX873941^4^, KX874322, KX872257, KX873550, KX873184, KX872585^10,15^.

***Vahlodea atropurpurea*** (Wahlenb.) Fr. ex Hartm., CANADA: British Columbia, *Peterson et al. 18728* (CAN-590732), KX872894, KX873943, KX874324^7^, KX872259, KX873551, KX873185, KX872587^15^.

^1^ *psbK–psbI*: a 10–12 bp inversion, starting at position 382 of the alignment, flanked by a 10 bp inverted repeat. The inversion is 10 bp except in *Gaudinia fragilis*, in which it is 12 bp.

^2^ *psbK–psbI*: a two bp inversion flanked by a two bp inverted repeat.

^3^ *psbK–psbI*: a two bp inversion flanked by a seven bp inverted repeat.

^4^ *psbK–psbI*: a 298 bp deletion, starting at position 196 of the alignment. The deletion is 247 bp excluding gaps introduced into the alignment.

^5^ *psbK–psbI*: a 117 bp deletion, starting at position 76 of the alignment. The deletion is 84 bp excluding gaps in the alignment.

^6^ *psbK–psbI*: a 10 bp deletion, starting at position 289 of the alignment.

^7^ *psbA–rps19–trnH*: a six bp inversion, starting at position 507 of the alignment.

^8^ *atpF–atpH*: a 260–268 bp deletion varying in size at the 5’-end, starting at position 355 of the alignment.

^9^ ETS: a 62–66 bp insertion, starting at position 154 of the alignment.

^10^ ETS: a 12 bp deletion, starting at position 129 of the alignment.

^11^ ETS: a 220 bp insertion, starting at position 261 of the alignment.

^12^ ETS: an 81 bp insertion, starting at position 482 of the alignment.

^13^ ETS: an 86–192 bp insertion, starting at position 562 of the alignment.

^14^ ETS a 73 bp insertion (47–56 bp unaligned), starting at position 1273 of the alignment.

^15^ ETS: a 190 bp insertion when the outgroup is excluded, starting at position 752 of the alignment. The insertion is 107–159 bp excluding gapped sites when the outgroup is excluded. The indel in the outgroup, *Bromus vulgaris* is 472 bp (426 ungapped sites).

^16^ ETS: a 110 bp insertion, starting at position 1347 of the alignment.

## Supplementary materials

Supplementary material 1 Detailed voucher specimen information and GenBank accession numbers for newly sampled accessions Data type: Microsoft Excel file This dataset is made available under the Open Database License (http://opendatacommons.org/licenses/odbl/1.0/). The Open Database License (ODbL) is a license agreement intended to allow users to freely share, modify, and use this Dataset while maintaining this same freedom for others, provided that the original source and author(s) are credited.Jeffery M. Saarela, Roger D. Bull, Michel J. Paradis, Sharon N. Ebata, Paul M. Peterson, Robert J. Soreng, Beata Paszko

Supplementary material 2 GenBank accession numbers and publication information for previously published sequences included in the ITS, ETS, *matK* and *trnL–trnF* trees Data type: Microsoft Excel file This dataset is made available under the Open Database License (http://opendatacommons.org/licenses/odbl/1.0/). The Open Database License (ODbL) is a license agreement intended to allow users to freely share, modify, and use this Dataset while maintaining this same freedom for others, provided that the original source and author(s) are credited.Jeffery M. Saarela, Roger D. Bull, Michel J. Paradis, Sharon N. Ebata, Paul M. Peterson, Robert J. Soreng, Beata Paszko

Supplementary material 3 Maximum likelihood phylogram inferred from combined ITS+ETS sequence data Data type: Adobe PDF file Explanation note: ML bootstrap support is recorded along branches. No support is shown for branches with bootstrap support <50%. This dataset is made available under the Open Database License (http://opendatacommons.org/licenses/odbl/1.0/). The Open Database License (ODbL) is a license agreement intended to allow users to freely share, modify, and use this Dataset while maintaining this same freedom for others, provided that the original source and author(s) are credited.Jeffery M. Saarela, Roger D. Bull, Michel J. Paradis, Sharon N. Ebata, Paul M. Peterson, Robert J. Soreng, Beata Paszko

Supplementary material 4 Maximum likelihood phylogram inferred from ITS sequence data Data type: Adobe PDF file Explanation note: ML bootstrap support is recorded along branches. No support is shown for branches with bootstrap support <50%. Source information for previously published sequences is provided in Suppl. material 2. This dataset is made available under the Open Database License (http://opendatacommons.org/licenses/odbl/1.0/). The Open Database License (ODbL) is a license agreement intended to allow users to freely share, modify, and use this Dataset while maintaining this same freedom for others, provided that the original source and author(s) are credited.Jeffery M. Saarela, Roger D. Bull, Michel J. Paradis, Sharon N. Ebata, Paul M. Peterson, Robert J. Soreng, Beata Paszko

Supplementary material 5 Maximum likelihood phylogram inferred from ETS sequence data Data type: Adobe PDF file Explanation note: ML bootstrap support (left) and BI posterior probabilities (right) are recorded along branches. No support is shown for branches with bootstrap support <50% and posterior probability <0.5. Source information for previously published sequences is provided in Suppl. material 2. This dataset is made available under the Open Database License (http://opendatacommons.org/licenses/odbl/1.0/). The Open Database License (ODbL) is a license agreement intended to allow users to freely share, modify, and use this Dataset while maintaining this same freedom for others, provided that the original source and author(s) are credited.Jeffery M. Saarela, Roger D. Bull, Michel J. Paradis, Sharon N. Ebata, Paul M. Peterson, Robert J. Soreng, Beata Paszko

Supplementary material 6 Maximum likelihood phylogram inferred from combined plastid (*atpF–atpH*, *matK*, *psbA–rps19–trnH*, *psbK–psbI*, *trnL–trnF*) sequence data Data type: Adobe PDF file Explanation note: ML bootstrap support is recorded along branches. No support is shown for branches with bootstrap support <50%. This dataset is made available under the Open Database License (http://opendatacommons.org/licenses/odbl/1.0/). The Open Database License (ODbL) is a license agreement intended to allow users to freely share, modify, and use this Dataset while maintaining this same freedom for others, provided that the original source and author(s) are credited.Jeffery M. Saarela, Roger D. Bull, Michel J. Paradis, Sharon N. Ebata, Paul M. Peterson, Robert J. Soreng, Beata Paszko

Supplementary material 7 Maximum likelihood phylogram inferred from *matK* sequence data Data type: Adobe PDF file Explanation note: ML bootstrap support is recorded along branches. No support is shown for branches with bootstrap support <50%. Source information for previously published sequences is provided in Suppl. material 2. This dataset is made available under the Open Database License (http://opendatacommons.org/licenses/odbl/1.0/). The Open Database License (ODbL) is a license agreement intended to allow users to freely share, modify, and use this Dataset while maintaining this same freedom for others, provided that the original source and author(s) are credited.Jeffery M. Saarela, Roger D. Bull, Michel J. Paradis, Sharon N. Ebata, Paul M. Peterson, Robert J. Soreng, Beata Paszko

Supplementary material 8 Maximum likelihood phylogram inferred from *atpF–atpH* sequence data Data type: Adobe PDF file Explanation note: ML bootstrap support is recorded along branches. No support is shown for branches with bootstrap support <50%. This dataset is made available under the Open Database License (http://opendatacommons.org/licenses/odbl/1.0/). The Open Database License (ODbL) is a license agreement intended to allow users to freely share, modify, and use this Dataset while maintaining this same freedom for others, provided that the original source and author(s) are credited.Jeffery M. Saarela, Roger D. Bull, Michel J. Paradis, Sharon N. Ebata, Paul M. Peterson, Robert J. Soreng, Beata Paszko

Supplementary material 9 Maximum likelihood phylogram inferred from *psbK–psbI* sequence data Data type: Adobe PDF file Explanation note: ML bootstrap support is recorded along branches. No support is shown for branches with bootstrap support <50%. This dataset is made available under the Open Database License (http://opendatacommons.org/licenses/odbl/1.0/). The Open Database License (ODbL) is a license agreement intended to allow users to freely share, modify, and use this Dataset while maintaining this same freedom for others, provided that the original source and author(s) are credited.Jeffery M. Saarela, Roger D. Bull, Michel J. Paradis, Sharon N. Ebata, Paul M. Peterson, Robert J. Soreng, Beata Paszko

Supplementary material 10 Maximum likelihood phylogram inferred from *psbA–rps19–trnH* sequence data Data type: Adobe PDF file Explanation note: ML bootstrap support is recorded along branches. No support is shown for branches with bootstrap support <50%. This dataset is made available under the Open Database License (http://opendatacommons.org/licenses/odbl/1.0/). The Open Database License (ODbL) is a license agreement intended to allow users to freely share, modify, and use this Dataset while maintaining this same freedom for others, provided that the original source and author(s) are credited.Jeffery M. Saarela, Roger D. Bull, Michel J. Paradis, Sharon N. Ebata, Paul M. Peterson, Robert J. Soreng, Beata Paszko

Supplementary material 11 Maximum likelihood phylogram inferred from *trnL–trnF* sequence data Data type: Adobe PDF file Explanation note: ML bootstrap support is recorded along branches. No support is shown for branches with bootstrap support <50%. Source information for previously published sequences is provided in Suppl. material 2. This dataset is made available under the Open Database License (http://opendatacommons.org/licenses/odbl/1.0/). The Open Database License (ODbL) is a license agreement intended to allow users to freely share, modify, and use this Dataset while maintaining this same freedom for others, provided that the original source and author(s) are credited.Jeffery M. Saarela, Roger D. Bull, Michel J. Paradis, Sharon N. Ebata, Paul M. Peterson, Robert J. Soreng, Beata Paszko

Supplementary material 12 Combined ITS+ETS alignment file Data type: NEXUS format This dataset is made available under the Open Database License (http://opendatacommons.org/licenses/odbl/1.0/). The Open Database License (ODbL) is a license agreement intended to allow users to freely share, modify, and use this Dataset while maintaining this same freedom for others, provided that the original source and author(s) are credited.Jeffery M. Saarela, Roger D. Bull, Michel J. Paradis, Sharon N. Ebata, Paul M. Peterson, Robert J. Soreng, Beata Paszko

Supplementary material 13
ITS alignment file Data type: NEXUS format This dataset is made available under the Open Database License (http://opendatacommons.org/licenses/odbl/1.0/). The Open Database License (ODbL) is a license agreement intended to allow users to freely share, modify, and use this Dataset while maintaining this same freedom for others, provided that the original source and author(s) are credited.Jeffery M. Saarela, Roger D. Bull, Michel J. Paradis, Sharon N. Ebata, Paul M. Peterson, Robert J. Soreng, Beata Paszko

Supplementary material 14
ETS alignment file Data type: NEXUS format This dataset is made available under the Open Database License (http://opendatacommons.org/licenses/odbl/1.0/). The Open Database License (ODbL) is a license agreement intended to allow users to freely share, modify, and use this Dataset while maintaining this same freedom for others, provided that the original source and author(s) are credited.Jeffery M. Saarela, Roger D. Bull, Michel J. Paradis, Sharon N. Ebata, Paul M. Peterson, Robert J. Soreng, Beata Paszko

Supplementary material 15 Combined plastid (*atpF–atpH*, *matK*, *psbA–rps19–trnH*, *psbK–psbI*, *trnL–trnF*) alignment file Data type: NEXUS format This dataset is made available under the Open Database License (http://opendatacommons.org/licenses/odbl/1.0/). The Open Database License (ODbL) is a license agreement intended to allow users to freely share, modify, and use this Dataset while maintaining this same freedom for others, provided that the original source and author(s) are credited.Jeffery M. Saarela, Roger D. Bull, Michel J. Paradis, Sharon N. Ebata, Paul M. Peterson, Robert J. Soreng, Beata Paszko

Supplementary material 16
*atpF–atpH* alignment file Data type: NEXUS format This dataset is made available under the Open Database License (http://opendatacommons.org/licenses/odbl/1.0/). The Open Database License (ODbL) is a license agreement intended to allow users to freely share, modify, and use this Dataset while maintaining this same freedom for others, provided that the original source and author(s) are credited.Jeffery M. Saarela, Roger D. Bull, Michel J. Paradis, Sharon N. Ebata, Paul M. Peterson, Robert J. Soreng, Beata Paszko

Supplementary material 17
*psbK–psbI* alignment file Data type: NEXUS format This dataset is made available under the Open Database License (http://opendatacommons.org/licenses/odbl/1.0/). The Open Database License (ODbL) is a license agreement intended to allow users to freely share, modify, and use this Dataset while maintaining this same freedom for others, provided that the original source and author(s) are credited.Jeffery M. Saarela, Roger D. Bull, Michel J. Paradis, Sharon N. Ebata, Paul M. Peterson, Robert J. Soreng, Beata Paszko

Supplementary material 18
*psbA–rps19–trnH* alignment file Data type: NEXUS format This dataset is made available under the Open Database License (http://opendatacommons.org/licenses/odbl/1.0/). The Open Database License (ODbL) is a license agreement intended to allow users to freely share, modify, and use this Dataset while maintaining this same freedom for others, provided that the original source and author(s) are credited.Jeffery M. Saarela, Roger D. Bull, Michel J. Paradis, Sharon N. Ebata, Paul M. Peterson, Robert J. Soreng, Beata Paszko

Supplementary material 19
*trnL–trnF* alignment file Data type: NEXUS format This dataset is made available under the Open Database License (http://opendatacommons.org/licenses/odbl/1.0/). The Open Database License (ODbL) is a license agreement intended to allow users to freely share, modify, and use this Dataset while maintaining this same freedom for others, provided that the original source and author(s) are credited.Jeffery M. Saarela, Roger D. Bull, Michel J. Paradis, Sharon N. Ebata, Paul M. Peterson, Robert J. Soreng, Beata Paszko

Supplementary material 20
*matK* alignment file Data type: NEXUS format This dataset is made available under the Open Database License (http://opendatacommons.org/licenses/odbl/1.0/). The Open Database License (ODbL) is a license agreement intended to allow users to freely share, modify, and use this Dataset while maintaining this same freedom for others, provided that the original source and author(s) are credited.Jeffery M. Saarela, Roger D. Bull, Michel J. Paradis, Sharon N. Ebata, Paul M. Peterson, Robert J. Soreng, Beata Paszko
