# Supplementary material for: Molecular phylogenetics of cool-season grasses in the subtribes Agrostidinae, Anthoxanthinae, Aveninae, Brizinae, Calothecinae, Koeleriinae and Phalaridinae (Poaceae, Pooideae, Poeae, Poeae chloroplast group 1)
Source: PhytoKeys. 2017 Oct 9;(87):1–139. doi: 10.3897/phytokeys.87.12774 (PMC5672130; doi:10.3897/phytokeys.87.12774)
Supplement: Supplementary material 10 — Maximum likelihood phylogram inferred from psbA–rps19–trnH sequence data [file phytokeys-87-001-s010.pdf]

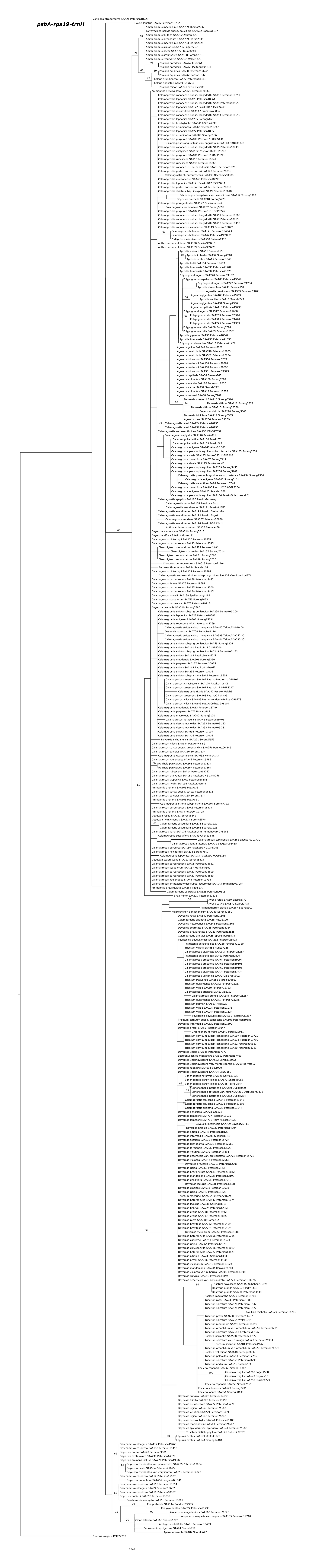
psbA-rps19-trnH
